# Pushing the limits of the hydrogen bond enhanced halogen bond—the case of the C–H hydrogen bond†

**DOI:** 10.1039/d2sc03792k

**Published:** 2022-08-31

**Authors:** Daniel A. Decato, Jiyu Sun, Madeleine R. Boller, Orion B. Berryman

**Affiliations:** University of Montana 32 Campus Drive Missoula MT USA orion.berryman@umontana.edu

## Abstract

C–H hydrogen bonds have remarkable impacts on various chemical systems. Here we consider the influence of C–H hydrogen bonds to iodine atoms. Positioning a methyl group between two iodine halogen bond donors of the receptor engendered intramolecular C–H hydrogen bonding (HBing) to the electron-rich belt of both halogen bond donors. When coupled with control molecules, the role of the C–H hydrogen bond was evaluated. Gas-phase density functional theory studies indicated that methyl C–H hydrogen bonds help bias a bidentate binding conformation. Interaction energy analysis suggested that the charged C–H donors augment the halogen bond interaction—producing a >10 kcal mol^−1^ enhancement over a control lacking the C–H⋯I–C interaction. X-ray crystallographic analysis demonstrated C–H hydrogen bonds and bidentate conformations with triflate and iodide anions, yet the steric bulk of the central functional group seems to impact the expected trends in halogen bond distance. In solution, anion titration data indicated elevated performance from the receptors that utilize C–H Hydrogen Bond enhanced Halogen Bonds (HBeXBs). Collectively, the results suggest that even modest hydrogen bonds between C–H donors and iodine acceptors can influence molecular structure and improve receptor performance.

## Abstract 2

C–H hydrogen bonds to iodine halogen bond donors are shown to improve halogen bonding and molecular preorganization.

## Graphical abstract

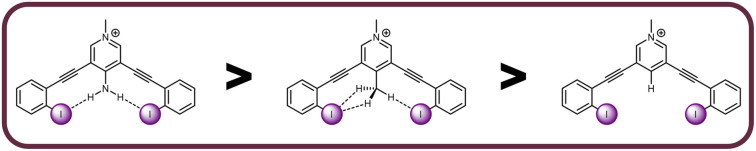

## Introduction

**Fig. 1:**
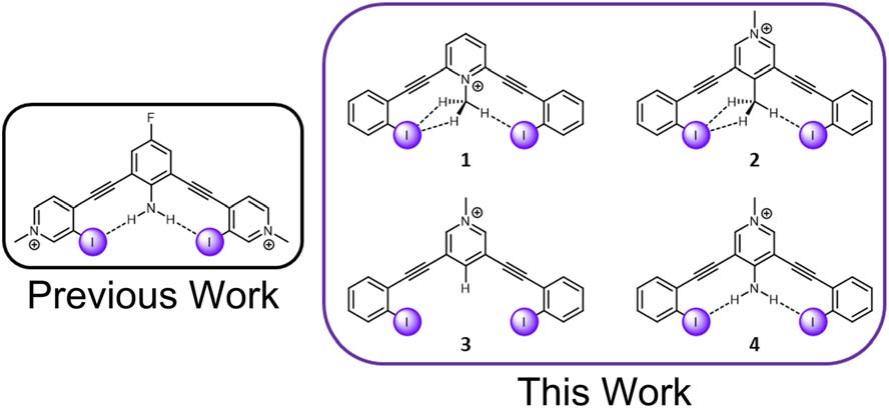
A previously evaluated amine HBeXB scaffold (left). The receptors in this work designed to evaluate C–H ‘non-traditional’ HBeXB (right).

C–H hydrogen bonds are often awarded the epithet “weak,” despite showing remarkable function in diverse chemical fields.^1^ The growing appreciation for these ‘non-traditional’ hydrogen bonds is reflected in the modern definition of the hydrogen bond, which places an emphasis on *evidence of bond formation*.^2^ While C–H hydrogen bond donors are now widely appreciated,^3,4^ their interaction with weak acceptors is seldomly studied. π-acceptors are the most frequently evaluated, and have been the subject of both structural chemistry^5^ and structural biology reports.^6^ Sulfur C–H hydrogen bond acceptors have more recently come into focus.^7^ Terminal organohalogens are also considered weak hydrogen bond acceptors despite being electronegative functional groups in polar covalent bonds.^8^ Within the group 17 elements, fluorine acceptors have been the primary focus of study with both traditional hydrogen bond donors and C–H hydrogen bond donors.^9–15^ In contrast, larger halogens with the capacity to be strong halogen bond donors, have been largely unexplored.^16^‡‡Historically, evaluations of CH HBing to organic halogens have focused on various conformational analysis studies highlighting a preference for gauche conformations over anti in alkyl halides. For a relevant review see: *Chem. Rev.* 2010, 110, 10, 6049–6076. Considering over half of organohalogen drugs launched contain heavier halogens with the faculty for halogen bonding (X = Cl, Br, I),^17^ this deficiency should be addressed. Herein, we evaluate the capacity of C–H hydrogen donors to hydrogen bond with iodine atoms by measuring the performance of halogen bonding anion receptors (Fig. 1).

The halogen bond is a noncovalent interaction between an electrophilic halogen and a Lewis base^18^ that can contain elements of covalency, polarization and electrostatics.^19^ The interaction has appealed to fundamental and functional chemical disciplines, in part for its strict linear geometry—which is far more stringent than the hydrogen bond. From an electrostatic perspective, halogen bond directionality is most often attributed to an anisotropic distribution of electron density that develops on an electron-deficient halogen. The electronic redistribution results in an electropositive region at the tip of the halogen and an electron rich belt orthogonal to the covalent bond. The electropositive region (the σ-hole) justifies the attractive interaction between the halogen and Lewis bases. The electron rich region is largely responsible for the linearity of the interaction (as a Lewis base deviates from the tip of the halogen the interaction becomes less favorable and eventually becomes repulsive) and various attractive “side-on” interactions with electrophilic species such as metals.^20^

More recently, this electronegative region of a terminal organohalogen has been utilized as a hydrogen bond acceptor while simultaneously donating a halogen bond—*a hydrogen bond-enhanced halogen bond* (HBeXB).^21^ A hydrogen bond to the electronegative belt of a halogen bond donor further polarizes and strengthens the halogen bond donor. HBeXBs have influenced macromolecule stability,^22^ small molecule anion binding,^23^ organocatalysis,^24,25^ and have been quantified in a fundamental solution study.^16^ Each of these studies employ “traditional” –OH or –NH donors, prompting us to consider the efficacy of C–H hydrogen bond donors to operate in a similar manner. To test this, we have constructed a series of charge-assisted bidentate halogen bond receptors to evaluate C–H HBeXBing in solution, the solid-state, and *in silico*.

## Results and discussion

### Design considerations and synthesis

**Fig. 2:**
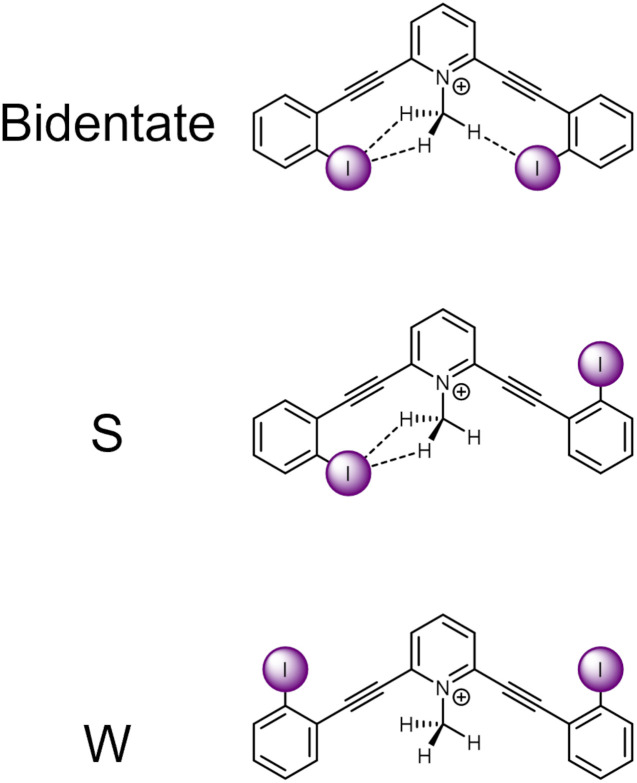
ChemDraw depictions of 1 highlighting the three planar conformations of *meta*-bis-ethynyl core receptors.

The current receptor design was inspired by our previous studies that established the HBeXB. Here, a bidentate receptor with two iodopyridinium arms flanking an aniline core afforded hydrogen bonds directed at the electron rich belt of the iodine atoms (Fig. 1, left).^23^ The *meta*-bis-ethynyl core enables three planar conformations—bidentate, S, and W (Fig. 2). The hydrogen bonds from the amine preorganized the receptor into the bidentate conformation and augmented the halogen bond donors, affording a near 9-fold increase in halide binding over a control molecule without the –NH_2_ donor. With a slight redesign, we envisioned that this system could be used to evaluate C–H hydrogen bonding to iodine halogen bond donors (Fig. 1, right). Simply put, could methyl C–H hydrogen bond donors operate like the previously studied NH_2_ to preorganize the receptor and improve anion binding?

To evaluate this hypothesis, four bidentate halogen bond receptors with a bis-ethynyl pyridinium core and flanking benzene arms were constructed (Fig. 1, see ESI† for synthesis details). The substitution on each core was varied, resulting in two receptors that could C–H HBeXB (1 & 2), a proto-control (3), and an amine (NH_2_ HBeXB) control (4) (Fig. 1). The pyridinium core of the receptors served as an electron withdrawing group to: enhance the halogen bond donor strength, produce potent C–H hydrogen bond donors in 1 & 2, and ensure the presence of an anion in solid-state evaluations. The *N*-methylpyridinium of 1—directed toward the receptor binding pocket—would enable C–H hydrogen bonding to the halogen bond donors when in the bidentate conformation. We hypothesized that receptor 1 should offer stronger halogen bonding than 2 or 3 due to the location of the pyridinium (greater through bond and through space effect on the halogen bond). In scaffolds 2 and 3, the pyridinium methyl functionality was placed on the backside of the receptor, removed from the binding pocket. Receptor 2 featured a methyl group *para* to the pyridinium nitrogen, directed into the binding pocket to evaluate C–H hydrogen bonding to the halogen bond donors. Scaffold 3 is a control molecule of 2 where the methyl group was replaced with a hydrogen atom. Scaffold 4 is structurally like 2 and 3 with the pyridinium methyl group directed away from the pocket but has an internally directed –NH_2_ group—included to benchmark the C–H HBeXBing.

### Computational evaluations

To evaluate the influence of C–H hydrogen bonding on scaffolds 1–4 computational investigations were carried out using Gaussian 09 at the M06-2X/def2-TZVPP level of theory and the small-core energy-consistent relativistic effective core potential (def2-ECP) applied to iodine (for additional computational details see ESI†).

### Conformational analysis

An initial relaxed scan dihedral driver analysis (Fig. 34S†) suggested that C–H hydrogen bonding to the iodine atoms stabilize the bidentate conformation. Thus, we obtained single-point energy computations for 1–4 in the bidentate, W, and S conformations (Table 1 for values, Fig. 2 for depictions of conformations).

**Table 1:** Computational results^a^

Scaffold	*V*_s,max_ b	IE	Relative IE	Favored conformation	Relative energy single point conformational analysis		
					Bidentate	S	W
1	75.93	−77.36	—	Bidentate	0.00	0.93	2.29
2	68.40	−70.37^c^	6.99^c^	S	0.04	0.00	0.49
3	69.65	−67.11	10.25	W	0.68	0.34	0.00
4	75.30	−76.70	0.66	Bidentate	0.00	2.34	4.82

a All values are presented in kcal mol^−1^. Interaction energy (IE) is computed as the difference between the complex and the isolated constituents in the same geometry as the complex. Values were corrected for basis set superposition error using the counterpoise technique (see ESI for more details).

b *V*_s,max_ value taken from surface of iodine atom when the receptor is in the bidentate conformation.

c The interaction energy was taken from a transition state structure with one imaginary frequency.

Scaffold 1 clearly prefers the bidentate conformation over the S and W conformation by 0.93 and 2.29 kcal mol^−1^, respectively. The favorable bidentate binding mode is attributed to C–H hydrogen bonding to the iodine atoms. In contrast scaffold 2, containing less electron deficient C–H hydrogen bond donors, very slightly favors the S over the bidentate conformation by 0.04 kcal mol^−1^. We note that sterics may be a source of the S conformation being slightly favored here and was further suggested in the solid-state investigations (*vide infra*). However, the W conformation of 2 is nearly 0.5 kcal mol^−1^ higher in energy than the S conformation. The relative preference for 1 to adopt the bidentate conformation highlights the impact of the stronger C–H hydrogen bond and favourable molecular dipoles. Control molecule 3, lacking the C–H hydrogen bond donor, favors the W conformation by 0.34 kcal mol^−1^ over the S conformation. Notably, the bidentate conformation of 3 is 0.68 kcal mol^−1^ less stable than the favored W conformation. 4 was evaluated to compare the conformational preference for a receptor containing a more traditional N–H hydrogen bond donor. 4 favors the bidentate conformation by 2.34 and 4.82 kcal mol^−1^ over the S and W conformation, respectively. The data indicate the stronger N–H hydrogen bonds provide more preorganization within this system. Overall, the conformational analysis highlights that C–H hydrogen bonding to the iodine halogen bond donors can stabilize the convergent bidentate conformation.

### Electrostatic potential analysis

Next, we asked whether C–H hydrogen bond donors could be used to enhance the strength of the halogen bond donor. Molecular electrostatic potential (MEP) maps of 1–4 provided an estimate of halogen bond strength by assessing the σ-hole (*V*_s,max_) of the iodine donors when adopting the bidentate conformation (Table 1).

1 had the greatest *V*_s,max_ when compared to the other analogues with a value of 75.93 kcal mol^−1^ due to the charge-assisted C–H hydrogen bond and the electron withdrawing effects associated with the location of the pyridinium. In contrast, 2 and 3 had similar *V*_s,max_ values (68.40 and 69.65 kcal mol^−1^, respectively). If C–H HBeXBing was enhancing the halogen bond donor in 2, we would expect 2 to have a greater *V*_s,max_ value. One possible explanation for this observation is that the electron donating effects of the methyl group nullified any polarization afforded by the C–H hydrogen bonds. Although a recent paper discussing distance and substituent effects indicates this may be negligible.^26^ The similar *V*_s,max_ values of 2 & 3 may suggest a potential limit to the C–H HBeXB and that receptor differences in solution between these two could be dictated by preorganization effects. Molecule 4 has the second greatest *V*_s,max_ (75.30 kcal mol^−1^) of the receptors evaluated, nearly 7 kcal mol^−1^ greater than 2 and 3, confirming that a stronger hydrogen bond donor will elicit greater σ-hole augmentation.

### Interaction energy analysis

Halogen bond interaction energies (see ESI† for details) with iodide were also computed to gather a more complete assessment of C–H HBeXB augmentation beyond electrostatics. The iodide counteranion was evaluated to complement the solid-state studies. Trending with the MEP data, 1 had the greatest interaction energy with iodide (−77.36 kcal mol^−1^). Receptor 4 had the second strongest interaction energy which was 0.66 kcal mol^−1^ less than 1. The results of 1 and 4 was expected considering the electronics of 1 and the strengths of the hydrogen bond donors in 1 and 4.

The gas phase interaction energies of 2 and 3 with iodide contrasts with the σ-hole (*V*_s,max_) analysis. Receptor 2 had a 3.26 kcal mol^−1^ greater interaction energy than 3, suggesting that the methyl C–H hydrogen bond donors strengthen the halogen bond.§§Multiple minimizations were conducted on the complex 2·I^−^ and each time the receptor adopted a distorted receptor conformation resulting in two halogen bonds and a C–H hydrogen bond to the iodine (Fig. 35S†). This tridentate structure would not permit valid interaction energy comparison as it would be comprised of two halogen bonds and the hydrogen bond. The intermediate structure had an imaginary frequency more positive than −50 cm^−1^. This disparity with the MEP analysis also provides another example where *V*_s,max_ σ-hole analysis may lead to incorrect predictions in halogen bond strength.^27^

### Atoms in molecules (AIM) analysis

Facilitated by Multiwfn,^28^ Bader's AIM analysis^29^ provided additional evidence for an intramolecular hydrogen bond between the CH_3_ methyl group and the iodine halogen bond donors.¶¶The shortcomings of the AIM method are noted. Those interested are referred to: *J. Comput. Chem.* 2019, 40, 2868–2881. With 1 and 2 there are (3,−1) bond critical points (BCPs) and bond paths (BPs) between the methyl group and the iodine atoms, suggesting a bonding relationship. Scaffolds 1 and 2 have two unique BPs—due to the geometry of the receptor and the methyl C–H hydrogen bond donors. In one case, there is a BP running directly from the hydrogen atom to the iodine in the same plane suggesting the presence of a monodentate C–H⋯I–C hydrogen bond. In the other case the BP splits the two hydrogen atoms and runs from the parent carbon atom to the iodine (Fig. 36S†). Previous evaluations of methyl systems with traditional hydrogen bond acceptors suggest an interplay between carbon bonding (*i.e.*, a tetrel bond) and what is more commonly asserted as a bifurcated (or trifurcated) hydrogen bond.^30,31^ These reports when compared to our BP findings suggest the BP may be considered a carbon tetrel bond, yet the angle between the donor and acceptor doesn't fit the tight criteria suggested for identifying these tetrel bonds in the solid-state.^30^ Furthermore it has been noted with an oxygen acceptor that the C–H⋯O hydrogen bond is comparable in binding energy to the C⋯O tetrel bond.^32^ The geometry presented here represents an opportunity for further carbon tetrel bond investigation and classification. Regardless, the BPs identified here provide additional evidence of C–H⋯I–C hydrogen bonds.

In contrast, 3 shows no BCP or BP between the aryl CH proton and the iodine donors. As expected, 4 has BCPs and BPs between the amine protons and the iodine atoms, aligning with a previous AIM HBeXB study evaluating intramolecular amide hydrogen bond donors.^33^ Overall, these theoretical data indicate that C–H hydrogen bonding to iodine atoms is occurring which would aid in receptor preorganization. The *in silico* data also suggests that the halogen bond is enhanced by the hydrogen bond but further physical studies in the solid and solution state are required.

### Solid-state evaluations

**Fig. 3:**
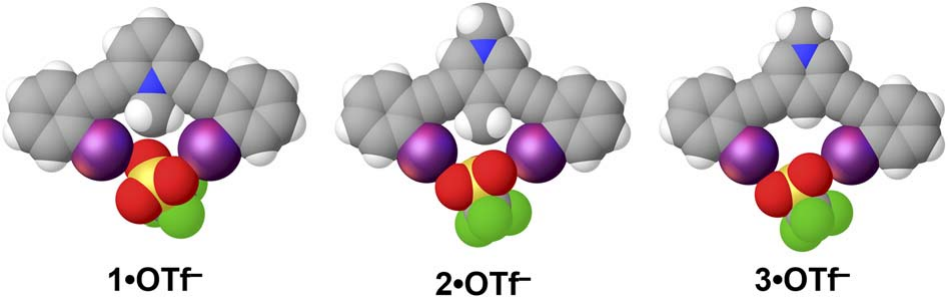
Asymmetric units of the triflate structures of 1–3. Each receptor adopts the bidentate conformation and halogen bonds to different triflate oxygen atoms. Spheres drawn using the default vdW radii within Olex2.

The triflate (OTf^−^) salts of 1–4 were synthesized and crystallized to provide an initial assessment of preorganization and C–H HBeXB in the solid state (Fig. 3). 1·OTf^−^, produced the shortest halogen bond contacts with OTf^−^ (*R*_IO_ values of 0.84 and 0.85) (Table 2)—highlighting the ability of the charged C–H donor to form HBeXBs. The structures of 2·OTf^−^ (*R*_IO_ values of 0.86 and 0.93) and 3·OTf^−^ (*R*_IO_ values of 0.88 and 0.91) have halogen bond contacts that are longer than 1·OTf^−^ which agrees with the computational evaluations on interaction strength. Between these two receptors 2·OTf^−^ exhibits both the longest and shortest halogen bond contacts whereas 3·OTf^−^ without C–H HBeXB has contacts that are intermediate (Table 2). The OTf^−^ anion has multiple Lewis basic sites and in each of the three complexes each iodine halogen bond donor forms a monodentate contact with distinct oxygen atoms. This binding complicates the analysis as there are subtle differences in the arrangement of the OTf^−^ despite all three complexes crystalizing in *P̄*1 with *Z*′ = 1 and adopting the bidentate conformation.

**Table 2:** Table of germane halogen bonding structural parameters

Complex	Distance (Å)	Angle (°)	*R*_XA_ a
1·I^−^	3.5694(4)	176.25(5)	0.87
1·I^−^	3.5772(3)	176.99(6)	0.88
2·I^−^	3.7037(5)	174.72(11)	0.91
3·I^−^	3.6590(4)	176.96(6)	0.90
4·I^−^	3.6247(5)	175.46(11)	0.89
1·OTf^−^	2.985(3)	177.60(10)	0.84
1·OTf^−^	3.017(2)	160.12(11)	0.85
2·OTf^−^	3.062(5)	175.31(11)	0.86
2·OTf^−^	3.299(5)	160.6(2)	0.93
3·OTf^−^	3.113(2)	170.43(9)	0.88
3·OTf^−^	3.219(3)	170.19(8)	0.91
4·OTf^−^b	3.003(11)	169.9(3)	0.85

a *R*_XA_ is the reduction ratio which is defined as 
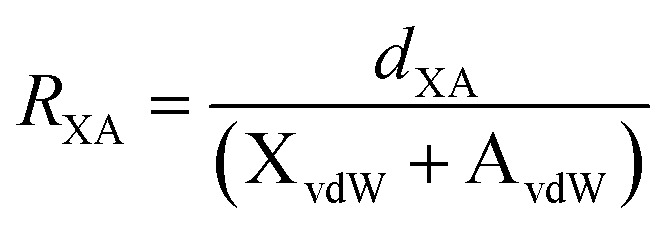
 where *d*_XA_ is the measured distance (Å) from the halogen donor (X) to the acceptor (A), divided by the sum of the van der Waals radii (Å) of X and A (X_vdW_ + A_vdW_). van der Waals radii used from Alvarez.^35^

b In 4·OTf^−^ the anion is disordered and the values shown are measured from the major component.

Interestingly, 4·OTf^−^ crystallizes in the tetragonal space group *P̄*42_1*c*_ and adopts an S conformation with a *Z*′ = 1 (Fig. S1†). We suspect that the unique shape of the triflate anion contributed to the deviation from the bidentate conformation as the anion ends up being bound to the receptor in a tridentate manner by an aryl C–H hydrogen bond, an N–H hydrogen bond and a halogen bond—a conformation previously observed in a dicationic receptor.^23^

**Fig. 4:**
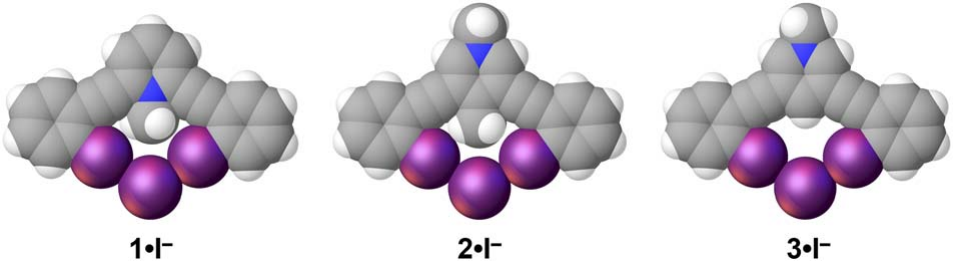
Iodide structures of 1–3. Each receptor adopts a bidentate conformation. Spheres drawn using the default vdW radii within Olex2.

To limit the influences of the polyatomic anion we crystalized structures 1–4 with monoatomic iodide (Fig. 4). Paralleling the OTf^−^ complexes, 1·I^−^ crystalized in the space group *P̄*1 with a *Z*′ = 1 resulting in the shortest halogen bond contacts with *R*_II_ values of 0.87 and 0.88 (Table 2). The structures of 2–4 with iodide all crystalized in *Pbcn* with a *Z*′ = 0.5. The isomorphous structures offer a favorable opportunity to evaluate potential influence of C–H HBeXB. The crystallographic symmetry dictates a single unique C–I⋯I^−^ contact. 2·I^−^ and 3·I^−^ had halogen bond distances and angles of 3.7037(5) Å, 174.72(11)° and 3.6590(4) Å, 176.96(6)°, respectively. The ≈0.04 Å shorter halogen bond contact of 3·I^−^, without any C–H hydrogen bond donors directed to the iodine rich belt of the halogen bond donor, potentially suggests the lower limit of C–H HBeXB or that electron donating effects of the methyl group are influencing the contact distance. Alternatively, the steric bulk of the methyl group of 2 might be preventing the alkynes from bending as much, thereby inhibiting shorter halogen bond contacts. For example, the iodine-to-iodine distance in 2·I^−^ is 6.3882(5) Å while in 3·I^−^ the distance is 6.1792(5) Å. The alkyne distortion is further demonstrated by measuring the angle formed by the centroids of each ring (arm-core-arm angle) of the receptor—a smaller angle would indicate the alkynes clamping down on the anion. In 2·I^−^ this angle is 124.559(3)° whereas 3·I^−^ is reduced to an angle of 123.025(4)°. So, it is possible that the C–H hydrogen bond donors from the methyl group enhance the halogen of 2 yet also introduce steric hindrance.

The crystal structure of 4·I^−^ offered another opportunity to compare the intramolecular hydrogen bond as this structure was also isomorphous with 2·I^−^ and 3·I^−^ (Fig. S1†). 4·I^−^ had halogen bond distances and angles of 3.6247(5) and 175.46(11). The halogen bonds of 4·I^−^ were shorter than both the 2 and 3 iodide structures which correlates with both the MEP and interaction energy analysis. The iodine-to-iodine distance was 6.2764(10)Å which represents a midpoint between 2·I^−^ and 3·I^−^, further suggesting that a central hydrogen bond donating group, whether that is a NH_2_ or a CH_3_, may limit the ability of the receptor to distort in this system.||||1·I has a iodine-to-iodine distance of 6.0546(4) and the angle of 121.002(19). These parameters do not follow the pattern suggested. We believe there are several possibilities for why 1·I does not follow the trend. First is that the structure crystalized in a different space group resulting in different packing. Second is that the location of the pyridinium is different. Thus, the halogen bond is more potent and possibly overcomes some of the steric clashing penalties imposed by the central methyl group. The angle formed by the centroids of each ring of the receptor was 123.94(4)° and is a midpoint between 2·I^−^ and 3·I^−^ following the trends of steric size (*i.e.* H < NH_2_ < Me).^34^

### C–H hydrogen bonds

The geometry and the rigid directional nature of the *meta*-bis-ethynyl core enables 1 and 2 to display C–H hydrogen bonds to the iodine atoms. In the presented structures of 1 and 2, the methyl groups maintain a single “strong to moderate” C–H hydrogen bond with the iodine as well as two “weak” CH hydrogen bonds—based on the parameters outlined by Johnson, Haley, Pluth *et al.* in a study focused mainly on CH hydrogen bonding to sulfur species.^7^ In their study the authors also expanded their analysis to other acceptors including organic iodine in a Cambridge Structural Database (CSD) analysis. The distances and angles of the strong to moderate C–H hydrogen bonds of 1 and 2 fall within regions that have the greatest number of observations in this reported CSD study. In contrast, the weak Hydrogen bonds fall in a region where C–H contacts trend, albeit fewer observations are noted. These geometries, coupled with the findings of the aforementioned CSD search is further indication that C–H⋯I–C are operating within this system.

### Solution studies

Our theoretical analysis and the solid-state investigations suggested that C–H HBeXB may improve anion receptor performance. To test this hypothesis in solution ^1^H NMR titrations were carried out to obtain association constants. However, minimal shifting of ^1^H resonances upon introduction of anions as tetrabutylammonium salts dictated the use of other spectroscopic methods.**Early efforts used the triflate salts of 1–4 as the starting host species. To ensure solubility, DMSO-*d*_*6*_ was the media investigated with various tetra-*n*-butylammonium anion sources. Unfortunately, minimal shifting of the ^1^H NMR resonances necessitated modifications. An initial consideration was that the OTf^−^ anion was potentially outcompeting the iodide anion, leading to minimal changes in chemical shift. However, the PF_6_^−^ and the BArF^−^ complexes 1 also showed minimal shifting upon addition of different anions. We then considered that a derivative with a –CF_3_ group *para* to the iodine donors might enhance binding (additional electron withdrawing) and potentially improve solubility. Unfortunately, CF_3_–1·OTf^−^ displayed the same minimal shifting and exhibited minimal solubility enhancements when evaluating other solvents. Presumably the DMSO solvent leads to weaker binding *vs.* the ultimate solvent choice of THF/DMSO/H_2_O (90/9.9/0.1) in the UV-vis studies. During all these studies there was minimal shifting of all the proton resonances, including the CH_3_ methyl groups. This suggests no hydrogen bonding between the anion and the CH_3_ groups. As such, we employed UV-Vis spectroscopic titrations. UV-vis titrations of 1–4·OTf^−^ were conducted with tetra-*n*-butylammonium bromide (TBABr) at 20 °C in a THF/DMSO/H_2_O (90/9.9/0.1) mixture to ensure that all compounds were soluble and that there was a constant amount of water present (additional details in the ESI†). Upon addition of TBABr to the solution of 1·OTf the absorbance band around 385 nm underwent a hypochromic shift. In contrast, the addition of TBABr to 2·OTf and 3·OTf led to hyperchromic shifts of absorbances around 365 nm and 375 nm. 4·OTf on the other hand had absorption bands around 375 nm and 350 nm grow and decrease respectively when increasing the amount of TBABr, creating an isosbestic point around 363 nm. These spectroscopic changes (see ESI†) were used to determine association constants (*K*_a_) by fitting the change in the absorbance to a 1 : 1 binding model using Bindfit.^36,37^ The *K*_a_ values measured followed the trend of 1·OTf > 4·OTf > 2·OTf > 3·OTf (Table 3) and corelate with the interaction energy trends obtained from the theoretical investigations. 1·OTf had the strongest binding (26 000 M^−1^). When comparing the values for 2·OTf and 3·OTf there is a slight difference in receptor performance. 2·OTf has a slightly larger association constant than 3·OTf (15 000 M^−1^*vs.* 12 000 M^−1^) indicating that C–H HBeXB is improving binding. As alluded to in the theoretical evaluations (as well as previous HBeXB papers), this is likely due to a combination of both preorganization as well as halogen bond enhancement. For comparison, 4·OTf (with the amine hydrogen bond donor) exhibited stronger binding (18 000 M^−1^) than 2·OTf and 3·OTf.

**Table 3:** Association constants with TBABr^a^

Receptor	1-OTf	2-OTf	3-OTf	4-OTf
Average (M^−1^)	26 000	15 000	12 000	18 000

a Association constants for binding of TBABr to all receptors in 90% THF/9.9% DMSO/0.1% deionized H_2_O at 293 K. Error is less than ±10%. The values presented are the average of three titrations.

## Conclusion

In conclusion, we have synthesized a series of halogen bond anion receptors to systematically evaluate C–H HBeXB. Collectively, the computational, solid-state, and solution phase evaluations suggest that C–H hydrogen bonds to iodine atoms can improve halogen bond receptor performance. The location of the charge is important—having the charge located near the binding pocket can produce C–H HBeXB receptors that outperform traditional HB donors where the charge is further away. In contrast, weaker C–H donors may be the limit to this polarization enhanced noncovalent interaction. The data also provide rare evidence of a hydrogen bond between C–H donors and iodine acceptors and suggest that preorganization may be a dominate factor, while the augmentation of the halogen bond seems to be subtle based on the solid-state and theoretical data. The identification of C–H HBeXB has broad implications for supramolecular designs as traditional hydrogen bonds are often used to preorganize molecular structure. These common N and O hydrogen bond donors are pH sensitive which can, in some circumstances, limit their utility. In contrast, non-traditional C–H donors offer pH insensitivity and, when coupled with the pH insensitive halogen bond, highlights one functional potential for this unique supramolecular design. Future studies are intended to target solvent effects and preorganization studies to better understand the influence of C–H hydrogen bonds to iodine atoms.

## Data availability

See ESI.†

## Author contributions

DAD and OBB conceptualized the project. DAD, JS, and MRB conducted synthesis and characterization. DAD conducted computational and solid-state studies. DAD and JS conducted the solution studies. DAD wrote the paper. OBB supervised the investigation and provided editorial assistance during manuscript preparation. All authors examined the data, results, and conclusions presented here.

## Conflicts of interest

There are no conflicts to declare.

## Supplementary Material

SC-013-D2SC03792K-s001

SC-013-D2SC03792K-s002
