# Supplementary material for: Pushing the limits of the hydrogen bond enhanced halogen bond—the case of the C–H hydrogen bond
Source: Chem Sci. 2022 Aug 31;13(37):11156–62. doi: 10.1039/d2sc03792k (PMC9516949; doi:10.1039/d2sc03792k)
Supplement: SC-013-D2SC03792K-s001 [file SC-013-D2SC03792K-s001.pdf]

This journal is © The Royal Society of Chemistry 2022

# Evaluating the Limits of the Hydrogen Bond Enhanced Halogen Bond —The Case of the C–H Hydrogen Bond

Daniel A. Decato, Jiyu Sun, Madeleine R. Boller, Orion B. Berryman*
* Department of Chemistry and Biochemistry, University of Montana, 32 Campus Drive, Missoula, Montana, United States, 59812

## Table of Contents


| Content | Page |
|---|---|
| General Methods | S2 |
| Single Crystal X-ray Diffraction Methods, Refinement, and Tables | S2-S5 |
| General Synthetic Scheme | S6 |
| Synthetic Procedures and Characterization | S6-S39 |
| Computational Details, Results, and Atomic Coordinates | S39-S61 |
| Titration Details and Data | S61-S64 |
| References | S64 |

**General methods**
All reagents were obtained from commercial sources and were used without further purification unless otherwise noted. Thin layer chromatography (TLC)was performed using normal-phase silica gel glass-backed plates (0.25 mm, F-254, SiliCycle) and observed under UV light. Flash column chromatography was performed using normal-phase silica gel (230–400 mesh, SiliaFlash®P60, SiliCycle. All compounds were dried in vacuo at room temperature as needed. High-resolution mass spectrometry was carried out using an Agilent 6520 Accurate-Mass Q-TOF LC/MS. All UV-Vis spectra were collected on a Cary 60 spectrometer at 298 K. Nuclear magnetic resonance (NMR) spectra were obtained with a VNMRS Varian 500 MHz or a Bruker Avance 400 MHz or an Agilent 400 MHz spectrometer. Chemical shifts are reported in parts per million (ppm) from high to low frequency using the residual solvent peak as the internal reference and are reported at 25°C unless noted. For the $^{19}F$ NMR spectra monofluorobenzene (-113.15ppm) was used as an internal standard. Signal splitting patterns are indicated as s, singlet; d, doublet; t, triplet; m, multiplet, b, broad. Coupling constants (J) are given in Hz.

**Single Crystal X-ray Diffraction Methods and Refinement**

X-ray diffraction data for all structures were collected at 100 K on a Bruker D8 Venture using MoKα-radiation (λ=0.71073 Å). Data have been corrected for absorption using SADABS[1]area detector absorption correction program. Using Olex2[2], the structure was solved with the SHELXT[3]structure solution program using Direct Methods and refined with the SHELXL[4]refinement package using least squares minimization. All non-hydrogen atoms were refined with anisotropic thermal parameters. Hydrogen atoms attached to heteroatoms were found from the residual density maps, placed, and refined with isotropic thermal parameters. All other hydrogen atoms in the investigated structure were located from difference Fourier maps but finally their positions were placed in geometrically calculated positions, and refined using a riding model. Isotropic thermal parameters of the placed hydrogen atoms were fixed to 1.2 times the U value of the atoms they are linked to. Calculations and refinement of structures were carried out using APEX3[5], SHELXTL[6], and Olex2 software. Crystallographic data for all structures are presented in a table below. Additionally, individual refinement details and crystal growth conditions are presented below.

**Crystal growth conditions and additional refinement details**

---

**1•OTf⁻**
Diffraction quality yellow rods were grown from slow evaporation of a methanol solution of 1•OTf⁻.

---

**2•OTf⁻**
Diffraction quality colorless needles were grown by vapor diffusion of diethyl ether into a nitromethane solution of **2•OTf⁻.**

---

**3•OTf⁻**

Diffraction quality colorless needles were grown by vapor diffusion of diethyl ether into a nitromethane solution of **3•OTf**$^{-}$**.**

**4•OTf**$^{-}$

Diffraction quality colorless rods were grown by vapor diffusion of ether into a nitromethane solution of **4•OTf**$^{-}$**.**

After initial solution and refinement of the data it was apparent that the triflate anion was disordered. The anion has been modeled over two positions using a PART instruction and tied to an individual free variable. Refinement of the free variable showed an approximate 70:30 disorder. The disorder model incorporated bond length similarity restraints (SADI). One of the amine hydrogen atoms after refinement elongated necessitating the use of bond length restraints (DFIX 0.87 0.02).

**1•I**$^{-}$

Diffraction quality yellow prisms were grown by vapor diffusion of diethyl ether into a methanol solution of **1•OTf** and tetra-*n*-hexylammonium iodide.

**2•I**$^{-}$

Diffraction quality yellow needles were grown by vapor diffusion of diethyl ether into a methanol solution of solution of **2•OTf** and tetra-*n*-hexylammonium iodide.

**3•I**$^{-}$

Diffraction quality yellow rods were grown by vapor diffusion of diethyl ether into a methanol solution of **3•OTf** and tetra-*n*-hexylammonium iodide.

**4•I**$^{-}$

Diffraction quality yellow rods were grown by vapor diffusion of diethyl ether into a methanol solution of **4•OTf** and tetra-*n*-hexylammonium iodide.

The amine hydrogen atoms necessitated bond distance restraints (DFIX 0.87 (0.02)) due to unreasonable bond length shortening upon refinement.

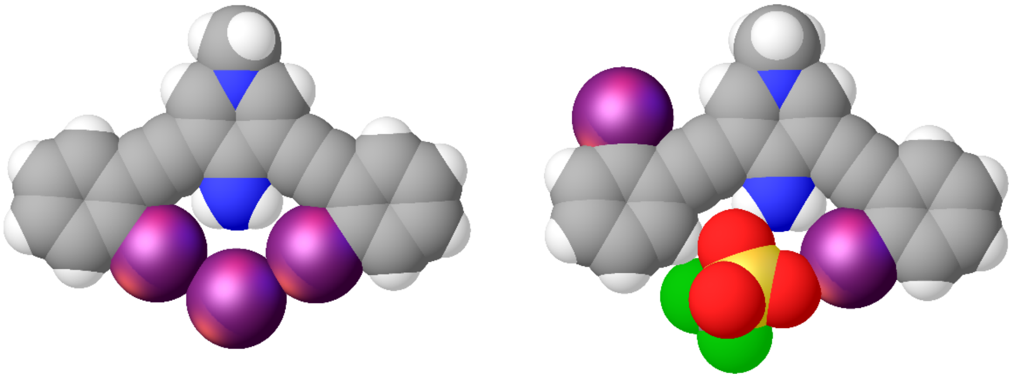

**4•I**$^{-}$ **4•OTf**$^{-}$



Figure 1S Asymmetric units of both **4•I**$^{-}$and **4•OTf**$^{-}$ X-ray crystal structures. Spheres drawn using the default vdW radii within Olex2.

Table 1S: Crystallographic Data

| Identification code | UMT_OB149 | UMT_OB206 | UMT_OB191 | UMT_OB216 |
|---|---|---|---|---|
| Manu reference | 1•OTf⁻ | 2•OTf⁻ | 3•OTf⁻ | 4•OTf⁻ |
| CCDC number | 2075099 | 2075104 | 2075103 | 2075102 |
| Empirical formula | $C_{23}H_{14}F_3I_2NO_3S$ | $C_{24}H_{16}F_3I_2NO_3S$ | $C_{23}H_{14}F_3I_2NO_3S$ | $C_{23}H_{15}F_3I_2N_2O_3S$ |
| Formula weight | 695.21 | 709.24 | 695.21 | 710.23 |
| Crystal system | triclinic | triclinic | triclinic | tetragonal |
| Space group | *P*-1 | *P*-1 | *P*-1 | $P\text{-}42_1c$ |
| a/Å | 8.5783(4) | 7.2077(5) | 7.1362(3) | 22.544(3) |
| b/Å | 11.1636(6) | 12.3954(8) | 12.3559(6) | 22.544(3) |
| c/Å | 13.3063(7) | 14.0967(9) | 14.2656(7) | 9.5927(11) |
| α/° | 74.307(2) | 98.565(2) | 98.460(2) | 90 |
| β/° | 71.324(2) | 101.823(2) | 103.067(2) | 90 |
| γ/° | 79.269(2) | 91.413(2) | 94.936(2) | 90 |
| Volume/Å$^3$ | 1155.23(10) | 1216.98(14) | 1202.63(10) | 4875.1(13) |
| Z | 2 | 2 | 2 | 8 |
| $\rho_{calc}$ g/cm$^3$ | 1.999 | 1.935 | 1.920 | 1.935 |
| Crystal size/mm$^3$ | 0.59 × 0.06 × 0.04 | 0.24 × 0.03 × 0.02 | 0.20 × 0.05 × 0.03 | 0.20 × 0.15 × 0.02 |
| 2Θ range for data collection/° | 5.042 to 57.518 | 5.784 to 55.75 | 5.906 to 56.556 | 5.11 to 52.826 |
| Reflections collected | 36937 | 64454 | 86479 | 142896 |
| Independent reflections | 5990 [$R_{int}$ = 0.0493, $R_{sigma}$ = 0.0345] | 5808 [$R_{int}$ = 0.0516, $R_{sigma}$ = 0.0271] | 5965 [$R_{int}$ = 0.0345, $R_{sigma}$ = 0.0145] | 4993 [$R_{int}$ = 0.0778, $R_{sigma}$ = 0.0257] |
| Data/restraints/parameters | 5990/0/299 | 5808/0/297 | 5965/0/299 | 4993/13/371 |
| Goodness-of-fit on $F^2$ | 1.079 | 1.159 | 1.166 | 1.098 |
| Final R indexes [I>=2σ (I)] | $R_1$ = 0.0276, $wR_2$ = 0.0474 | $R_1$ = 0.0315, $wR_2$ = 0.0650 | $R_1$ = 0.0275, $wR_2$ = 0.0658 | $R_1$ = 0.0248, $wR_2$ = 0.0467 |
| Final R indexes [all data] | $R_1$ = 0.0451, $wR_2$ = 0.0517 | $R_1$ = 0.0441, $wR_2$ = 0.0685 | $R_1$ = 0.0330, $wR_2$ = 0.0678 | $R_1$ = 0.0358, $wR_2$ = 0.0506 |
| Largest diff. peak/hole / e Å$^{-3}$ | 1.00/-0.57 | 1.48/-0.61 | 2.15/-1.41 | 0.49/-0.35 |

| | | | | |
|---|---|---|---|---|
| Identification code | UMT_OB154 | UMT_OB193 | UMT_OB175 | UMT_OB214a |
| Manu reference | 1•I⁻ | 2•I⁻ | 3•I⁻ | 4•I⁻ |
| CCDC number | 2075101 | 2075098 | 2075105 | 2075100 |
| Empirical formula | $C_{22}H_{14}I_3N$ | $C_{23}H_{16}I_3N$ | $C_{22}H_{14}I_3N$ | $C_{22}H_{15}I_3N_2$ |
| Formula weight | 673.04 | 687.07 | 673.04 | 688.06 |
| Crystal system | triclinic | orthorhombic | orthorhombic | orthorhombic |
| Space group | *P*-1 | *Pbcn* | *Pbcn* | *Pbcn* |
| a/Å | 8.0838(2) | 21.1343(8) | 21.0383(12) | 21.0883(17) |
| b/Å | 11.8898(4) | 13.7339(7) | 13.7820(8) | 13.7730(11) |
| c/Å | 12.2871(4) | 7.3280(3) | 7.3031(4) | 7.3022(6) |
| α/° | 64.3550(10) | 90 | 90 | 90 |
| β/° | 77.9010(10) | 90 | 90 | 90 |
| γ/° | 77.3900(10) | 90 | 90 | 90 |
| Volume/$Å^3$ | 1030.02(6) | 2127.00(16) | 2117.5(2) | 2120.9(3) |
| Z | 2 | 4 | 4 | 4 |
| $\rho_{calc}g/cm^3$ | 2.170 | 2.146 | 2.111 | 2.155 |
| Crystal size/$mm^3$ | 0.19 × 0.1 × 0.08 | 0.24 × 0.01 × 0.01 | 0.45 × 0.02 × 0.02 | 0.61 × 0.02 × 0.02 |
| 2Θ range for data collection/° | 5.208 to 56.716 | 5.932 to 50.154 | 5.912 to 59.212 | 5.916 to 61.014 |
| Reflections collected | 43567 | 28749 | 44308 | 29568 |
| Independent reflections | 5147 [$R_{int}$ = 0.0294, $R_{sigma}$ = 0.0149] | 1892 [$R_{int}$ = 0.1025, $R_{sigma}$ = 0.0325] | 2984 [$R_{int}$ = 0.0595, $R_{sigma}$ = 0.0283] | 3244 [$R_{int}$ = 0.0633, $R_{sigma}$ = 0.0385] |
| Data/restraints/parameters | 5147/0/236 | 1892/0/127 | 2984/0/121 | 3244/1/130 |
| Goodness-of-fit on $F^2$ | 1.097 | 1.053 | 1.058 | 1.063 |
| Final R indexes [I>=2σ (I)] | $R_1$ = 0.0171, $wR_2$ = 0.0352 | $R_1$ = 0.0251, $wR_2$ = 0.0345 | $R_1$ = 0.0265, $wR_2$ = 0.0446 | $R_1$ = 0.0362, $wR_2$ = 0.0671 |
| Final R indexes [all data] | $R_1$ = 0.0218, $wR_2$ = 0.0362 | $R_1$ = 0.0453, $wR_2$ = 0.0382 | $R_1$ = 0.0444, $wR_2$ = 0.0484 | $R_1$ = 0.0636, $wR_2$ = 0.0744 |
| Largest diff. peak/hole / e $Å^{-3}$ | 0.76/-0.37 | 0.53/-0.53 | 0.76/-0.50 | 1.33/-1.21 |

**General Synthetic Scheme**

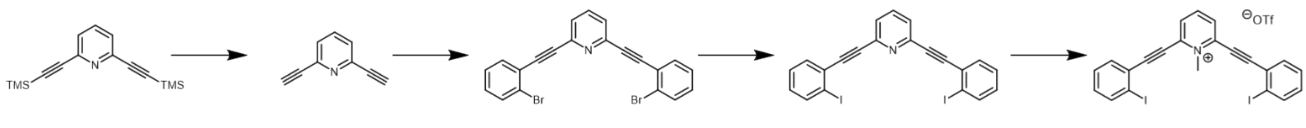

1CorePro 1CoreDePro 1Br 1Neu 1

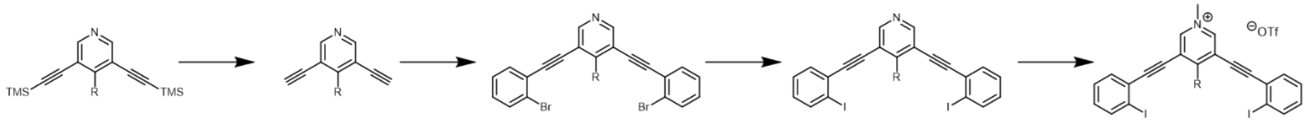

| 2CorePro R = Me | 2CoreDePro R = Me | 2Br R = Me | 2Neu R = Me | 2 R = Me |
|---|---|---|---|---|
| 3CorePro R = H | 3CoreDePro R = H | 3Br R = H | 3Neu R = H | 3 R = H |
| 4CorePro R = $NH_2$ | 4CoreDePro R = $NH_2$ | 4Br R = $NH_2$ | 4Neu R = $NH_2$ | 4 R = $NH_2$ |

**Synthetic Procedures and Characterization**

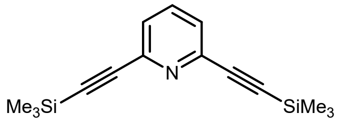


**1CorePro** Synthesis conducted according to previously reported procedure.[7]

---

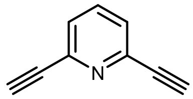


**1CoreDePro** Synthesis conducted according to previously reported procedure.[8]

---

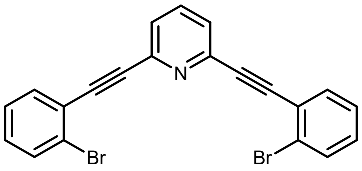


**Procedure:** A flame dried Schlenk flask was charged with 2,6-diethynylpyridine (**1CoreDePro**) (0.2 g, 1.57 mmol), Bis(triphenylphosphine)palladium (II) dichloride (0.044 g, 0.06 mmol) and Copper (I) iodide (0.011 g, 0.06 mmol) and then sealed with a rubber septum. The Schlenk flask was then evacuated and backfilled with dry nitrogen gas three times. To a flame dried 50 ml round bottom flask was added tetrahydrofuran and triethylamine. The round bottom was sealed with a rubber septum and then sparged with dry nitrogen gas for 20 minutes, after which 2-bromo-iodobenzene (1.11 g, 3.93 mmol) was added and sparging resumed for 2 minutes. The 2-bromo-iodobenzene solution was then canula transferred to the Schlenk flask and stirred at room temperature overnight. The crude reaction mixture was concentrated and purified by silica gel column chromatography (5% ethyl acetate in hexanes) to afford **1Br** (0.448 g, 1.02 mmol, 65 % yield) as a white solid.

**$^1$H NMR (500 MHz, $CDCl_3$)** δ 7.71 (t, $J$ = 7.4 Hz, 1H), 7.66 – 7.61 (m, 4H) (overlap of two doublets), 7.57 (d, $J$ = 7.8 Hz, 2H), 7.32 (td, $J$ = 7.6, 1.2 Hz, 2H), 7.23 (td, $J$ = 7.8, 1.7 Hz, 2H).

**$^{13}$C NMR (126 MHz, CDCl$_3$)** δ 143.76, 136.59, 134.04, 132.65, 130.38, 127.26, 127.09, 126.07, 124.50, 92.47, 88.24.

**HRMS (ESI pos) m/z for $C_{21}H_{12}Br_2N^+$ $[M+H]^+$ :** calculated:437.9311; found:437.9354

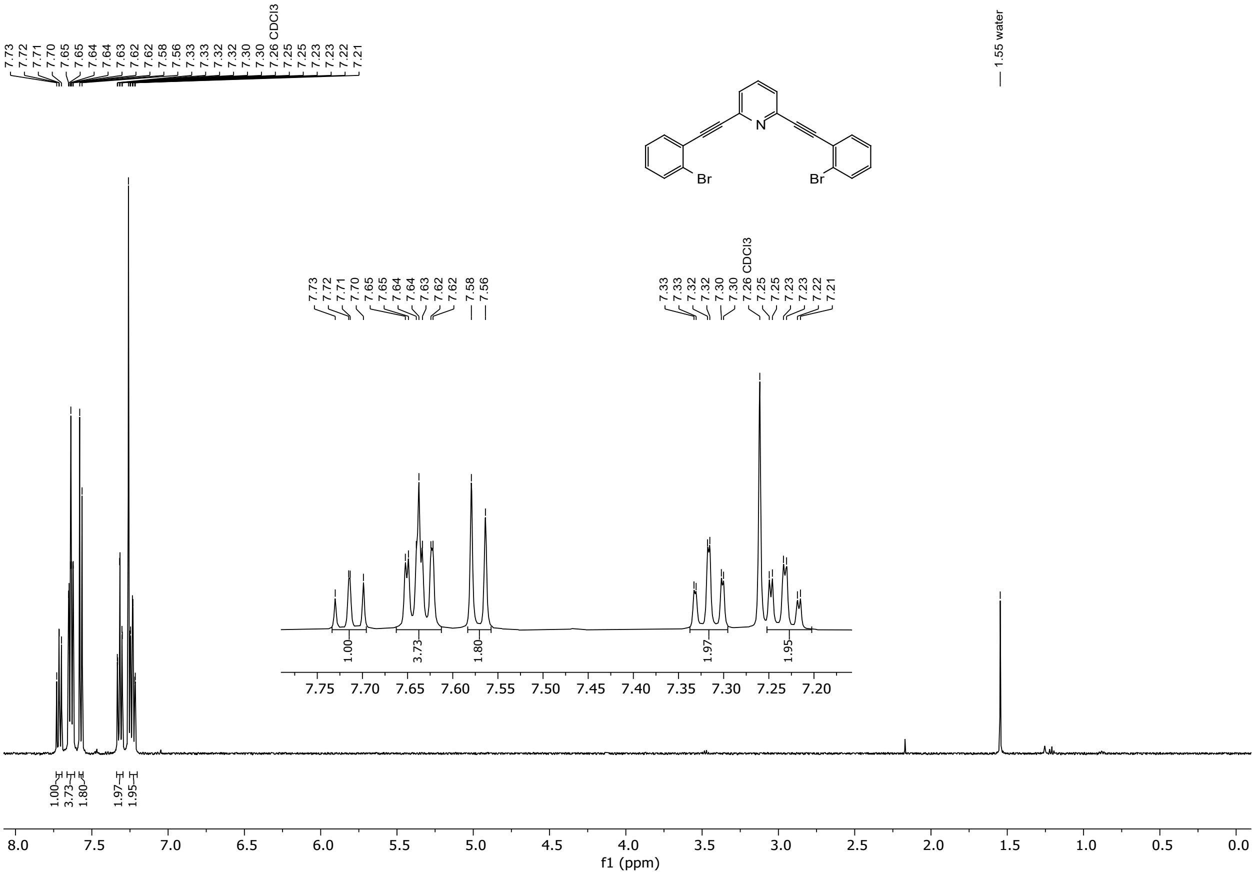


Figure 2S $^1$H NMR spectrum of **1Br** (500 MHz, CDCl3)

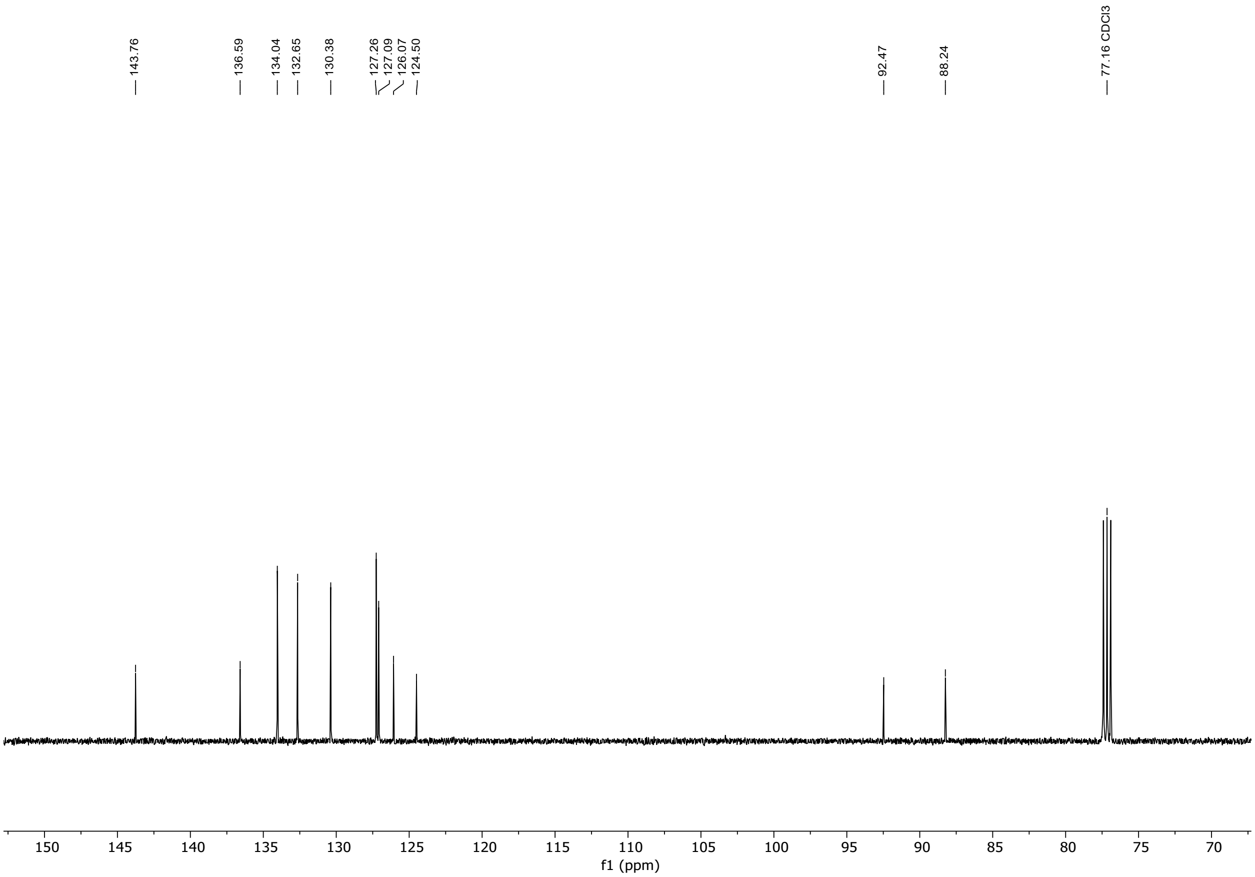


Figure 3S $^{13}C$ NMR spectrum of **1Br** (126 MHz, $CDCl_3$)

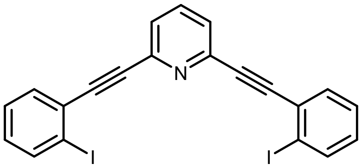


**Procedure:** An oven dried round bottom (50 mL) was charged with **1Br** (0.10 g, 0.228 mmol) was subsequently dissolved in 20 mL of dry tetrahydrofuran and cooled to -67℃ (dry ice and acetone bath). N-butyllithium (1.6 M in hexanes, 0.36 mL, 0.57 mmol) was added dropwise to the colorless solution producing a yellow solution that darkened overtime. The mixture was stirred for 30 min at -67℃. Iodine (0.29 g, 1.14 mmol) in 5 mL of tetrahydrofuran was cooled to -67˚C then added dropwise. The resulting blood red solution was allowed to gradually warm to room temperature and stirred overnight. The crude reaction mixture was washed with a saturated aqueous sodium thiosulfate solution and extracted with diethyl ether. The organic layers were combined and dried with magnesium sulfate. The crude product was loaded onto C18 silica gel and subsequently purified via prep-HPLC to afford **1Neu** (0.1 g, 0.188 mmol, 82 % yield) as a beige solid.

**$^{1}$H NMR (500 MHz, CDCl$_3$)** δ 7.89 (d, *J* = 8.1 Hz, 1H), 7.73 (t, *J* = 7.2 Hz, 1H), 7.62 (dd, *J* = 7.8, 1.8 Hz, 2H), 7.61 (d, *J* = 7.8 Hz, 2H), 7.36 (t, *J* = 7.6 Hz, 1H), 7.07 (td, *J* = 7.8, 1.7 Hz, 2H).
**$^{13}$C NMR (126 MHz, CDCl$_3$)** δ 143.80, 138.95, 136.59, 133.39, 130.36, 128.87, 128.07, 127.06, 101.30, 91.74, 91.62.
**HRMS (ESI pos) m/z for $C_{21}H_{12}I_2N^+$ [M+H]$^+$ :** calculated: 531.9054 found:531.9112

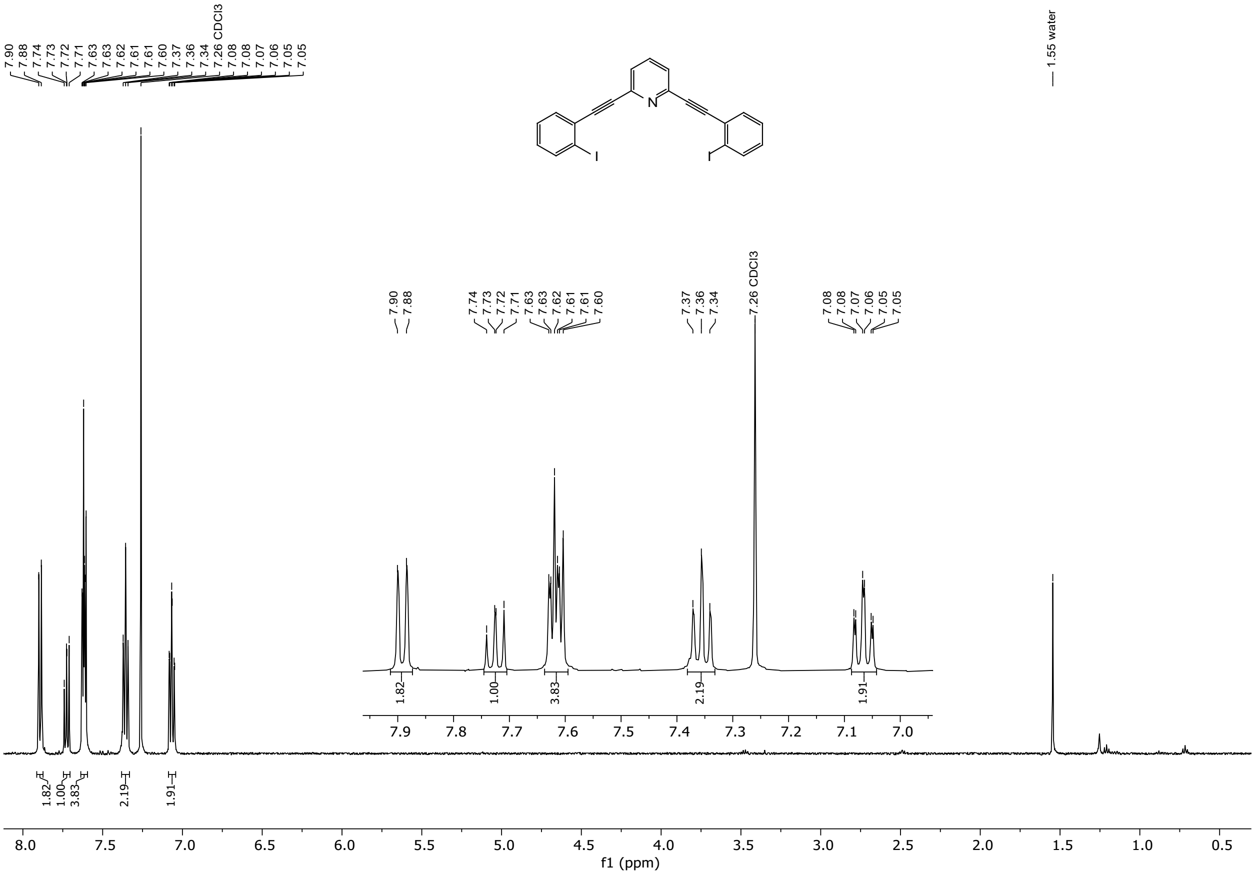

Figure 4S $^{1}$H NMR spectrum of **1Neu** (500 MHz, CDCl3)

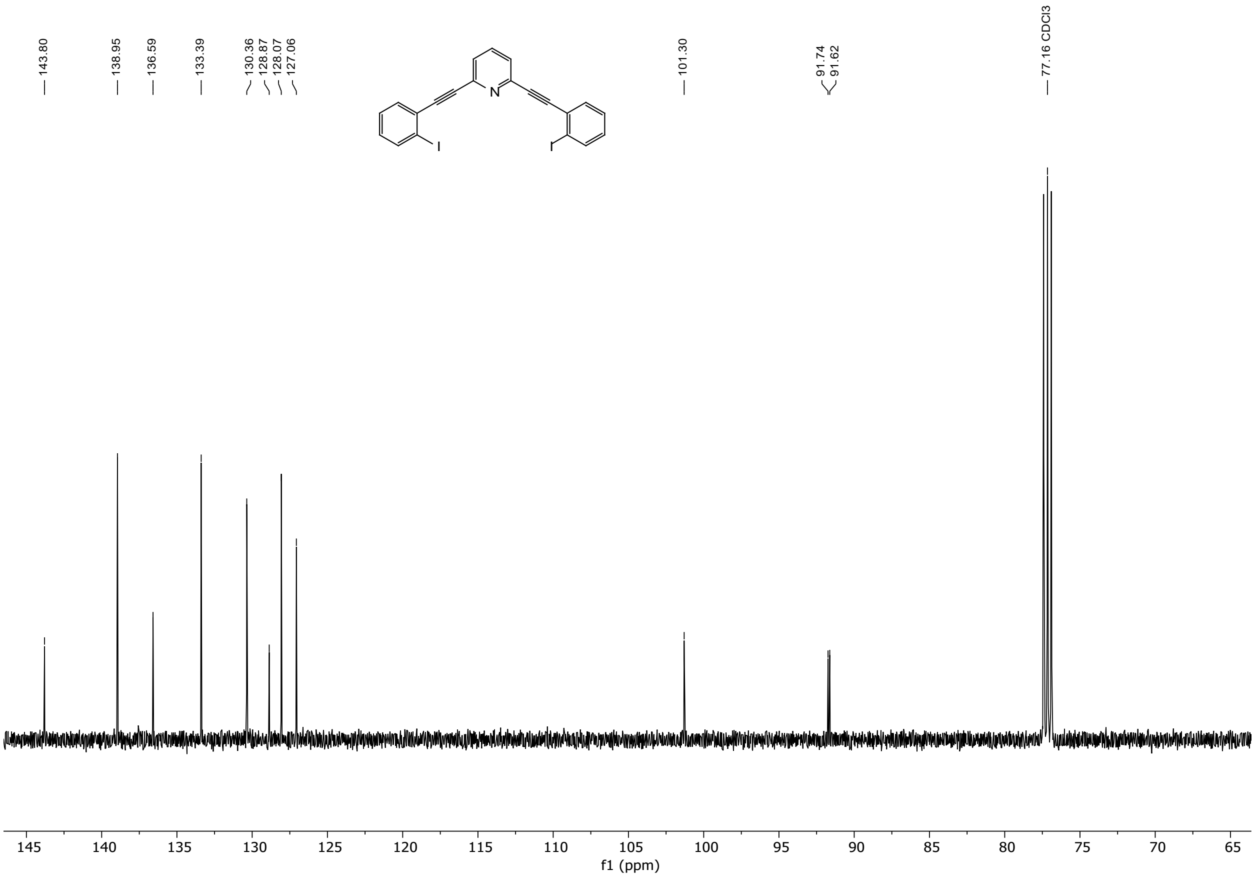

Figure 5S $^{13}$C NMR spectrum of **1Neu** (126 MHz, $CDCl_3$)

---

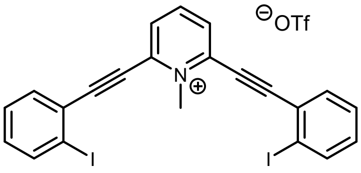


**Procedure:** To a flame dried scintillation vial, **1Neu** (0.1 g, 0.188 mmol) was dissolved in 12ml of dry dichloromethane. Methyl trifluoromethanesulfonate(0.046 g, 0.28 mmol) was added dropwise to the solution, after which the vial was capped then allowed to stir at room temperature for 1-2 days. A light yellow precipitate formed, addition of diethyl ether facilitated further precipitation allowing **1** to be isolated by filtration. The solid was then washed with diethyl ether to afford **1** (0.12 g, 0.172 mmol, 91 % yield) as a light yellow solid.
**$^{1}$H NMR (500 MHz, DMSO-d6)** δ 8.63 (t, $J$ = 8.0 Hz, 1H), 8.41 (d, $J$ = 8.0 Hz, 2H), 8.10 (dd, $J$ = 8.0, 1.1 Hz, 2H), 7.93 (dd, $J$ = 7.7, 1.6 Hz, 2H), 7.63 (td, $J$ = 7.6, 1.1 Hz, 2H), 7.39 (td, $J$ = 7.8, 1.7 Hz, 2H), 4.71 (s, 3H).
**$^{13}$C NMR (126 MHz, DMSO-d6)** δ 144.12, 139.22, 138.28, 134.83, 133.24, 131.28, 128.78, 125.52, 106.53, 101.90, 83.37, 46.62. The $^{13}$C resonance of the triflate anion (quartet with relative intensities of 1:3:3:1) was not observed)
**$^{19}$F NMR (470 MHz, $CD_3CN$)** δ -77.55

**HRMS (ESI pos) m/z for $C_{22}H_{14}I_2N^+$ [M]$^+$ :** calculated: 545.9210 found: 545.9232

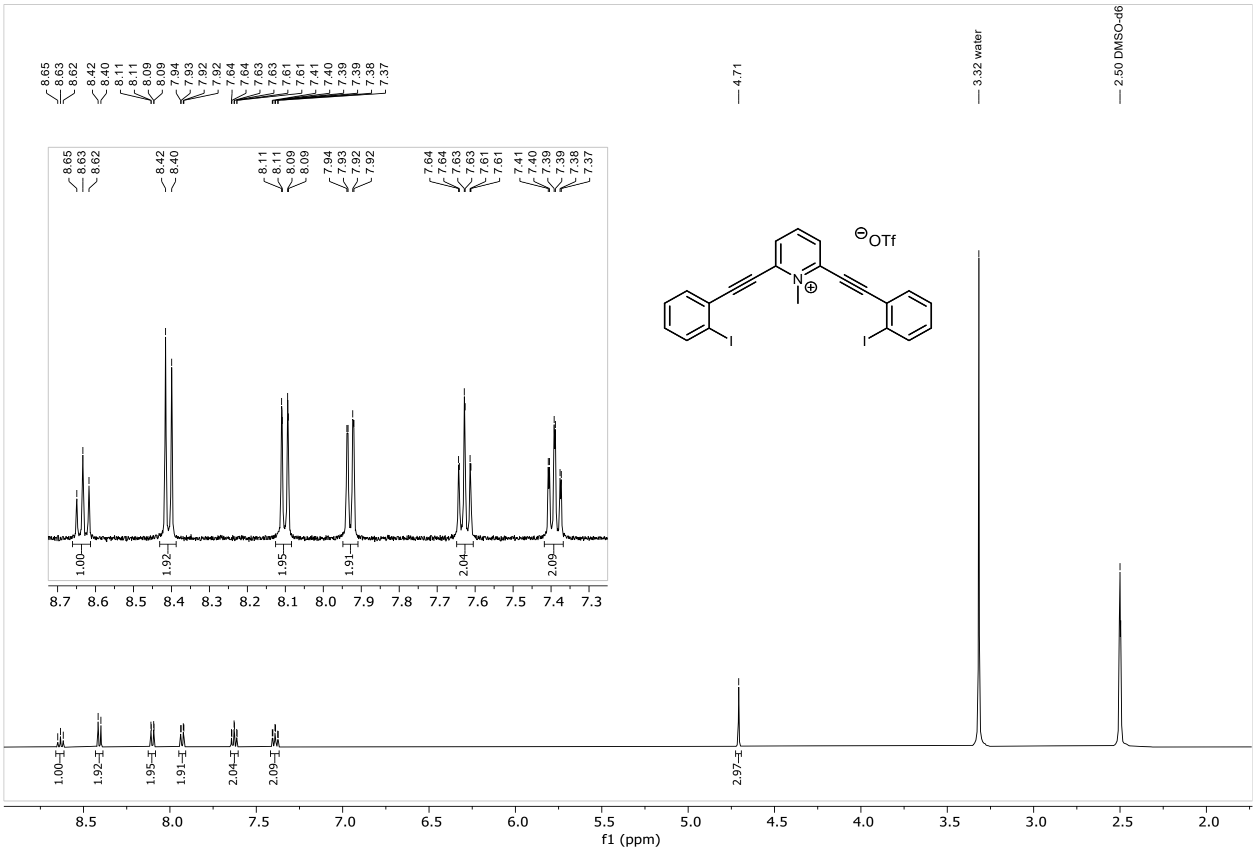

Figure 6S $^1$H NMR spectrum of **1** (500 MHz, DMSO)

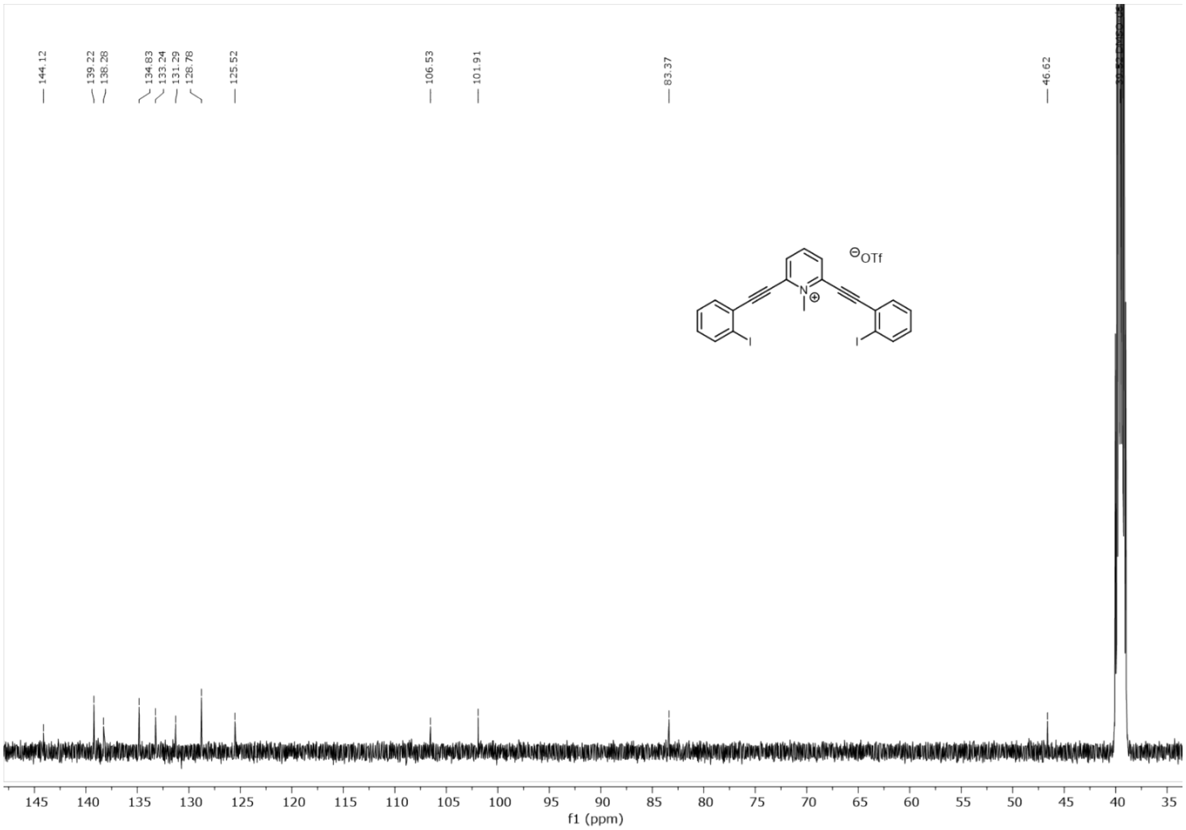

Figure 7S $^{13}C$ NMR spectrum of **1** (126 MHz, DMSO)

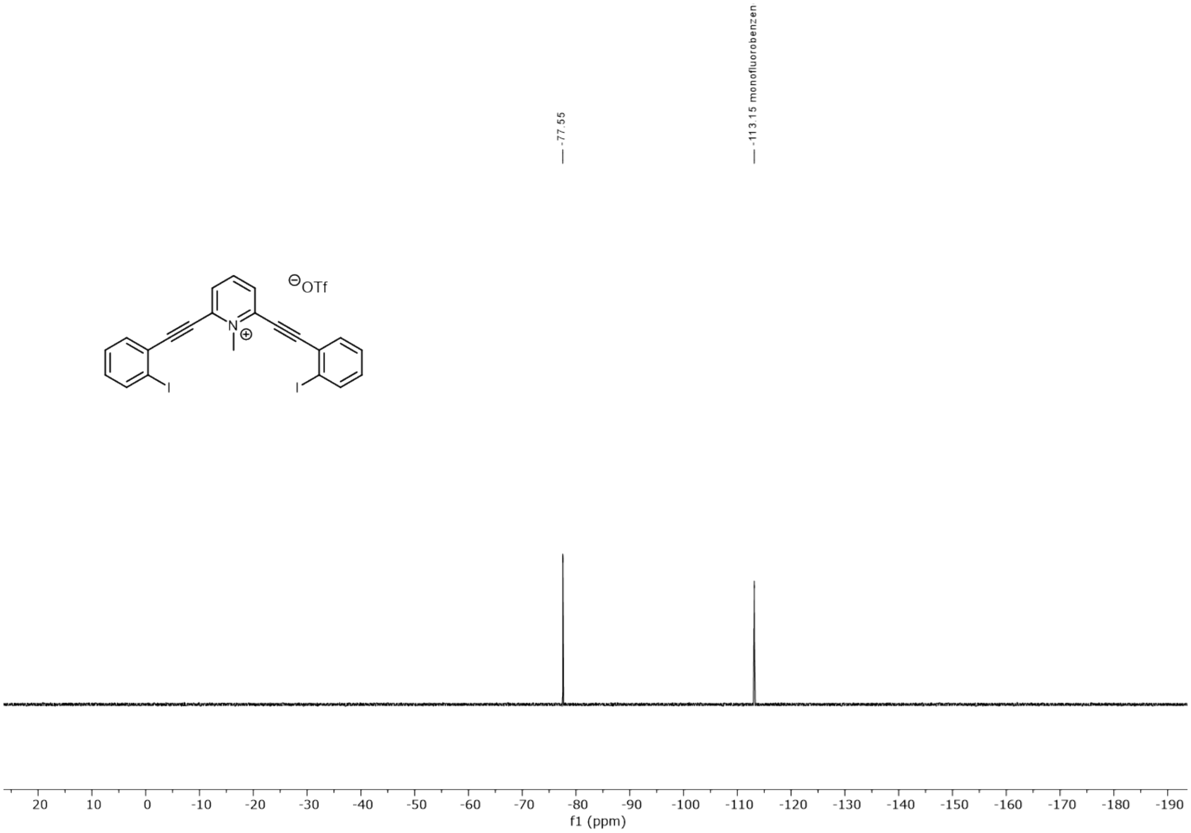


Figure 8S $^{19}$F NMR spectrum of **1** (470 MHz, $CD_3CN$). $C_6H_5F$ (monofluorobenzene) internal reference.

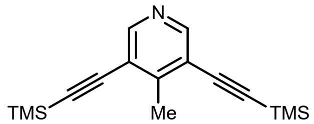


**2CorePro** synthesis and spectroscopic data in accordance with previously reported material. CrystEngComm (2017), 19, (23), 3094-3097.

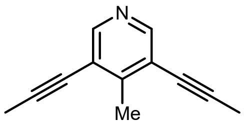


**2CoreDePro** synthesis and spectroscopic data in accordance with previously reported material. CrystEngComm (2017), 19, (23), 3094-3097.

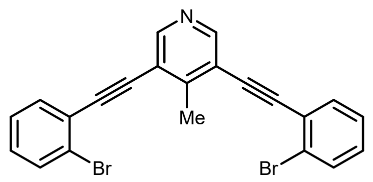


A flame dried Schlenk flask was charged with 3,5-diethynyl-4-methyl-pyridine (**2CoreDePro**) (0.3 g, 2.12 mmol), Bis(triphenylphosphine)palladium (II) dichloride (0.06 g, 0.085 mmol) and Copper (I) iodide (0.016 g, 0.085 mmol) and then sealed with a rubber septum. The Schlenk flask was then evacuated and backfilled with dry nitrogen gas three times. To a flame dried 50 ml round bottom flask was added tetrahydrofuran and triethylamine. The round bottom was sealed with a rubber septum and then sparged with dry nitrogen gas for 20 minutes, after which 2-bromo-iodobenzene (1.5 g, 5.31mmol) was added and sparging resumed for 2 minutes. The 2-bromo-iodobenzene solution was then canula transferred to the Schlenk flask and stirred at room temperature overnight. The crude reaction mixture was concentrated and purified by silica gel column chromatography (10% ethyl acetate in hexanes) to afford **2Br** (0.787 g, 1.74 mmol, 82 % yield) as a beige solid.

**$^{1}$H NMR (500 MHz, CDCl$_3$)** δ 8.67 (s, 2H), 7.65 (dd, $J$ = 8.1, 1.2 Hz, 2H), 7.61 (dd, $J$ = 7.7, 1.7 Hz, 2H), 7.34 (td, $J$ = 7.6, 1.2 Hz, 2H), 7.24 (td, $J$ = 7.8, 1.7 Hz, 2H), 2.81 (s, 3H).

**$^{13}$C NMR (126 MHz, CDCl$_3$)** δ 151.50, 151.04, 133.60, 132.74, 130.13, 127.31, 125.75, 124.97, 120.41, 94.98, 89.17, 19.46.

**HRMS (ESI pos) m/z for $C_{22}H_{14}Br_2N^+$ $[M+H]^+$ :** calculated: 451.9467 found: 451.9479

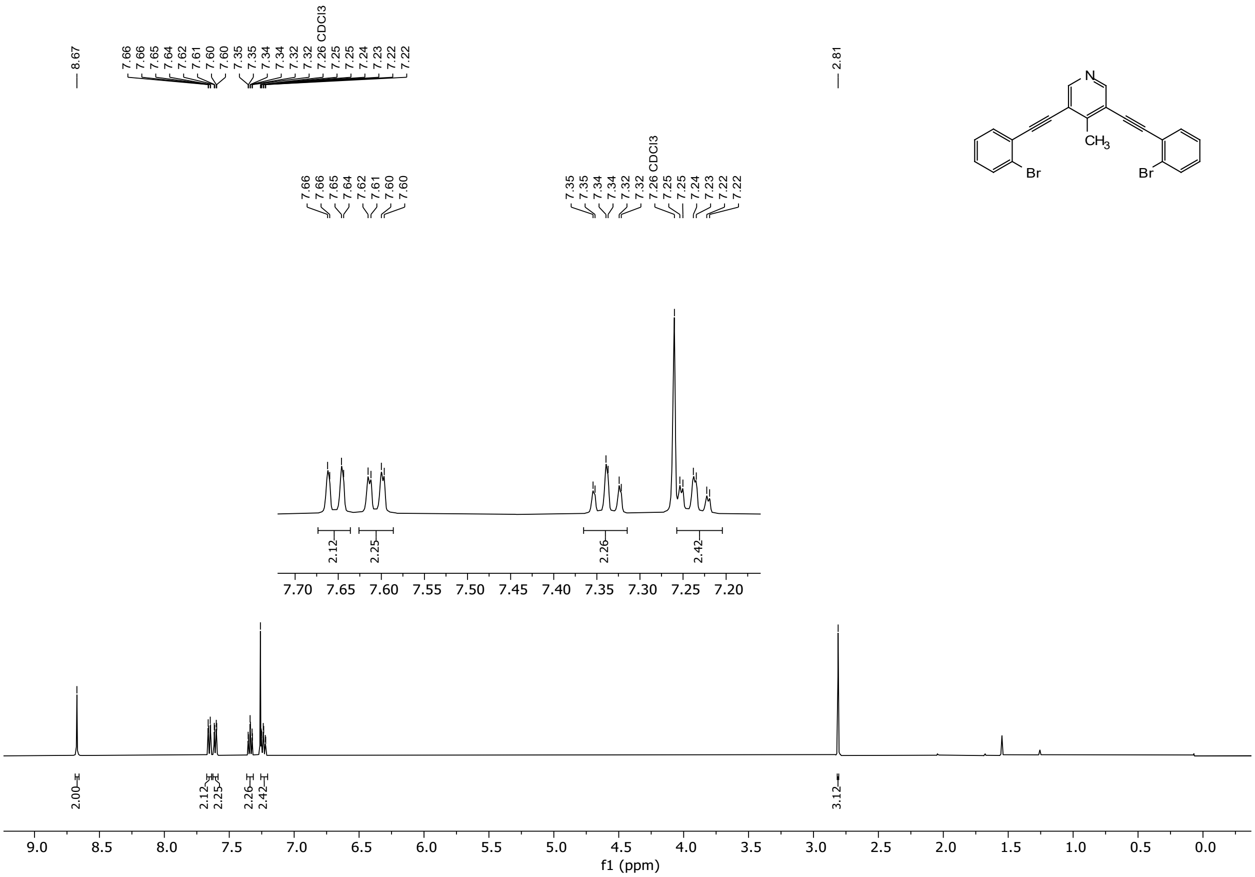

Figure 9S $^{1}H$ NMR spectrum of **2Br** (500 MHz, $CDCl_3$)

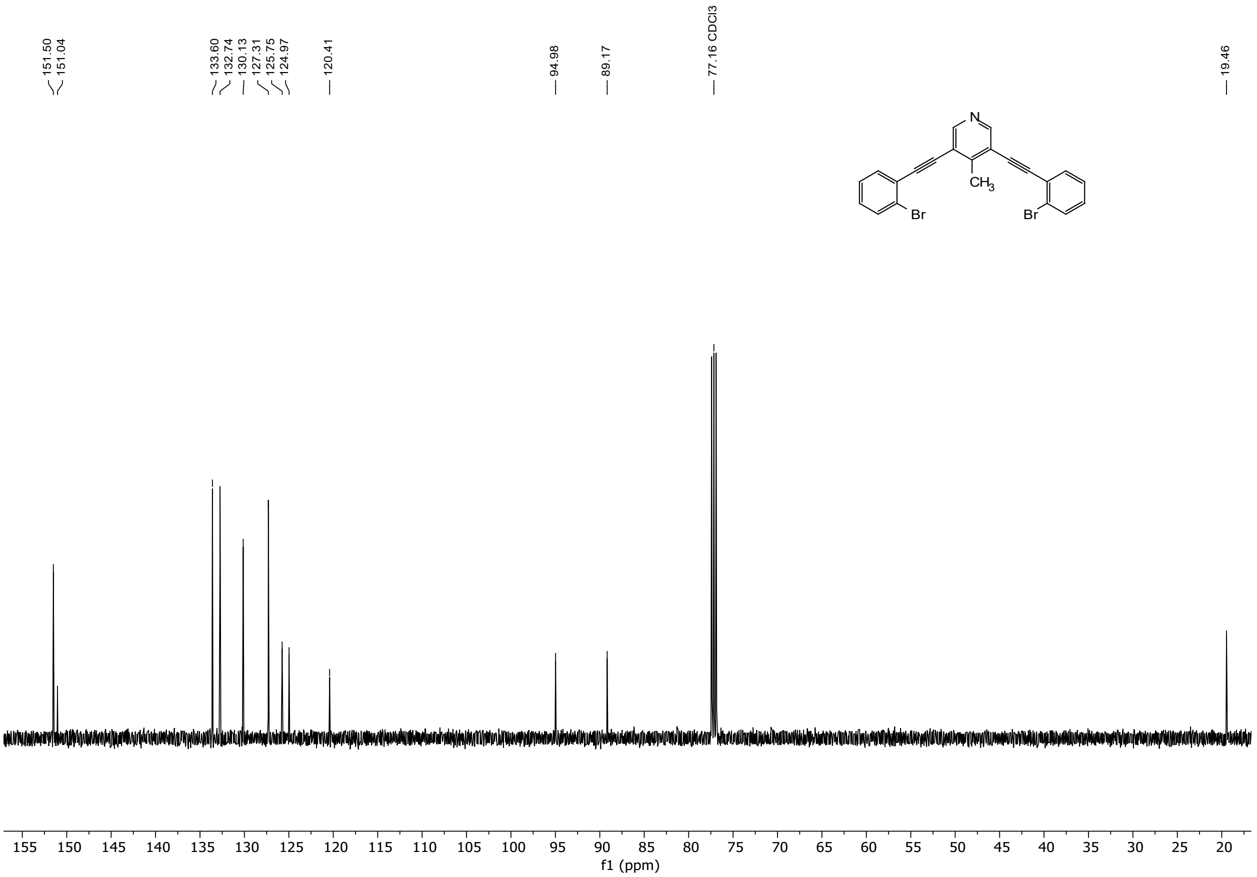

Figure 10S $^{13}$C NMR spectrum of **2Br** (126 MHz, $CDCl_3$)

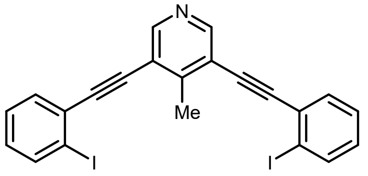


An oven dried round bottom (50 mL) was charged with **2Br** (0.2 g, 0.44 mmol) was subsequently dissolved in 20 mL of dry tetrahydrofuran and cooled to -67℃ (dry ice and acetone bath). N-butyllithium (1.6 M in hexanes, 0.7 mL, 1.12 mmol) was added dropwise producing a deep beet red colored solution. The mixture was stirred for 30 min at -67℃. Iodine (0.56 g, 2.22 mmol) in 5 mL of tetrahydrofuran was cooled to -67˚C then added dropwise. The resulting dark red solution was allowed to gradually warm to room temperature and stirred overnight. The crude reaction mixture was washed with a saturated aqueous sodium thiosulfate solution and extracted with diethyl ether. The organic layers were combined and dried with magnesium sulfate. The crude product was loaded onto C18 silica gel and subsequently purified via prep-HPLC to afford **2Neu** (0.089 g, 0.163 mmol, 37 %) as a light yellow solid.
**$^1$H NMR (500 MHz, $CDCl_3$)** δ 8.70 (s, 2H), 7.91 (dd, $J$ = 7.9, 1.2 Hz, 2H), 7.59 (dd, $J$ = 7.7, 1.6 Hz, 2H), 7.38 (td, $J$ = 7.6, 1.1 Hz, 2H), 7.07 (td, $J$ = 7.6, 1.7 Hz, 2H), 2.85 (s, 3H).

**$^{13}$C NMR** (126 MHz, $CDCl_3$, 50°C) δ 151.61, 150.84, 139.14, 133.08, 130.07, 129.60, 128.07, 120.50, 100.78, 98.28, 88.46, 19.86.

**HRMS (ESI pos) m/z for $C_{22}H_{14}I_2N^+$ $[M+H]^+$ :** calculated:545.9210 found: 545.9189

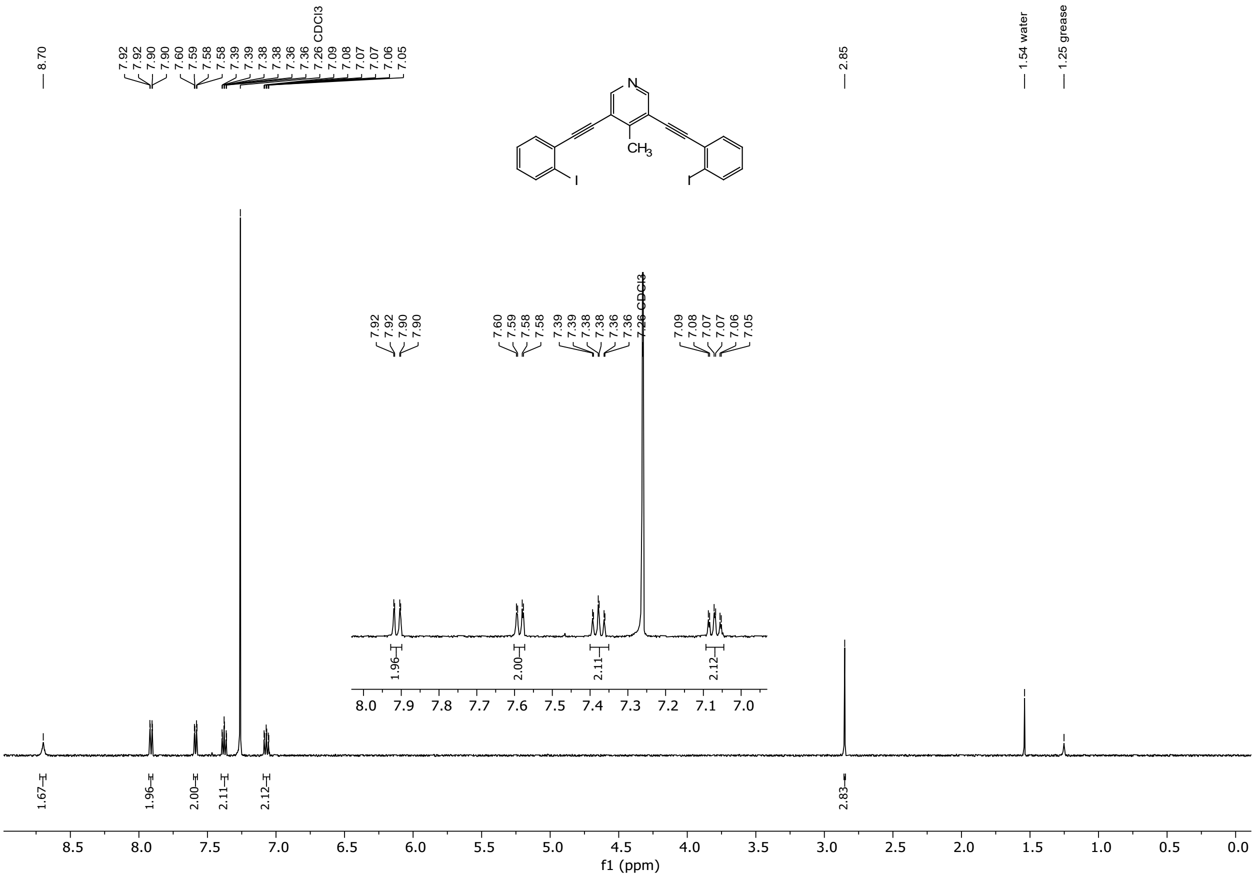

Figure 11S $^1$H NMR spectrum of **2Neu** (500 MHz, $CDCl_3$)

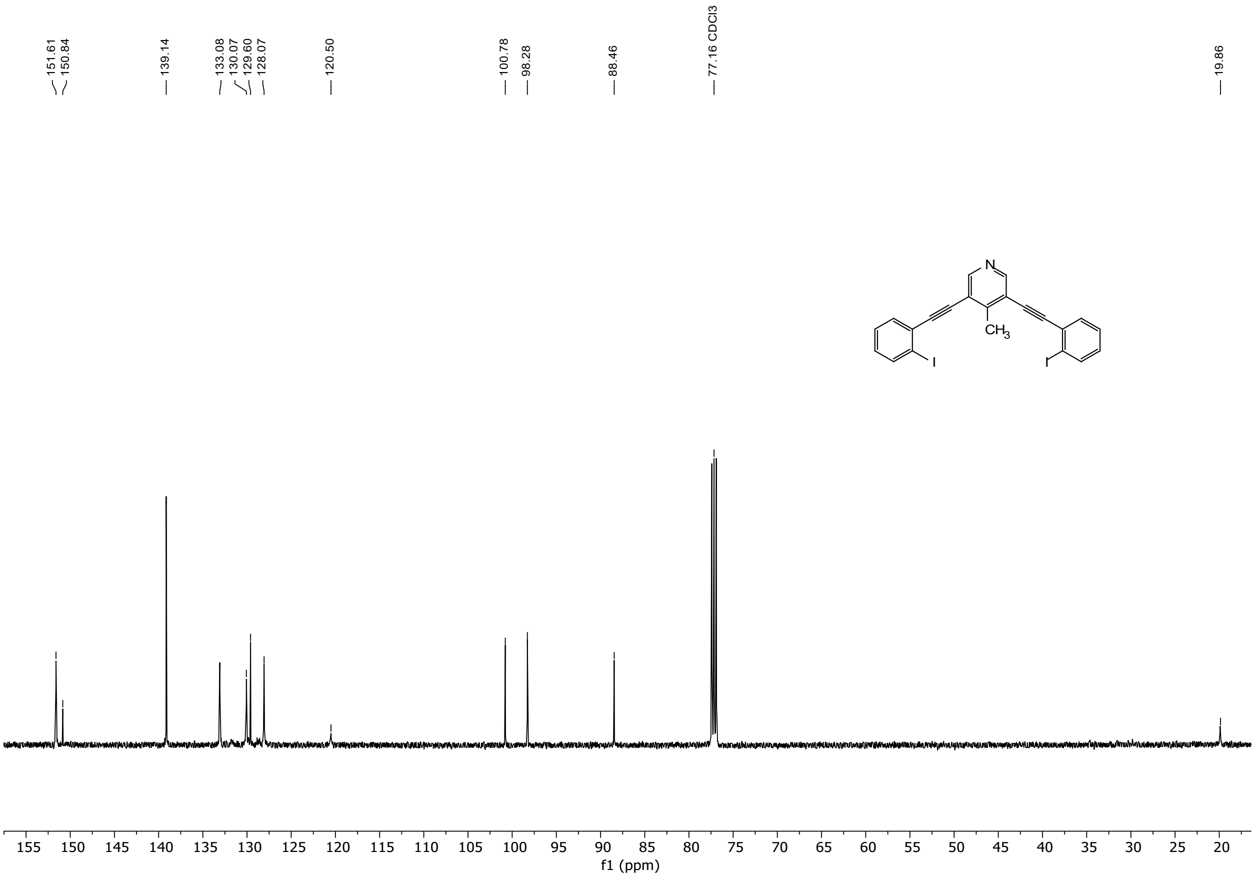


Figure 12S $^{13}$C NMR spectrum of **4Neu** (126 MHz, $CDCl_3$, 50°C)

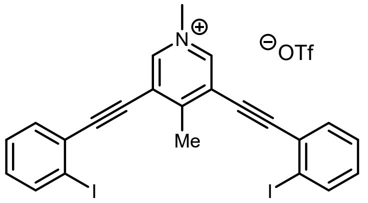


**Procedure:** To a flame dried scintillation vial, **2Neu** (0.089 g, 0.163 mmol) was dissolved in 12ml of dry dichloromethane. Methyl trifluoromethanesulfonate (0.032 g, 0.2 mmol) was added dropwise to the solution, after which the vial was capped then allowed to stir at room temperature for 1-2 days. A white precipitate formed; diethyl ether was added to further the precipitation. The precipitate was isolated by filtration and washed with additional diethyl ether to afford **2** (0.105 g, 0.148 mmol, 91 % yield) as a white solid.

**$^{1}$H NMR (500 MHz, $CD_3CN$)** δ 8.73 (s, 2H), 8.02 (dd, $J$ = 8.0, 1.2 Hz, 2H), 7.72 (dd, $J$ = 7.7, 1.6 Hz, 2H), 7.52 (td, $J$ = 7.6, 1.2 Hz, 2H), 7.26 (td, $J$ = 7.8, 1.6 Hz, 2H), 4.27 (s, 4H), 3.07 (s, 3H).

**$^{13}C$ NMR (126 MHz, $CD_3CN$)** δ 162.01, 146.27, 140.31, 134.74, 132.82, 129.64, 128.36, 125.27, 103.09, 101.01, 84.66, 49.09, 21.76. The $^{13}C$ resonance of the triflate anion (quartet with relative intensities of 1:3:3:1) was not observed)
**$^{19}F$ NMR (470 MHz, $CD_3CN$)** δ -77.56.
**HRMS (ESI pos) m/z for $C_{23}H_{16}I_2N^+$ $[M]^+$ :** calculated: 559.9367 found: 559.9342

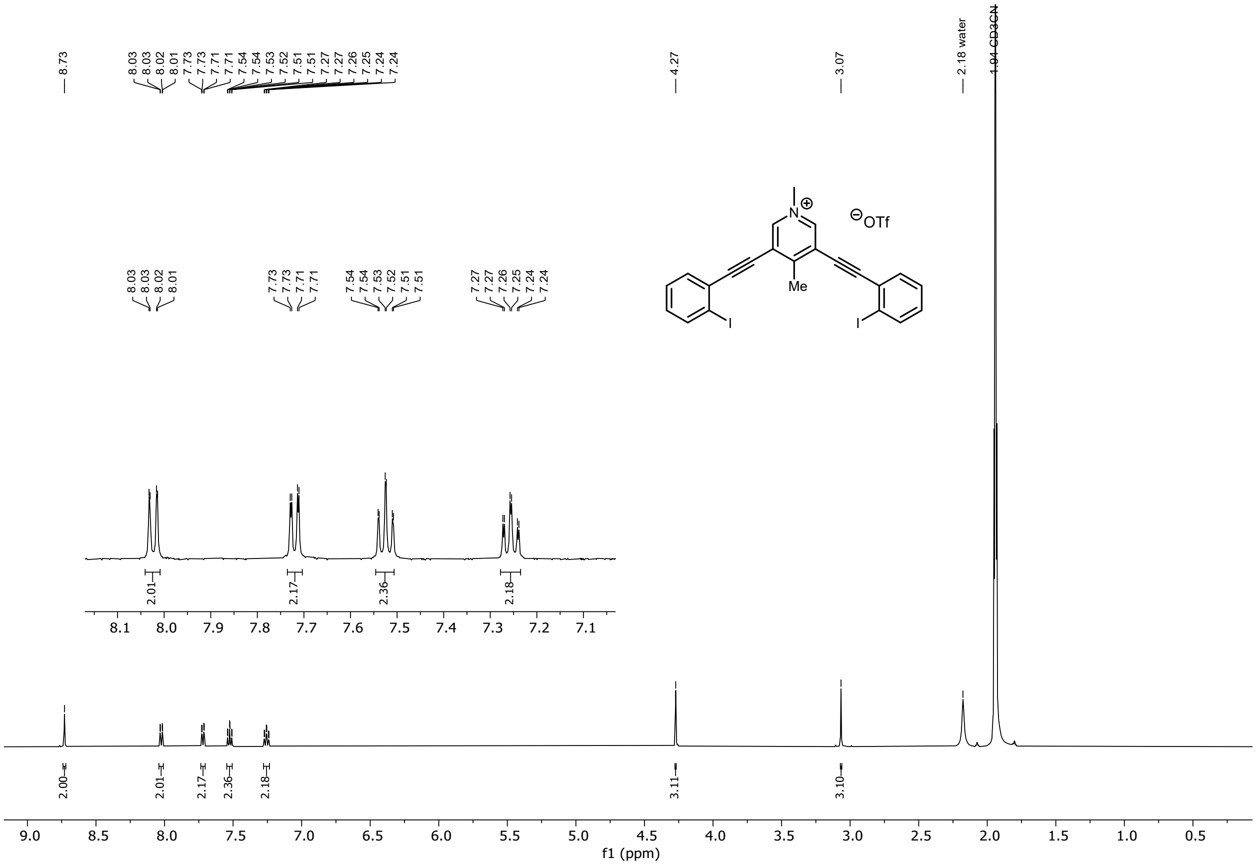


Figure 13S $^1H$ NMR spectrum of **2** (500 MHz, $CD_3CN$)

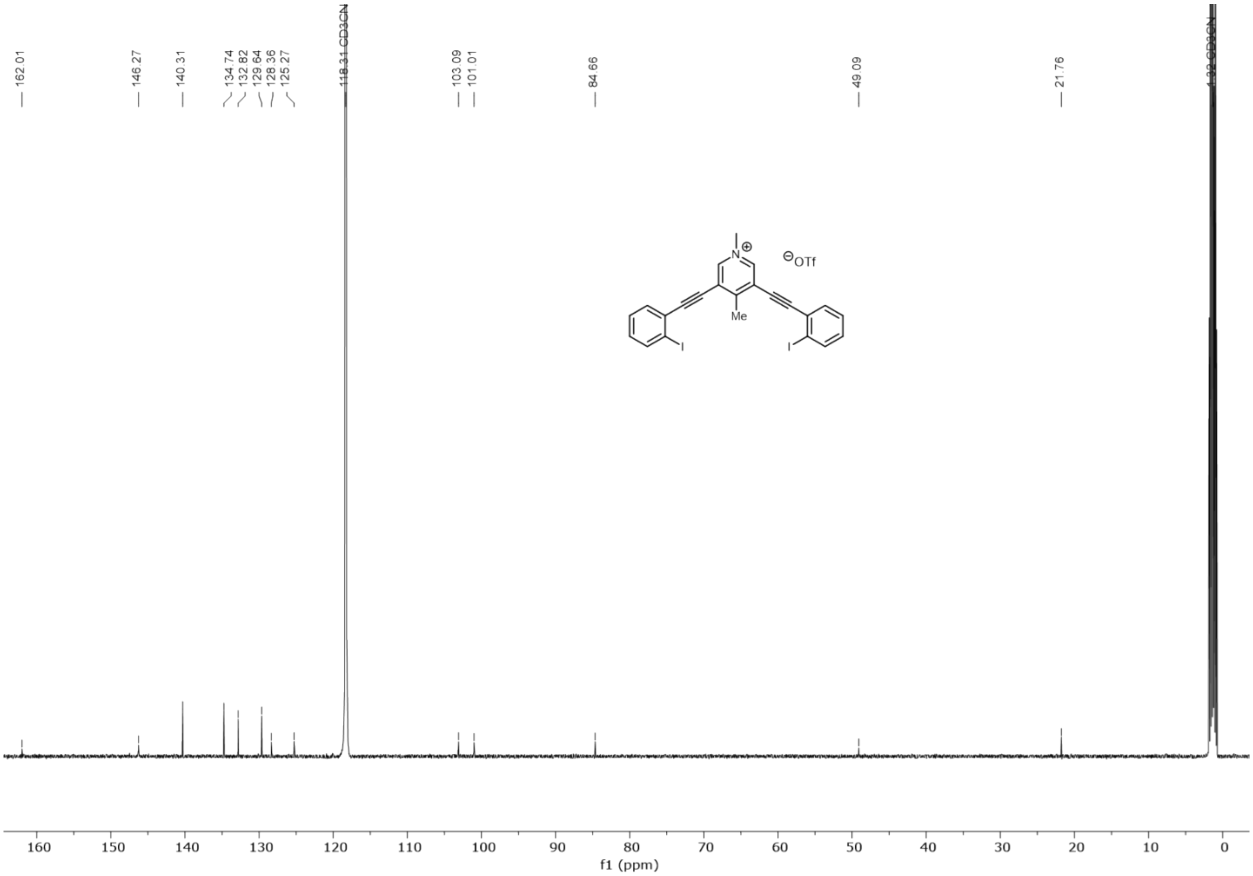


Figure 14S $^{13}C$ NMR spectrum of **2** (126 MHz, $CD_3CN$)

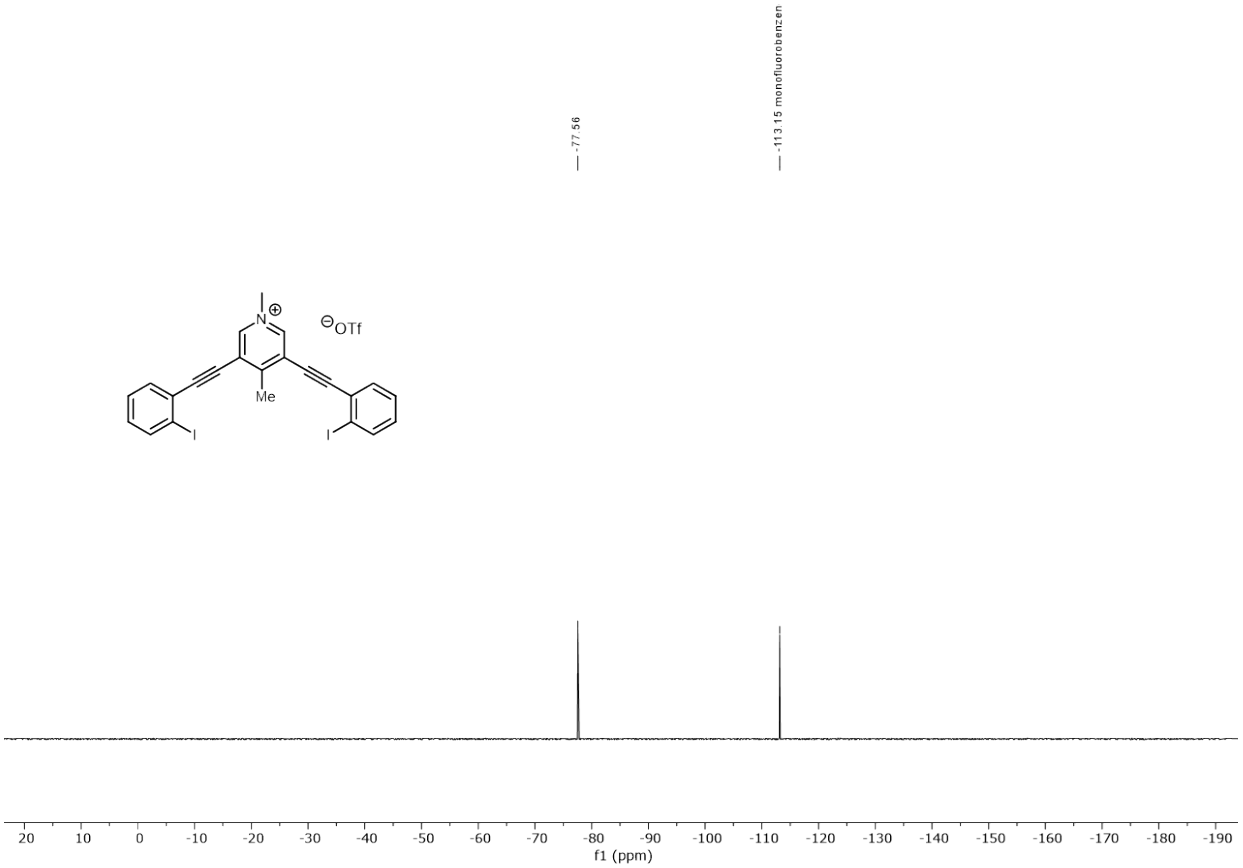


Figure 15S $^{19}F$ NMR spectrum of **2** (470 MHz, $CD_3CN$). $C_6H_5F$ (monofluorobenzene) internal reference.

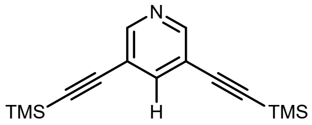


**3CorePro** Synthesis conducted according to previously reported procedure.

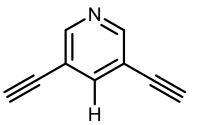


**3CoreDePro** Synthesis conducted according to previously reported procedure.

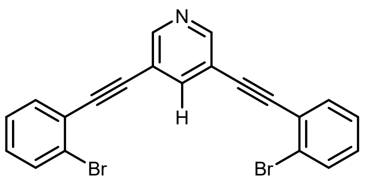

**Procedure:** A flame dried Schlenk flask was charged with 3,5-diethynylpyridine (**3CoreDePro**) (0.16 g, 1.26 mmol), Bis(triphenylphosphine)palladium (II) dichloride (0.035 g, 0.05 mmol) and Copper (I) iodide (0.01 g, 0.05 mmol) and then sealed with a rubber septum. The Schlenk flask was then evacuated and backfilled with dry nitrogen gas three times. To a flame dried 50 ml round bottom flask was added tetrahydrofuran and triethylamine. The round bottom was sealed with a rubber septum and then sparged with dry nitrogen gas for 20 minutes, after which 2-bromo-iodobenzene (0.89 g, 3.14 mmol) was added and sparging resumed for 2 minutes. The 2-bromo-iodobenzene solution was then canula transferred to the Schlenk flask and stirred at room temperature overnight. The crude reaction mixture was concentrated and purified by silica gel column chromatography (5% ethyl acetate in hexanes) to afford **3Br** (0.433 g, 0.99 mmol, 79 %) as a white solid.

**$^{1}$H NMR (500 MHz, $CDCl_3$)** δ 8.75 (d, $J$ = 2.0 Hz, 2H), 8.02 (t, $J$ = 2.0 Hz, 1H), 7.65 (dd, $J$ = 8.1, 1.2 Hz, 2H), 7.58 (dd, $J$ = 7.7, 1.7 Hz, 2H), 7.33 (td, $J$ = 7.6, 1.2 Hz, 2H), 7.24 (td, $J$ = 7.7, 1.6 Hz, 2H).

**$^{13}$C NMR (126 MHz, $CDCl_3$)** δ 151.31, 140.79, 133.58, 132.76, 130.29, 127.32, 125.91, 124.66, 120.00, 91.96, 89.64.

**HRMS (ESI pos) m/z for $C_{21}H_{12}Br_2N^+$ $[M+H]^+$ :** calculated: 437.9311 found: 437.9307

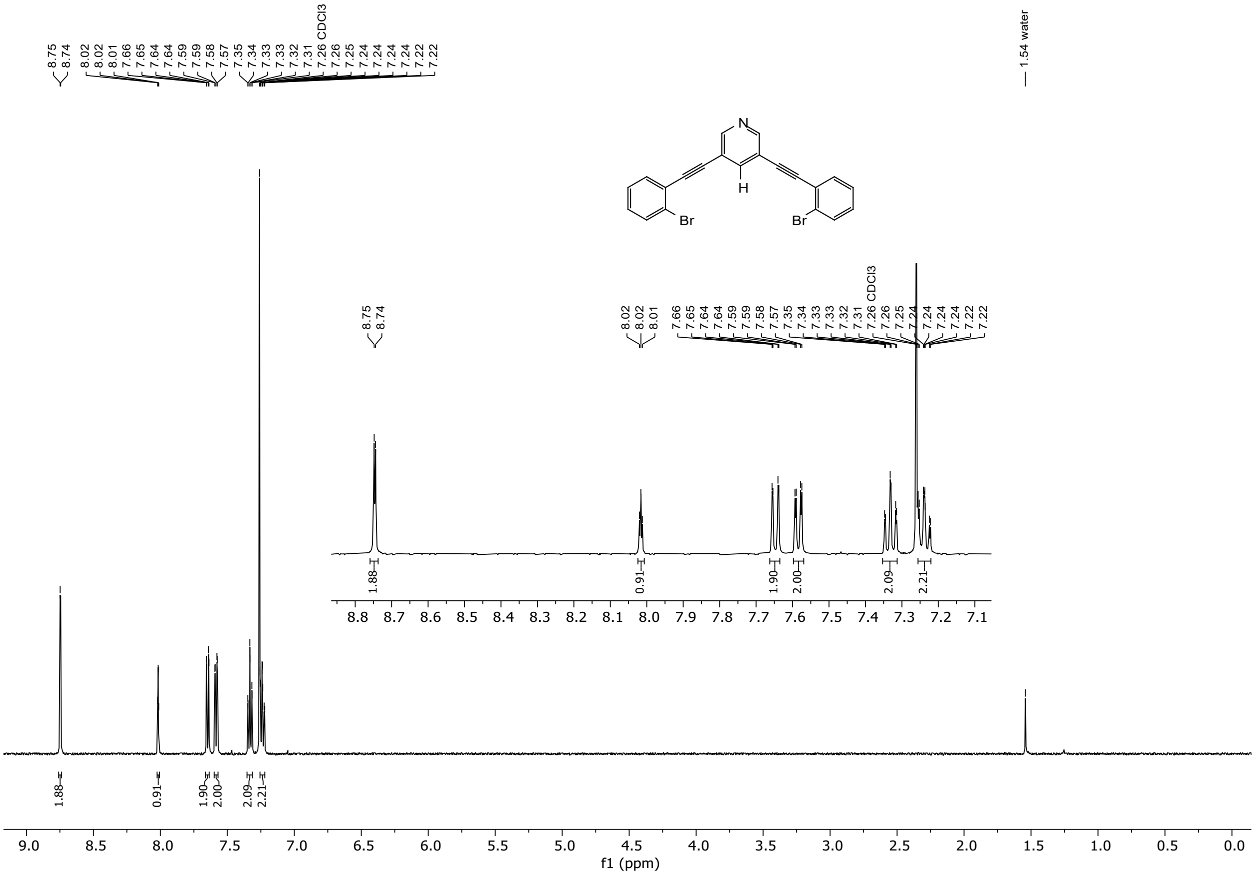


Figure 16S $^{1}$H NMR spectrum of **3Br** (500 MHz, $CDCl_3$)

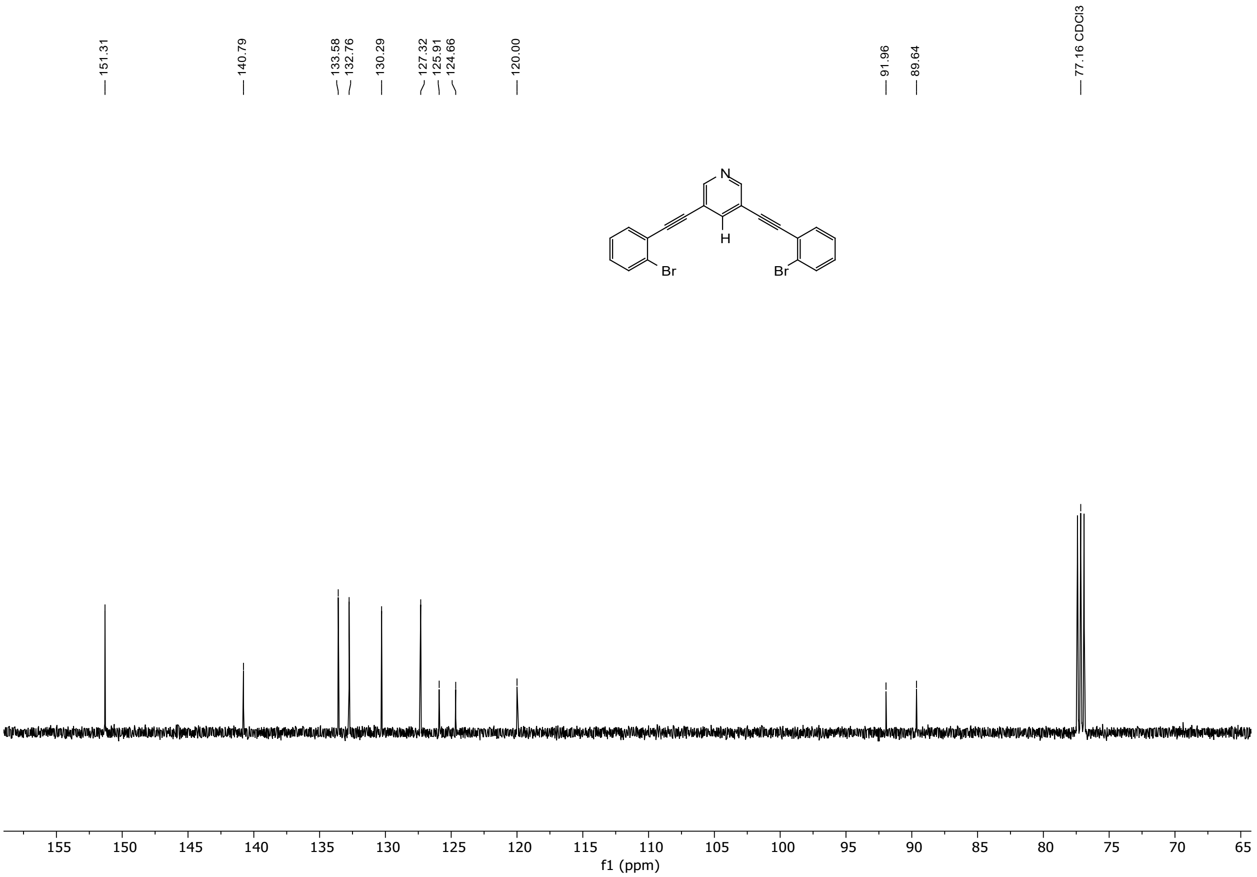

Figure 17S $^{13}$C NMR spectrum of **3Br** (126 MHz, $CDCl_3$)

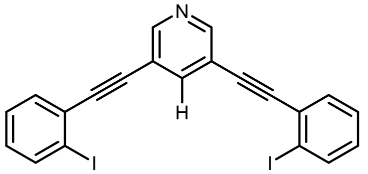


**Procedure:** An oven dried round bottom (50 mL) was charged with **3Br** (0.2 g, 0.46 mmol) was subsequently dissolved in 20 mL of dry tetrahydrofuran and cooled to -67℃ (dry ice and acetone bath). N-butyllithium (1.6 M in hexanes, .072 mL, 1.14 mmol) was added dropwise producing a red solution. The mixture was stirred for 30 min at -67℃. Iodine (0.58 g, 2.29 mmol) in 5 mL of tetrahydrofuran was cooled to -67˚C then added dropwise. The resulting red solution was allowed to gradually warm to room temperature and stirred overnight. The crude reaction mixture was washed with a saturated aqueous sodium thiosulfate solution and extracted with diethyl ether. The organic layers were combined and dried with magnesium sulfate. The crude product was loaded onto C18 silica gel and subsequently purified via prep-HPLC to afford **3Neu** (0.103 g, 0.194mmol, 42%) as a very light yellow solid.

**$^1$H NMR (500 MHz, $CDCl_3$)** δ 8.77 (s, 2H), 8.04 (s, 1H), 7.91 (dd, $J$ = 8.1, 1.2 Hz, 2H), 7.56 (dd, $J$ = 7.7, 1.6 Hz, 2H), 7.37 (t, $J$ = 7.6 Hz, 2H), 7.07 (td, $J$ = 7.7, 1.7 Hz, 2H).

$^{13}C$ NMR (126 MHz, $CDCl_3$, 50°) δ 151.31, 140.56, 139.15, 132.89, 130.22, 129.23, 128.10, 120.17, 101.24, 95.57, 88.96.
**HRMS (ESI pos) m/z for $C_{21}H_{12}I_2N^+$ $[M+H]^+$ :** calculated: 531.9054 found: 531.9018

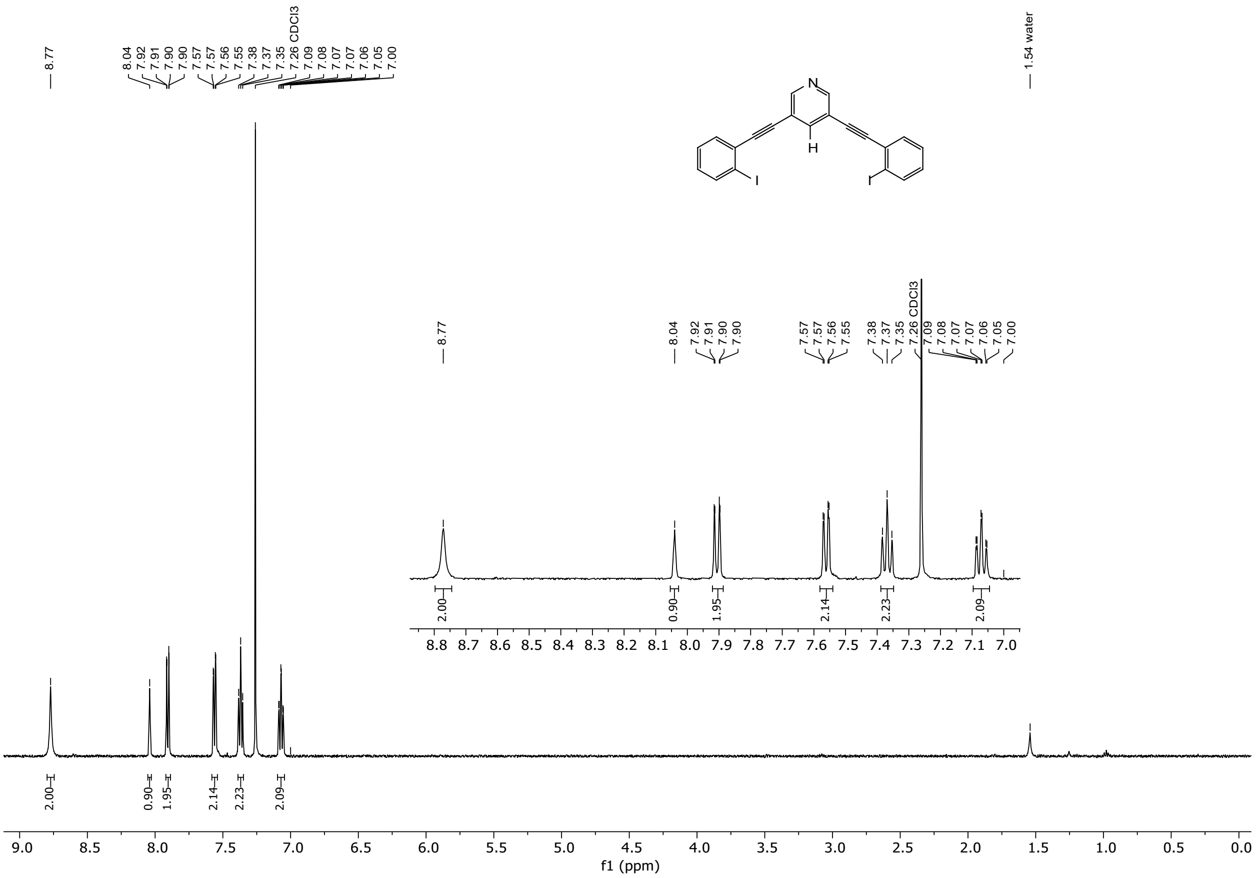

Figure 18S $^{1}H$ NMR spectrum of **3Neu** (500 MHz, $CDCl_3$)

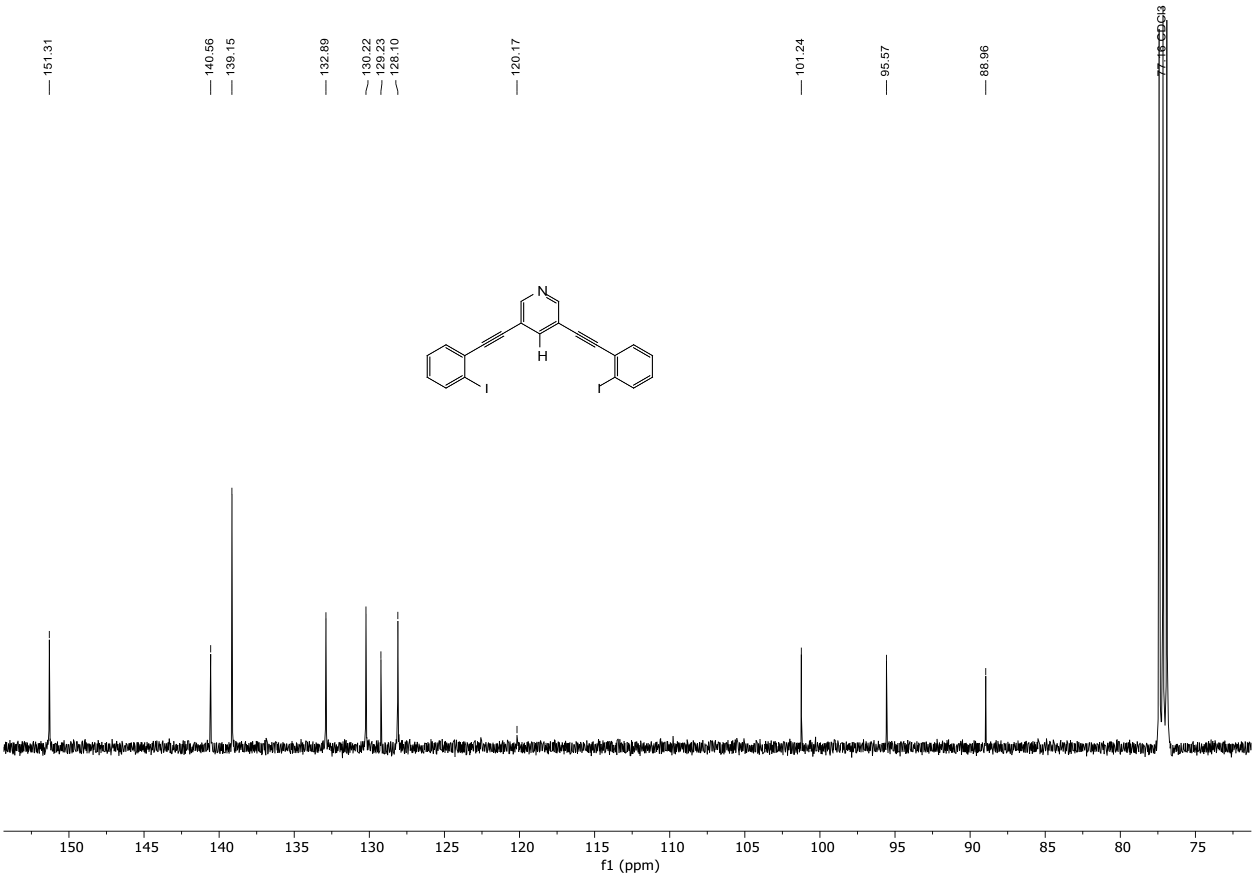


Figure 19S $^{13}C$ NMR spectrum of **3Neu** (126 MHz, $CDCl_3$, 50°C)

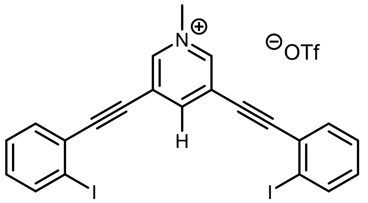


**Procedure:** To a flame dried scintillation vial, **3Neu** (0.05 g, 0.094 mmol) was dissolved in 12ml of dry dichloromethane. Methyl trifluoromethanesulfonate(0.023 g, 0.141 mmol) was added dropwise to the solution, after which the vial was capped then allowed to stir at room temperature for 1-2 days. A white precipitate formed; diethyl ether was added to further the precipitation. The precipitate was isolated by filtration and washed with additional diethyl ether to afford **3** (0.06 g, 0.086 mmol, 91 % yield) as a white solid.

**$^{1}H$ NMR (500 MHz, $CD_3CN$)** δ 8.81 (s, 2H), 8.68 (s, 1H), 8.02 (dd, $J$ = 8.0, 1.1 Hz, 2H), 7.69 (dd, $J$ = 7.7, 1.7 Hz, 2H), 7.52 (td, $J$ = 7.6, 1.2 Hz, 2H), 7.26 (td, $J$ = 7.8, 1.7 Hz, 2H).
**$^{13}C$ NMR (126 MHz, $CD_3CN$)** δ 147.84, 146.09, 139.34, 133.55, 131.93, 128.68, 127.05, 124.22, 100.30, 99.53, 84.13, 48.87. The $^{13}C$ resonance of the triflate anion (quartet with relative intensities of 1:3:3:1) was not observed)
$^{19}F$ NMR (470 MHz, $CD_3CN$) δ -77.55.

**HRMS (ESI pos) m/z for $C_{22}H_{14}I_2N^+$ [M]$^+$ :** calculated: 545.9210 found: 545.9233

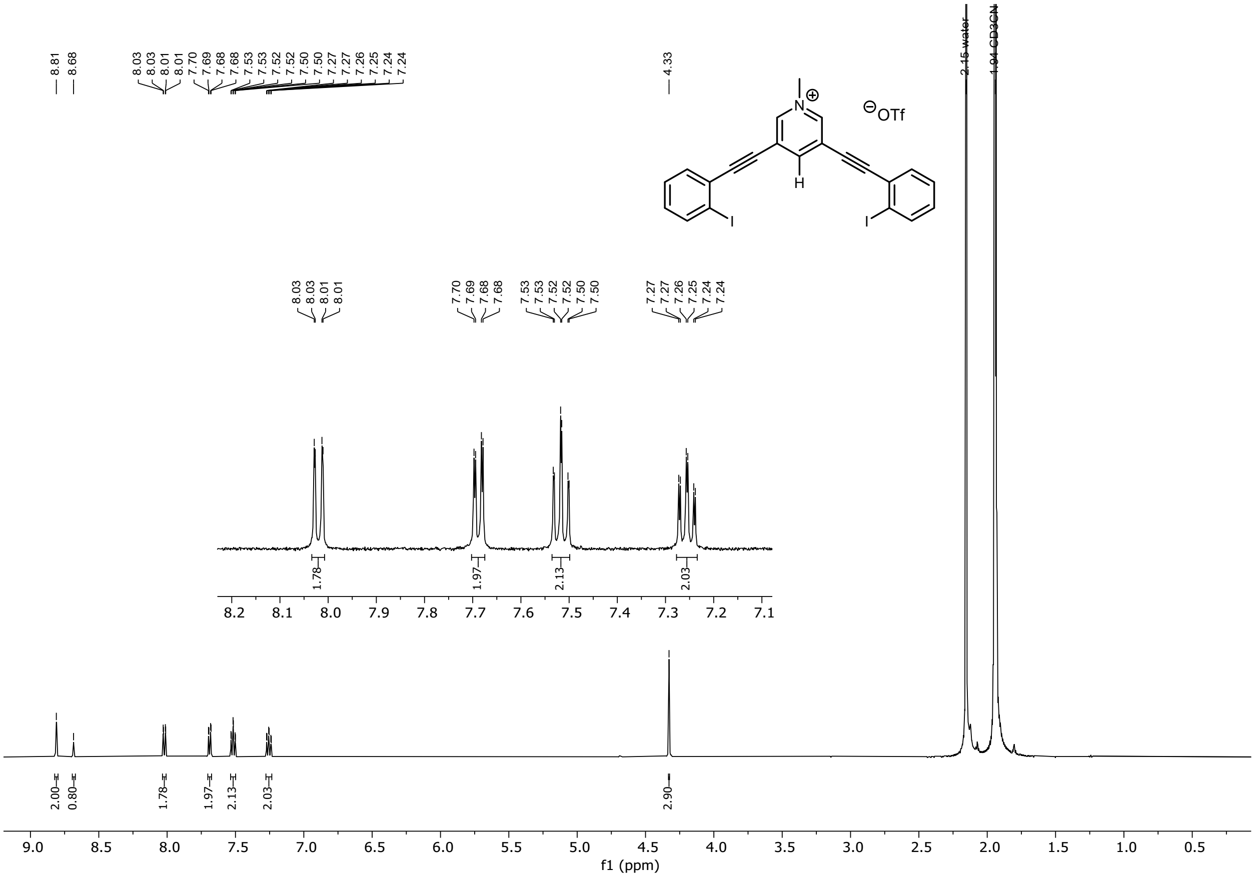

Figure 20S $^1$H NMR spectrum of **3** (500 MHz, $CD_3CN$)

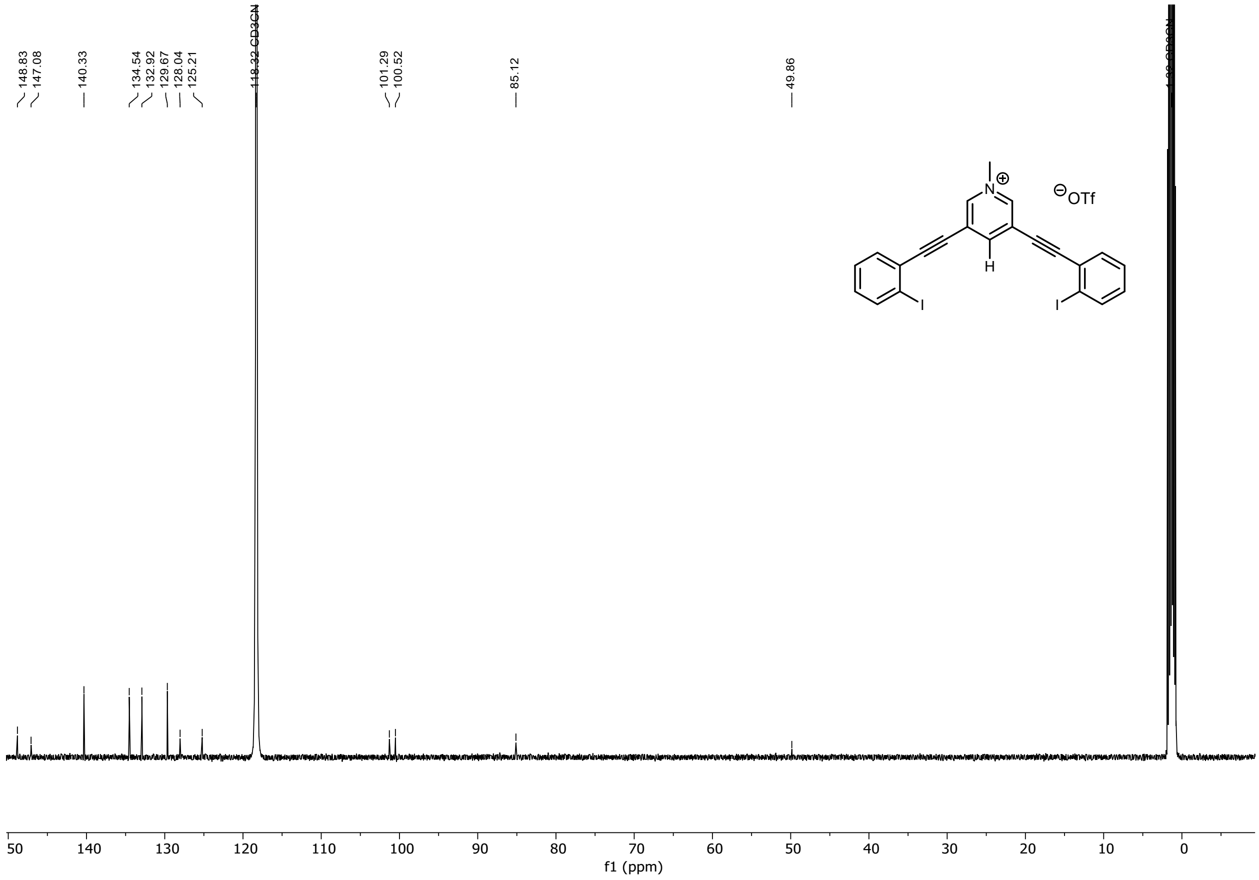


Figure 21S $^{13}$C NMR spectrum of **3** (126 MHz, $CD_3CN$)

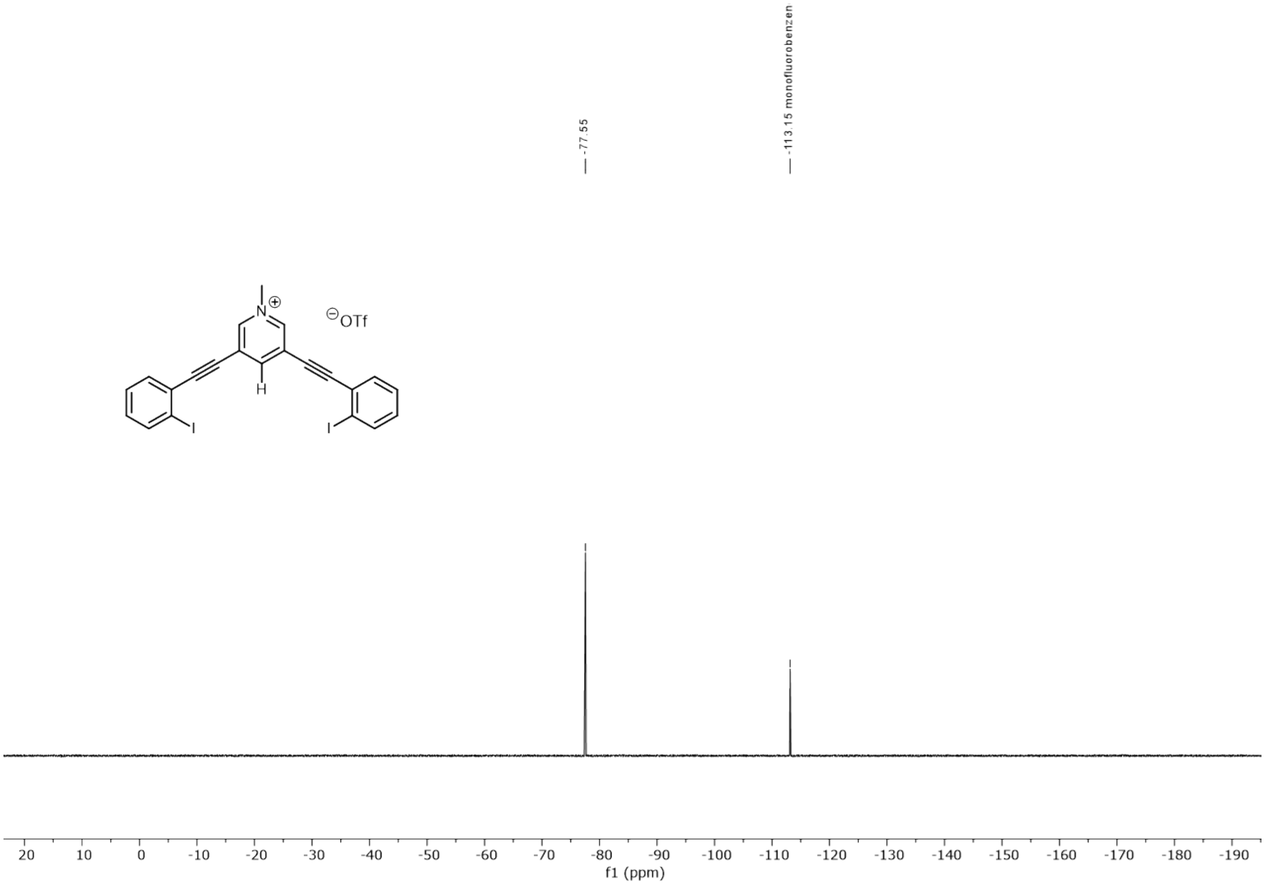

Figure 22S $^{19}$F NMR spectrum of **3** (470 MHz, $CD_3CN$). $C_6H_5F$ (monofluorobenzene) internal reference.

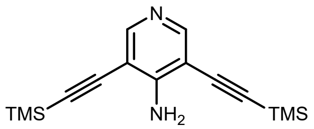


Synthesis adopted from.[10]

An oven dried Schlenk flask was charged with 3,5-dibromo-4-amino-pyridine (9.022 g, 35.7 mmol), bis(triphenylphosphine)palladium(II) dichloride (1.5 g, 2.14 mmol), and  copper (I)

iodide (0.68 g, 3.7 mmol) and subsequently vacuumed and backfilled with dry $N_2$ gas three times. While under nitrogen, the solid reagents were then dissolved in 180ml of dimethylformamide (DMF). To this stirring solution was added N,N-Diisopropylethylamine (31ml, 178.6 mmol) and trimethylsilylacetylene (10.52 g 107.2 mmol). The brown solution was then heated to 60°C and stirred for up to 2 days. Reaction progress was monitored by TLC and the reaction was pulled when consumption of starting material was observed. The reaction mixture was then run through a silica plug with a hexane/ethyl acetate solvent mixture (50:50) to remove any excess salts and catalysts. Subsequent removal of DMF, hexanes and ethyl acetate by roto-evaporation left a brown solid that was purified by silica gel column chromatography (15% ethyl acetate in hexanes) to afford **4CorePro** (5.54 g, 19.33 mmol, 54 % yield) as an off-white solid.

**$^1$H NMR (500 MHz, CDCl$_3$)** δ 8.27 (s, 2H), 5.20 (s, 2H), 0.27 (s, 18H).

**$^{13}$C NMR (126 MHz, CDCl$_3$)** δ 154.24, 151.77, 103.92, 103.55, 97.85, 0.07.

**HRMS (ESI pos) m/z for $C_{15}H_{23}N_2Si_2^+$ [M+H]$^+$ :** calculated:287.1394 found: 287.1409

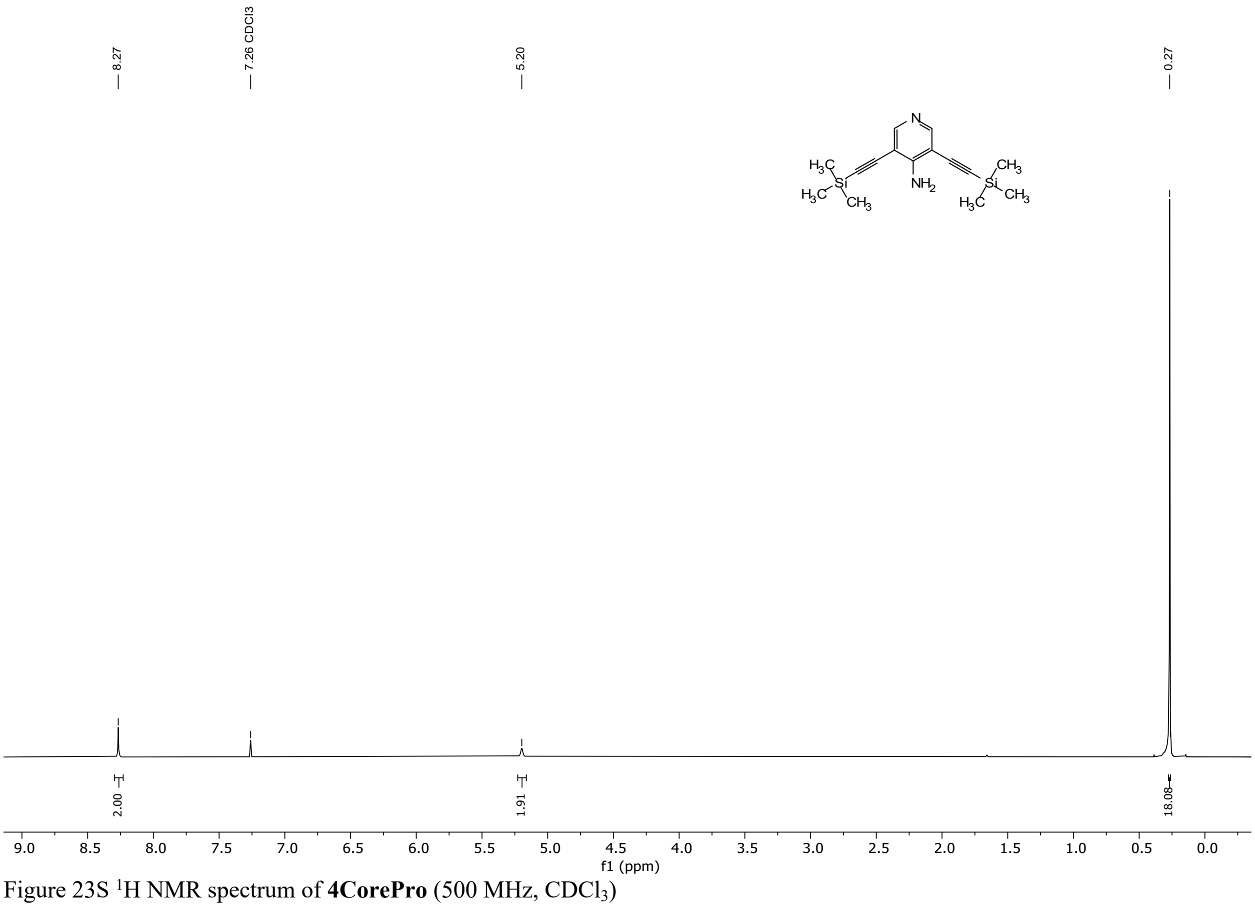


Figure 23S $^1$H NMR spectrum of **4CorePro** (500 MHz, CDCl$_3$)

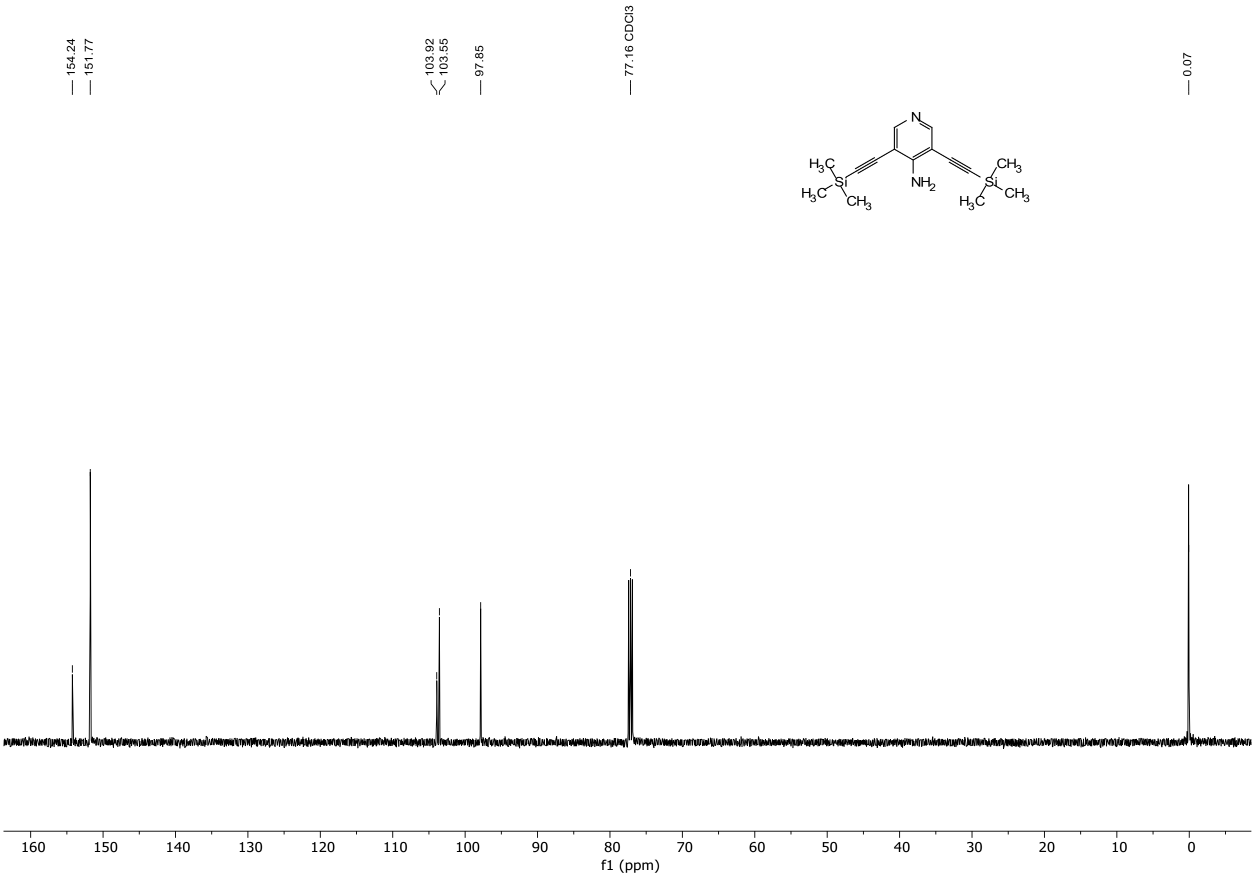

Figure 24S $^{13}$C NMR spectrum of **4CorePro** (126 MHz, $CDCl_3$)

---

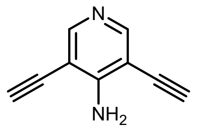


**4CorePro** (1.5 g, 5.24 mmol) was dissolved in 20 mL methanol and 20 mL DCM in a 250 mL round bottom flask. Potassium carbonate (1.81 g, 13.09 mmol) was added to the organic mixture. The reaction stirred vigorously for 4 hours at room temperature. The reaction progress was checked via TLC. When the reaction came to completion, water was added to quench the reaction. The crude product was extracted with ethyl acetate/hexanes (1:1) mix. The organic fractions were then dried over magnesium sulfate and vacuum filtered. The organic solution was reduced under vacuum producing an off-white solid of **4CoreDePro** (0.621 g, 4.37 mmol, 83%). Often the material can then be used in the next step without further purification. If further purification is needed, sublimation is suggested which will produce colorless crystals.

**$^{1}$H NMR (500 MHz, $CDCl_3$)** δ 8.34 (s, 2H), 5.25 (s, 2H), 3.50 (s, 2H).
**$^{13}$C NMR (126 MHz, DMSO)** δ 154.98, 151.89, 102.32, 88.62, 77.11.
**HRMS (ESI pos) m/z for $C_9H_7N_2^+$ $[M+H]^+$ :** calculated: 143.0604 found: 143.0627

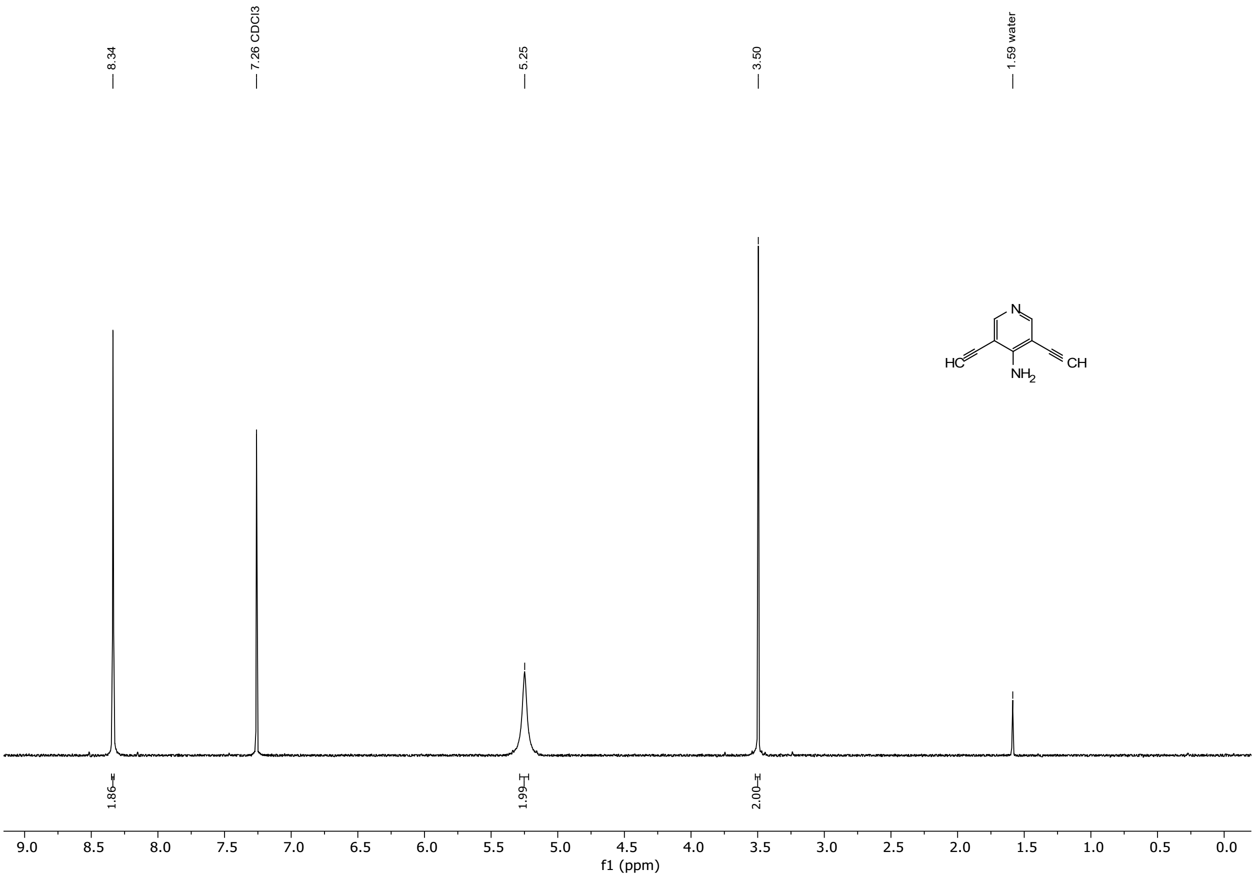


Figure 25S $^1$H NMR spectrum of **4CoreDePro** (500 MHz, $CDCl_3$)

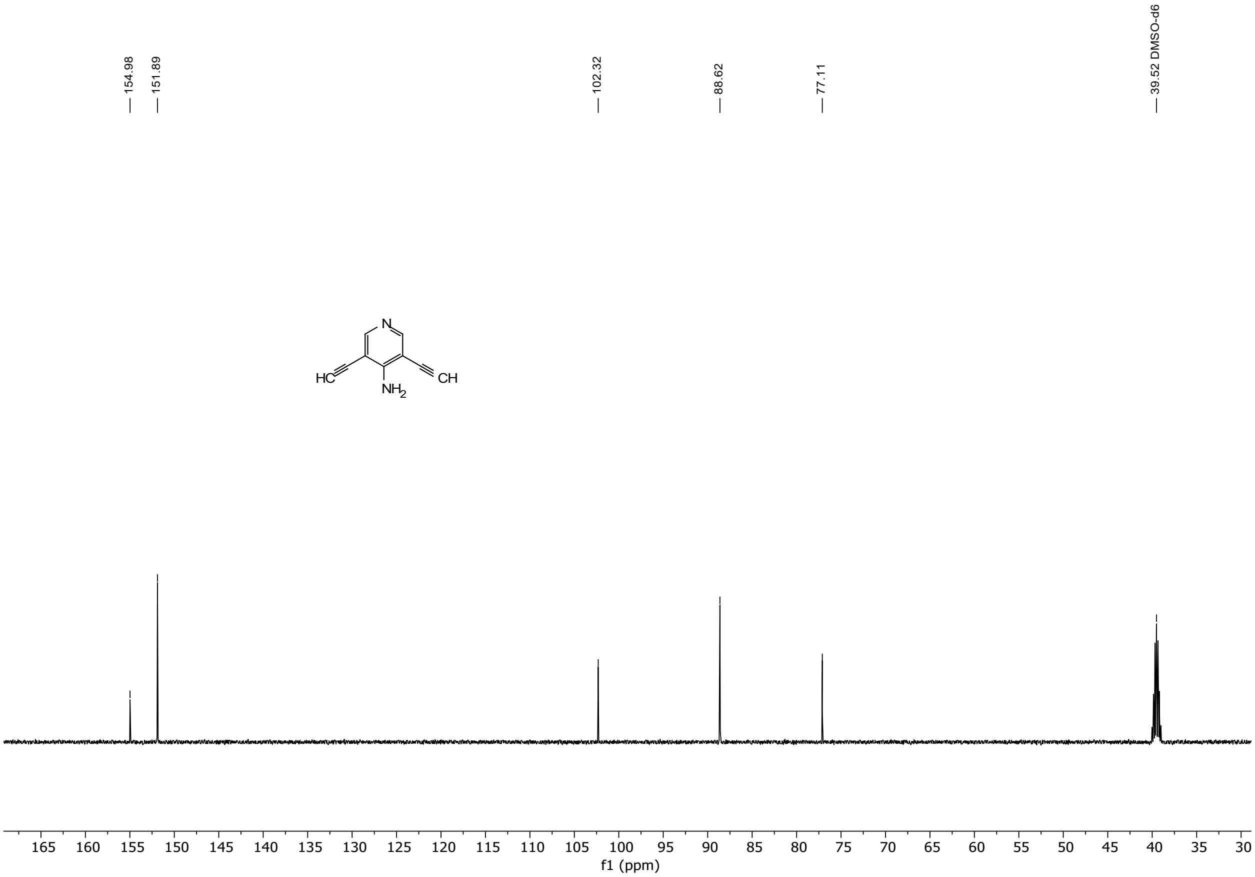

Figure 26S $^{13}C$ NMR spectrum of **4CoreDePro** (126 MHz, $CDCl_3$)

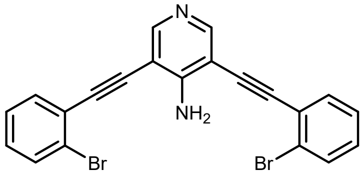


A flame dried Schlenk flask was charged with **4CoreDePro** (0.25 g, 1.76 mmol), Bis(triphenylphosphine)palladium (II) dichloride (0.05 g, 0.07 mmol) and Copper (I) iodide (0.013 g, 0.07 mmol) and then sealed with a rubber septum. The Schlenk flask was then evacuated and backfilled with dry nitrogen gas three times. To a flame dried 50 ml round bottom flask was added tetrahydrofuran and triethylamine. The round bottom was sealed with a rubber septum and then sparged with dry nitrogen gas for 20 minutes, after which 2-bromo-iodobenzene (1.24 g, 4.39 mmol) was added and sparging resumed for 2 minutes. The 2-bromo-iodobenzene solution was then canula transferred to the Schlenk flask and stirred at room temperature overnight. The crude reaction mixture was concentrated and purified by silica gel column chromatography (30% ethyl acetate in hexanes) to afford **4Br** (0.48 g, 1.06 mmol, 60 %) as a white solid.

**$^{1}$H NMR (500 MHz, CDCl$_3$)** δ 8.42 (s, 2H), 7.65 (d, *J* = 7.9 Hz, 2H), 7.60 (d, *J* = 7.9 Hz, 2H), 7.35 (t, *J* = 7.6 Hz, 2H), 7.23 (t, *J* = 7.8 Hz, 2H), 5.77 (s, 2H).
**$^{13}$C NMR** (126 MHz, CDCl$_3$) δ 154.32, 151.69, 133.15, 132.54, 129.98, 127.49, 125.33, 124.91, 103.78, 96.28, 87.25.
**HRMS (ESI pos) m/z for $C_{21}H_{13}Br_2N_2^+$ $[M+H]^+$ :** calculated:452.9420 found:452.9448

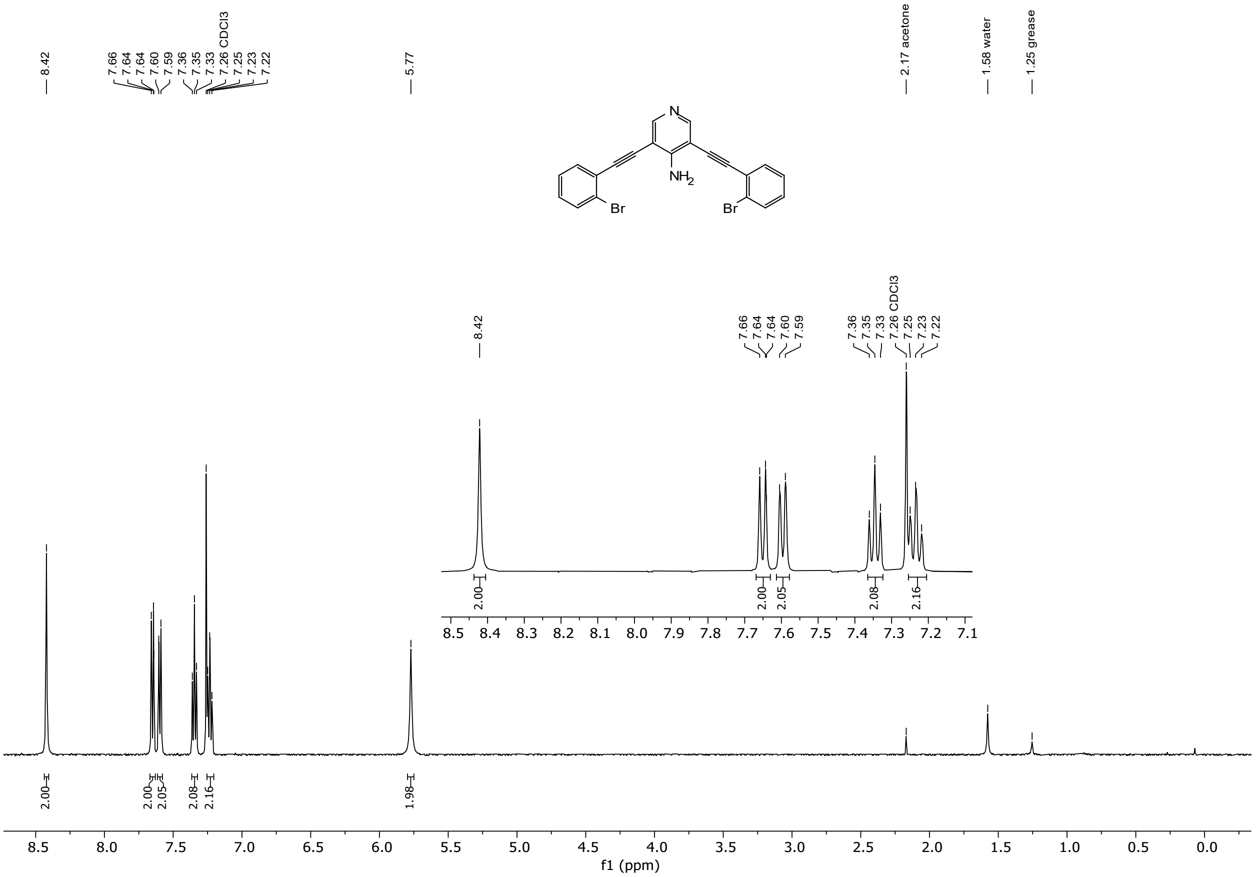


Figure 27 $^{1}$H NMR spectrum of **4Br** (500 MHz, CDCl$_3$)

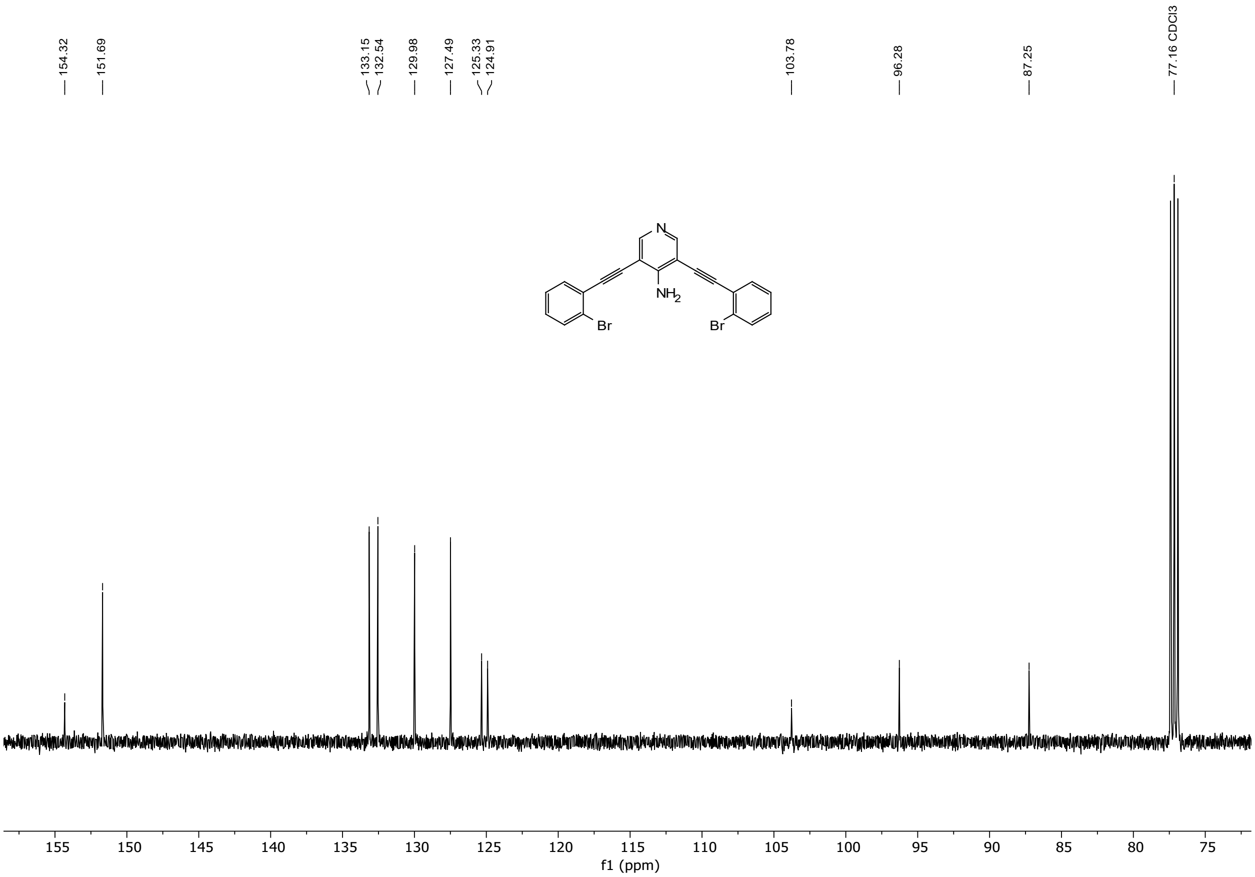


*Figure 28S* $^{13}$C NMR spectrum of **4Br** (126 MHz, $CDCl_3$)

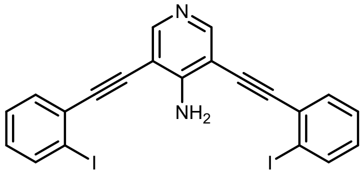


**4Br** (0.1 g, 0.22 mmol), copper iodide (0.004 g, 0.022 mmol), sodium iodide (0.13 g, 0.88 mmol) were added to a 10-20 mL microwave reaction vial containing a stir bar and dissolved in 10-15ml 1,4-dioxane. To the reaction mixture, transN,N'-dimethylcyclohexane-1,2-diamine (0.15 mL, 0.95 mmol) was added. The microwave vial was sealed and placed in a microwave. The reaction was performed in a Biotage Initiator+ microwave reactor for 16 hours at 150 ˚C. After cooling, an aliquot was run through pipet silica plug with EtOAc to remove catalysts and salts. The EtOAc crude was then ran through GCMS in order to obtain % conversion of bromines to iodines. If the reaction was unfinished, it would be submitted again at 30 min increments. When the reaction ran to completion, the crude reaction was run through a silica plug with EtOAc. The crude product was loaded onto C18 silica gel and subsequently purified via prep-HPLC to afford **4Neu** (0.028 g, 0.051 mmol, 23 % yield) as a beige solid.

**$^1$H NMR (500 MHz, CDCl$_3$) δ** 8.44 (s, 2H), 7.90 (dd, *J* = 8.1, 1.2 Hz, 2H), 7.58 (dd, *J* = 7.8, 1.6 Hz, 2H), 7.38 (td, *J* = 7.6, 1.2 Hz, 2H), 7.07 (td, *J* = 7.9, 1.7 Hz, 2H), 5.86 (s, 2H).
**$^{13}$C NMR (126 MHz, CDCl$_3$, 45°C)** δ 154.39, 151.91, 138.87, 132.74, 129.93, 129.52, 128.24, 103.89, 100.77, 99.49, 86.57.
**HRMS (ESI pos) m/z for $C_{21}H_{13}I_2N_2^+$ [M+H]$^+$ :** calculated: 546.9163 found: 546.9183

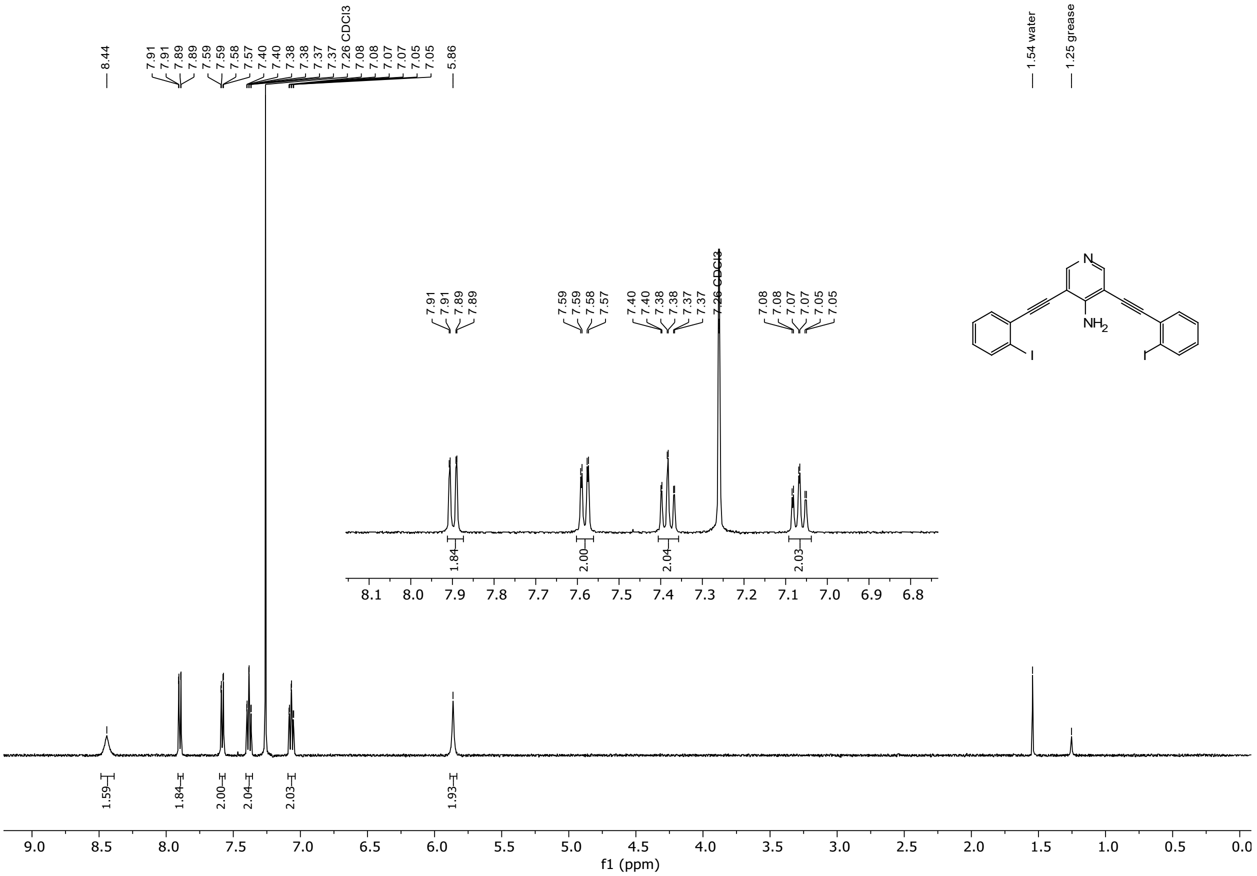

Figure 29S $^1$H NMR spectrum of **4Neu** (500 MHz, CDCl$_3$)

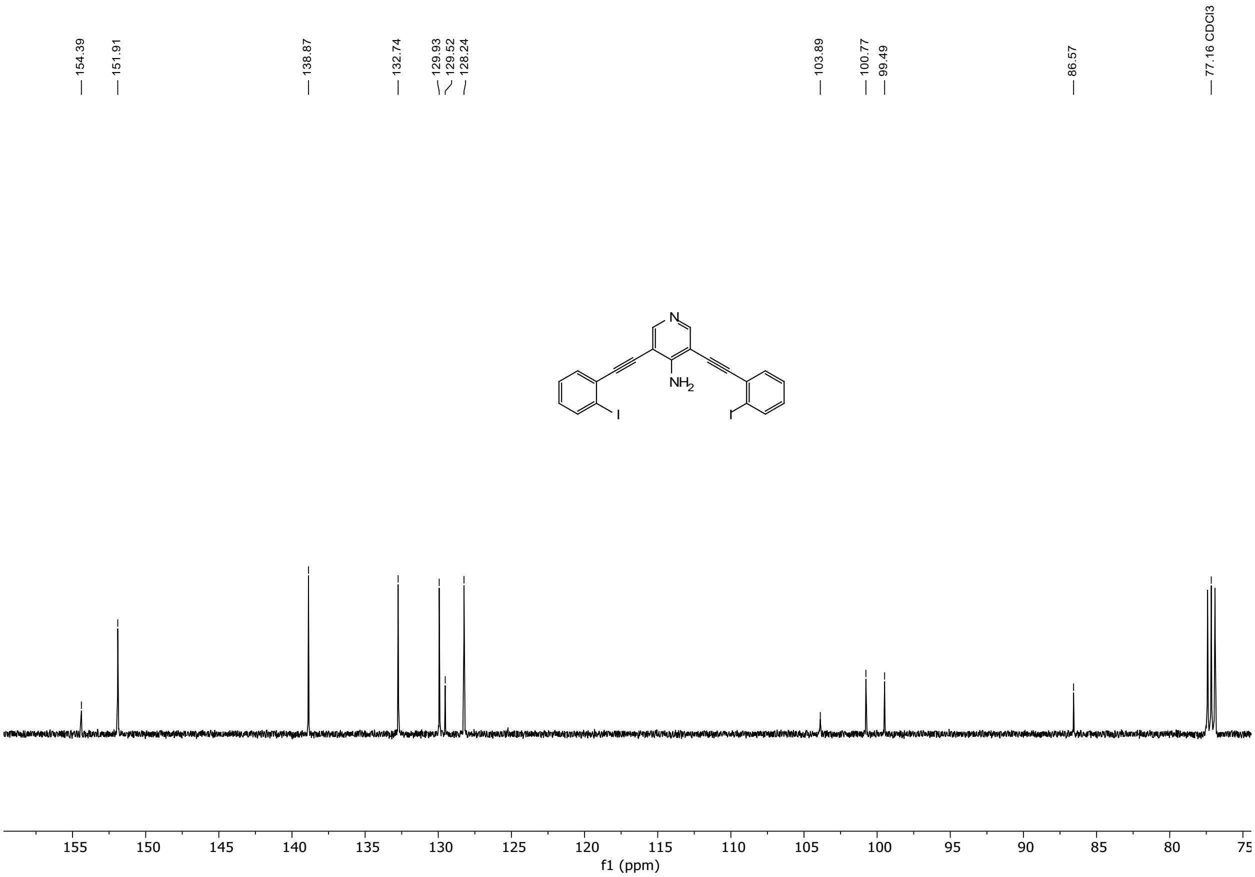


Figure 30S $^{13}C$ NMR spectrum of **4Neu** (126 MHz, $CDCl_3$, 45°C)

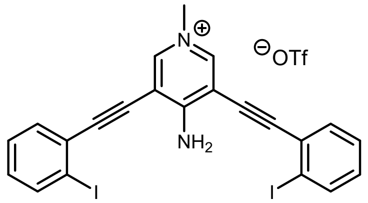


To a flame dried scintillation vial, **4Neu** (0.07 g, 0.128 mmol) was dissolved in 5 ml of dry dichloromethane. Methyl trifluoromethanesulfonate(0.033 g, 0.2 mmol) was added dropwise to the solution, after which the vial was capped then allowed to stir at room temperature for 1-2 days. A white precipitate formed; diethyl ether was added to further the precipitation. The precipitate was isolated by filtration and washed with additional diethyl ether to afford **4** (0.063 g, 0.089 mmol, 69 % yield) as a white solid.

**$^{1}$H NMR (500 MHz, DMSO)** δ 8.66 (s, 2H), 8.47 (s, 2H), 8.02 (dt, $J$ = 8.1, 1.2 Hz, 2H), 7.82 (dt, $J$ = 7.8, 1.4 Hz, 2H), 7.54 (tt, $J$ = 7.6, 1.3 Hz, 2H), 7.25 (tt, $J$ = 7.5, 1.5 Hz, 2H), 3.99 (s, 3H).
**$^{13}$C NMR (126 MHz, DMSO)** δ 156.71, 145.78, 138.70, 133.70, 131.37, 128.33, 127.41, 104.18, 101.46, 100.43, 82.09, 45.17. The $^{13}$C resonance of the triflate anion (quartet with relative intensities of 1:3:3:1) was not observed)
**$^{19}$F NMR** (470 MHz, $CD_3CN$) δ -77.57.

**HRMS (ESI pos) m/z for $C_{22}H_{15}I_2N_2^+$ [M]$^+$ :** calculated:560.9319 found: 560.9288

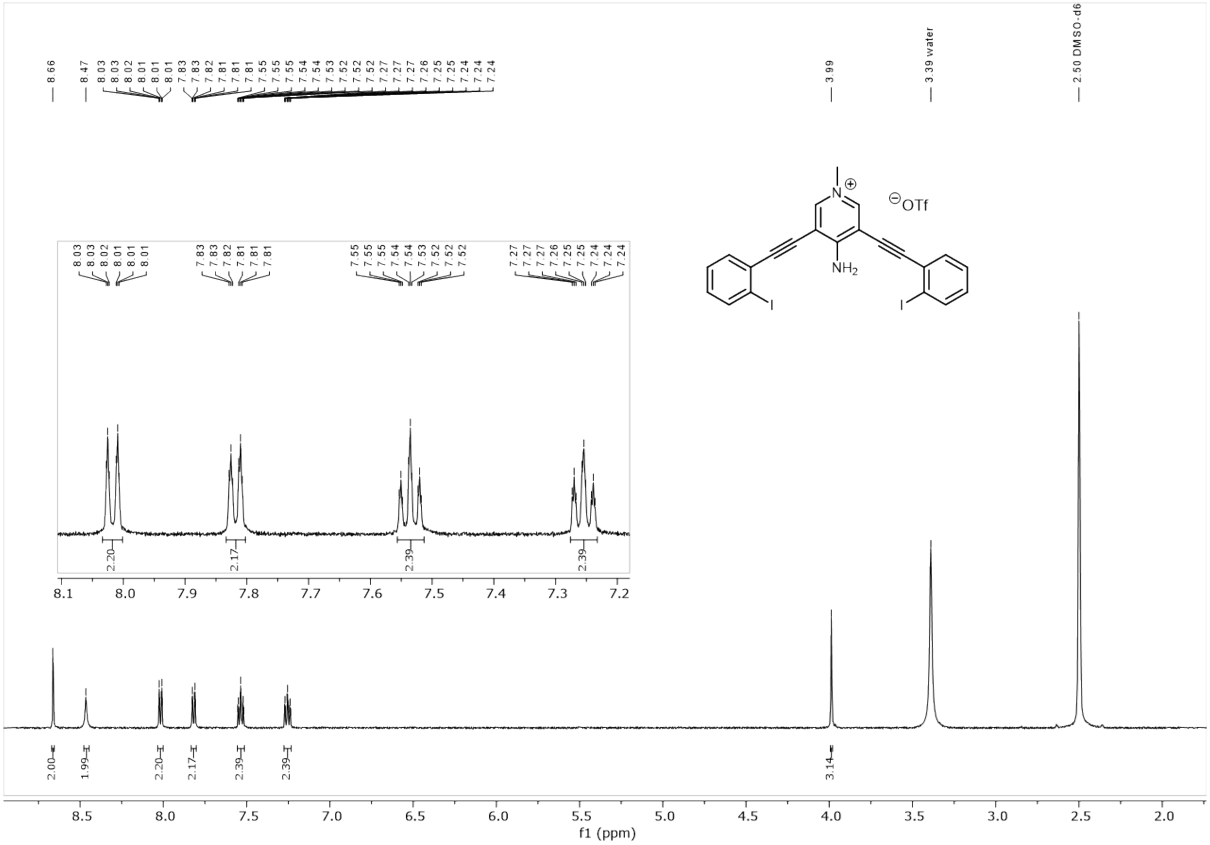


Figure 31S $^1$H NMR spectrum of **4** (500 MHz, DMSO)

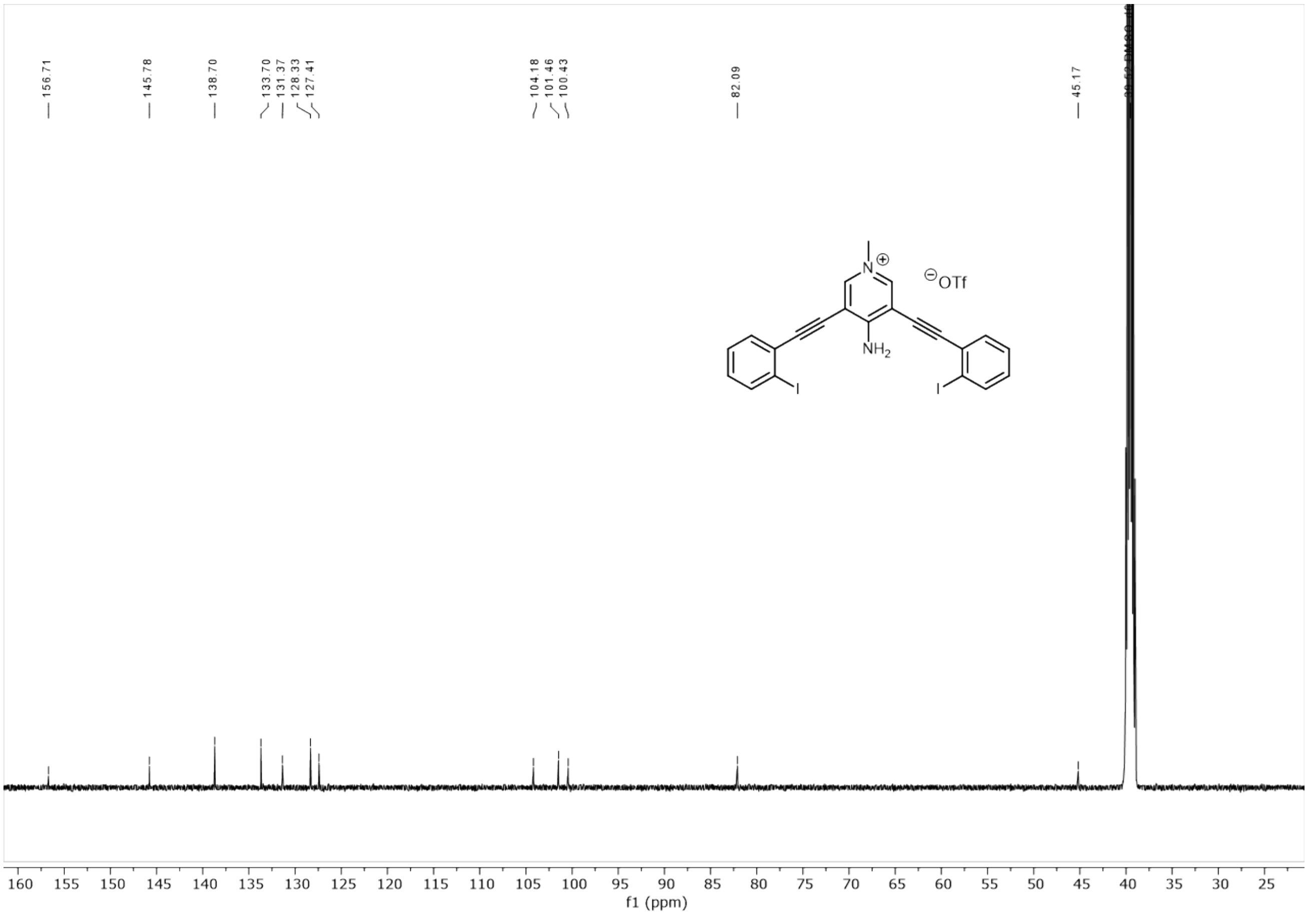

Figure 32S $^{13}$C NMR spectrum of **4** (126 MHz, DMSO)

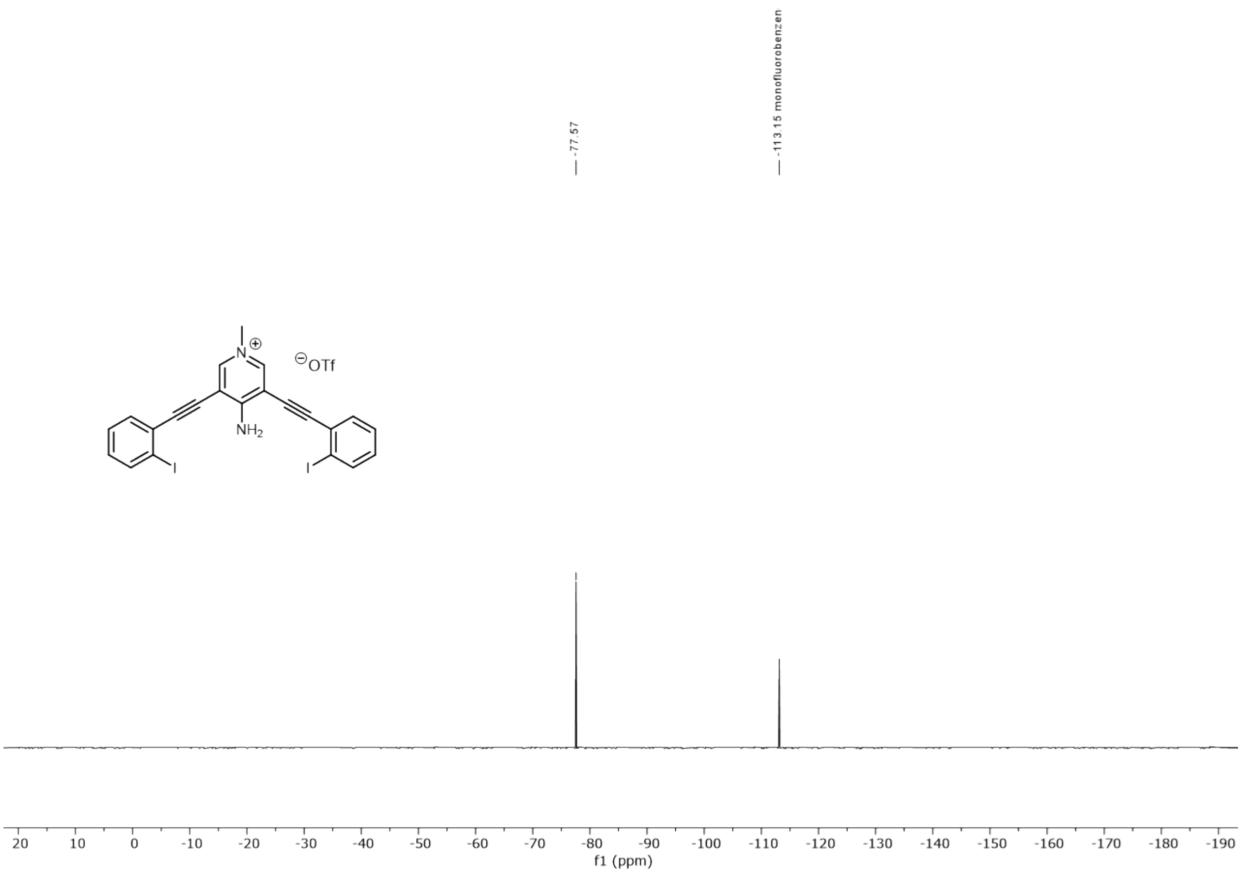

Figure 33S $^{19}$F NMR spectrum of **4** (470 MHz, $CD_3CN$). $C_6H_5F$ (monofluorobenzene) internal reference.

## Computational details

Gas-phase density functional theory (DFT) calculations were performed with the M06-2X functional[11] using the Gaussian 09 suite of programs.[12] The def2-TZVPP basis set[13] was used for all atoms and for iodine a small-core energy-consistent relativistic effective core potential (def2-ECP)[14] was applied to iodine. The basis set and effective core potential was downloaded from the EMSL Basis Set Exchange.[15] Employing optimized geometries, frequency calculations were carried out confirm molecules and complexes were at local minima. The electrostatic potential surface maps (isodensity = 0.001au)were constructed using optimized structures (coordinates below) The interaction energy was computed as the difference in energy between the complex and the sum of the energies of the individual components in their complex geometry (equation below) and were corrected for basis set superposition error (BSSE) by the counterpoise technique.[16] The alkyne driver study was conducted using a relaxed scan procedure and utilized 8 steps with each step being 45 degrees.

Binding energy (complexation energy) is the energy of the complex minus the energy of the isolated monomers (host & guest) in their minima configuration.

Table 2S Gas phase complex results. All values in kcal/mol. Deformation energy is the difference between complexation energy and interaction energy.

| Scaffold | Complexation energy | Interaction energy | Deformation energy |
|---|---|---|---|
| **1** | -76.02 | -77.36 | 1.34 |
| **2** | -69.05 | -70.37 | 1.32 |
| **3** | -65.95 | -67.11 | 1.16 |
| **4** | -74.28 | -76.70 | 2.43 |
| **2-tridendate** | -69.68 | -71.49 | 1.81 |

Table 3S Alkyne Driver Study Results—Relative energy in kcal/mol

| | Receptor | | | |
|---|---|---|---|---|
| Dihedral | **1** | **2** | **3** | **4** |
| 0 | 0.00 | 0.04 | 0.34 | 0.00 |
| 45 | 0.89 | 0.48 | 0.95 | 0.79 |
| 90 | 2.25 | 1.31 | 1.52 | 2.58 |
| 135 | 1.54 | 0.59 | 0.56 | 2.52 |
| 180 | 0.93 | 0.00 | 0.00 | 2.35 |
| 225 | 1.54 | 0.60 | 0.82 | 2.47 |
| 270 | 2.25 | 1.31 | 1.53 | 2.68 |
| 315 | 0.89 | 0.48 | 0.71 | 1.13 |
| 360 | 0.00 | 0.04 | 0.34 | 0.00 |

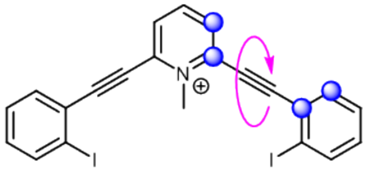


Chemdraw depicting the alkyne bond that provided the rotation axis. The carbon atoms labeled as blue circles were used to dictate the dihedral angle.

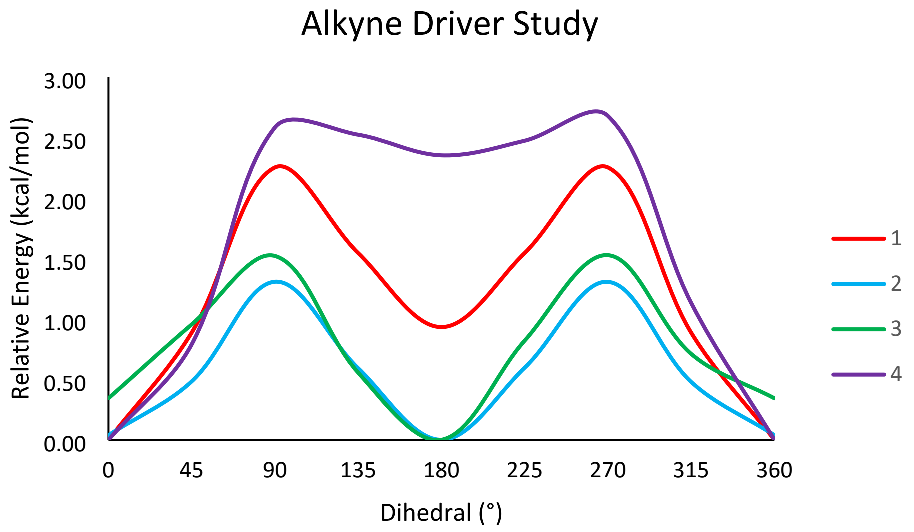


Figure 34S. Alkyne driver analysis plot.

# Optimized coordinates for single point conformational analysis and MEP analysis

## Bidentate

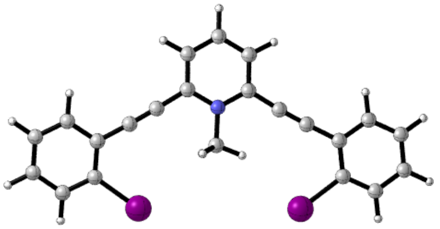

**1**

| | | | |
|---|---|---|---|
| I | 3.18164000 | -1.88927100 | -0.00034300 |
| I | -3.12845200 | -1.86371400 | 0.00010800 |
| C | 7.22859600 | -0.37989800 | -0.00005400 |
| C | -7.21911500 | -0.47739700 | -0.00024200 |
| C | 7.09929900 | 1.00291200 | 0.00017800 |
| H | 7.97687100 | 1.63322500 | 0.00029100 |
| C | 5.84154700 | 1.57222200 | 0.00026100 |
| H | 5.72238200 | 2.64662800 | 0.00044100 |
| C | 6.10515200 | -1.19545800 | -0.00020100 |
| H | 6.21479300 | -2.27020900 | -0.00038100 |
| C | 4.84157400 | -0.62845700 | -0.00011600 |
| C | 4.69490100 | 0.76589200 | 0.00011700 |
| C | 3.42232200 | 1.39673800 | 0.00021400 |

| | | | |
|---|---|---|---|
| C | 2.36024800 | 1.96782400 | 0.00028700 |
| C | 1.15619600 | 2.70778300 | 0.00028900 |
| C | 1.18146100 | 4.09824600 | 0.00031900 |
| H | 2.14160000 | 4.58983900 | 0.00035200 |
| C | -0.00324600 | 4.80961500 | 0.00029900 |
| C | -1.20923500 | 4.13315700 | 0.00024100 |
| H | -2.15590700 | 4.65032000 | 0.00021300 |
| N | -0.03940100 | 2.05725500 | 0.00025200 |
| C | -1.21599000 | 2.74487000 | 0.00021400 |
| C | -2.42239800 | 2.01154700 | 0.00014200 |
| C | -3.46777400 | 1.40982000 | 0.00006400 |
| C | -4.72156000 | 0.74402200 | -0.00003900 |
| C | -5.89179900 | 1.51597600 | -0.00013500 |
| H | -5.80459700 | 2.59343500 | -0.00012800 |
| C | -7.13161000 | 0.90885600 | -0.00023600 |
| H | -8.02787300 | 1.51228400 | -0.00031000 |
| C | -4.82602900 | -0.65438300 | -0.00004600 |
| C | -6.07192700 | -1.25914300 | -0.00014700 |
| H | -6.14930300 | -2.33669000 | -0.00014900 |
| H | 0.01306800 | 5.89063900 | 0.00032200 |
| H | 8.21034400 | -0.83315000 | -0.00012100 |
| H | -8.18687900 | -0.95980000 | -0.00031900 |
| C | -0.10220300 | 0.58608700 | 0.00023100 |
| H | 0.90380100 | 0.18489600 | 0.00042700 |
| H | -0.64579900 | 0.26050700 | -0.88412900 |
| H | -0.64614800 | 0.26050300 | 0.88437300 |

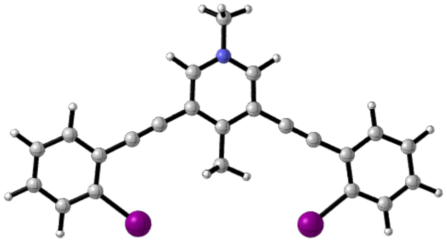

**2**

| | | | |
|---|---|---|---|
| I | 3.38715000 | -2.09583900 | 0.00219000 |
| I | -3.32807700 | -2.06621000 | 0.00111900 |
| C | 7.37931000 | -0.44991700 | -0.00193600 |
| C | -7.37472300 | -0.55613300 | -0.00093300 |
| C | 7.20233400 | 0.92734600 | -0.00391300 |
| H | 8.05772800 | 1.58762700 | -0.00527200 |
| C | 5.92533600 | 1.45305200 | -0.00411500 |
| H | 5.77129900 | 2.52303800 | -0.00561500 |
| C | 6.28294600 | -1.30024400 | -0.00017000 |
| H | 6.42659800 | -2.37092000 | 0.00136500 |
| C | 5.00001100 | -0.77577700 | -0.00038900 |
| C | 4.80475400 | 0.61181700 | -0.00237900 |

| | | | |
|---|---|---|---|
| C | 3.50919800 | 1.20235100 | -0.00269000 |
| C | 2.43327500 | 1.74429500 | -0.00298800 |
| C | 1.17326500 | 2.40023500 | -0.00290400 |
| C | 1.15076500 | 3.78887600 | -0.00867100 |
| H | 2.05843700 | 4.37248900 | -0.01078800 |
| N | -0.00490100 | 4.46469600 | -0.01272600 |
| C | -1.18268600 | 3.82332500 | -0.00914100 |
| H | -2.07206800 | 4.43491400 | -0.01170500 |
| C | -0.04290700 | 1.69432500 | -0.00008100 |
| C | -1.23929300 | 2.43966600 | -0.00292000 |
| C | -2.50172000 | 1.79286100 | -0.00295900 |
| C | -3.56174500 | 1.21967800 | -0.00249600 |
| C | -4.83782800 | 0.59015600 | -0.00195300 |
| C | -5.98521700 | 1.39450800 | -0.00297300 |
| H | -5.86609900 | 2.46894000 | -0.00413700 |
| C | -7.24377300 | 0.82645600 | -0.00247000 |
| H | -8.12077400 | 1.45774200 | -0.00325900 |
| C | -4.98639900 | -0.80352700 | -0.00040100 |
| C | -6.25112600 | -1.37032900 | 0.00009900 |
| H | -6.35967800 | -2.44518500 | 0.00129000 |
| H | 8.37552100 | -0.87033000 | -0.00176300 |
| H | -8.35657600 | -1.00909300 | -0.00053000 |
| C | 0.00939100 | 5.93813300 | 0.02735800 |
| H | 0.95320700 | 6.29219400 | -0.37327700 |
| H | -0.10617000 | 6.26823100 | 1.05695800 |
| H | -0.80799800 | 6.31436100 | -0.57967700 |
| C | -0.09941200 | 0.21072300 | 0.00259400 |
| H | 0.89159700 | -0.23246700 | 0.00794000 |
| H | -0.66642000 | -0.13185500 | 0.87149300 |
| H | -0.65770200 | -0.13518800 | -0.87070500 |

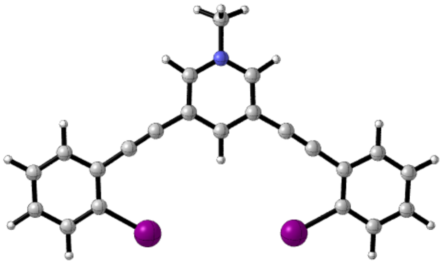

**3**

| | | | |
|---|---|---|---|
| I | 2.75586300 | -1.92596800 | -0.20922900 |
| I | -2.75611900 | -1.92585400 | 0.21184200 |
| C | 6.97251700 | -1.03856300 | 0.12306100 |
| C | -6.97254800 | -1.03823800 | -0.12278500 |
| C | 7.04376500 | 0.34474100 | 0.22171200 |

| | | | |
|---|---|---|---|
| H | 8.00132400 | 0.83653400 | 0.31559800 |
| C | 5.88312100 | 1.09297400 | 0.19949000 |
| H | 5.92016400 | 2.17066100 | 0.27568900 |
| C | 5.74538600 | -1.67593200 | 0.00194100 |
| H | 5.69826900 | -2.75240200 | -0.07502100 |
| C | 4.57850700 | -0.92946900 | -0.02058800 |
| C | 4.63562400 | 0.46841500 | 0.07894600 |
| C | 3.45634500 | 1.26168900 | 0.06136100 |
| C | 2.44882200 | 1.92079100 | 0.04634900 |
| C | 1.21145500 | 2.60917600 | 0.02224200 |
| C | 1.17180700 | 3.99949700 | 0.01503700 |
| H | 2.06654500 | 4.60252200 | 0.03078500 |
| N | 0.00028000 | 4.64955100 | -0.01392000 |
| C | -1.17130800 | 3.99930800 | -0.03506400 |
| H | -2.06602900 | 4.60219600 | -0.05622600 |
| C | 0.00020700 | 1.91577100 | -0.00052200 |
| C | -1.21103000 | 2.60899100 | -0.02907800 |
| C | -2.44832900 | 1.92047100 | -0.05259900 |
| C | -3.45586900 | 1.26136400 | -0.06616400 |
| C | -4.63530700 | 0.46829700 | -0.08197200 |
| C | -5.88264700 | 1.09285500 | -0.20412600 |
| H | -5.91943300 | 2.17037000 | -0.28284800 |
| C | -7.04346400 | 0.34484400 | -0.22472700 |
| H | -8.00089600 | 0.83663700 | -0.31989400 |
| C | -4.57852200 | -0.92935800 | 0.02086200 |
| C | -5.74557200 | -1.67559900 | -0.00001700 |
| H | -5.69871200 | -2.75189600 | 0.07947300 |
| H | 7.87663700 | -1.63147500 | 0.13970400 |
| H | -7.87680000 | -1.63098300 | -0.13814400 |
| H | 0.00024500 | 0.83401000 | 0.00151100 |
| C | -0.00074900 | 6.12505000 | 0.02706200 |
| H | 0.89689500 | 6.49072500 | -0.46033200 |
| H | -0.02308800 | 6.44985800 | 1.06450700 |
| H | -0.87675500 | 6.49050800 | -0.49838000 |

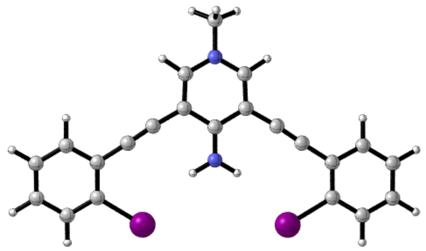

**4**

| | | | |
|---|---|---|---|
| I | 2.86727900 | -1.99847000 | 0.17059400 |
| I | -2.86683900 | -1.99932300 | -0.16177600 |

| | | | |
|---|---|---|---|
| C | 7.04386100 | -0.94404600 | -0.11333200 |
| C | -7.04448900 | -0.94356700 | 0.09988800 |
| C | 7.06222800 | 0.44183400 | -0.19214600 |
| H | 8.00105600 | 0.97104500 | -0.27121000 |
| C | 5.87459400 | 1.14693700 | -0.16972800 |
| H | 5.87353800 | 2.22614300 | -0.23076400 |
| C | 5.83971400 | -1.62623100 | -0.01182100 |
| H | 5.83032900 | -2.70468500 | 0.04942600 |
| C | 4.64851800 | -0.91972900 | 0.01084900 |
| C | 4.64821600 | 0.47836900 | -0.06797300 |
| C | 3.44617900 | 1.24115900 | -0.04869800 |
| C | 2.45257900 | 1.92232700 | -0.03480800 |
| C | 1.22143300 | 2.62530100 | -0.01772900 |
| C | 1.16914100 | 3.99525100 | -0.02348700 |
| H | 2.06905700 | 4.59137800 | -0.03645800 |
| N | -0.00024500 | 4.66662900 | -0.01465700 |
| C | -1.16947600 | 3.99537400 | 0.00539400 |
| H | -2.06946000 | 4.59142800 | 0.01479300 |
| C | -0.00013400 | 1.88695800 | 0.00064800 |
| C | -1.22168200 | 2.62536300 | 0.01171800 |
| C | -2.45292400 | 1.92248700 | 0.02599200 |
| C | -3.44656900 | 1.24137000 | 0.03920300 |
| C | -4.64866600 | 0.47863900 | 0.05735600 |
| C | -5.87544200 | 1.14767300 | 0.15087300 |
| H | -5.87462500 | 2.22716300 | 0.20667800 |
| C | -7.06316600 | 0.44268000 | 0.17186000 |
| H | -8.00230400 | 0.97225400 | 0.24454600 |
| C | -4.64865100 | -0.91983200 | -0.01459100 |
| C | -5.83994500 | -1.62622100 | 0.00665800 |
| H | -5.83031900 | -2.70496100 | -0.04927700 |
| H | 7.96917600 | -1.50298900 | -0.13056700 |
| H | -7.96986900 | -1.50243300 | 0.11604600 |
| C | 0.00114100 | 6.13510800 | 0.03918300 |
| H | 0.87503400 | 6.50748200 | -0.48627500 |
| H | 0.02282700 | 6.46663800 | 1.07512000 |
| H | -0.89383600 | 6.50771300 | -0.44920500 |
| N | -0.00010700 | 0.56845500 | 0.00334000 |
| H | 0.87276700 | 0.05403700 | -0.00279100 |
| H | -0.87297500 | 0.05403100 | 0.00996500 |

**S conformation**

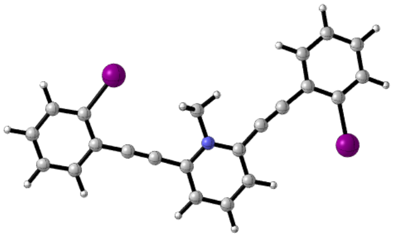

**1**

| | | | |
|---|---|---|---|
| I | 5.74625500 | -1.07999300 | -0.07925400 |
| I | -4.38615900 | 1.89831500 | -0.02774400 |
| C | 6.14187100 | 3.22140300 | 0.04918600 |
| C | -7.90783400 | -0.60258500 | -0.04608700 |
| C | 4.84034700 | 3.70398700 | 0.10111500 |
| H | 4.65391500 | 4.76752800 | 0.14009100 |
| C | 3.78237400 | 2.81699800 | 0.10294400 |
| H | 2.76319500 | 3.17552900 | 0.14361400 |
| C | 6.39008800 | 1.85648700 | -0.00110600 |
| H | 7.40596500 | 1.49108900 | -0.04193000 |
| C | 5.33272400 | 0.96155300 | 0.00094200 |
| C | 4.01294200 | 1.43606400 | 0.05337900 |
| C | 2.91066500 | 0.54234700 | 0.05861900 |
| C | 1.98274000 | -0.22733100 | 0.06354400 |
| C | 1.02706000 | -1.26700700 | 0.07069700 |
| C | 1.44929400 | -2.59231300 | 0.09393400 |
| H | 2.51198800 | -2.78232600 | 0.10645400 |
| C | 0.51626700 | -3.61070300 | 0.10097700 |
| C | -0.83333100 | -3.30510000 | 0.08454000 |
| H | -1.59332800 | -4.07064900 | 0.08948600 |
| N | -0.30304900 | -0.98276000 | 0.05432400 |
| C | -1.23581500 | -1.97750000 | 0.06062100 |
| C | -2.60083500 | -1.61725900 | 0.04102400 |
| C | -3.77376000 | -1.33684300 | 0.02267600 |
| C | -5.16536600 | -1.05708600 | 0.00021600 |
| C | -6.06553600 | -2.13181700 | 0.00186300 |
| H | -5.67348100 | -3.13901600 | 0.02110500 |
| C | -7.42712500 | -1.90548000 | -0.02116600 |
| H | -8.11298700 | -2.74035900 | -0.01986600 |
| C | -5.66581600 | 0.25248900 | -0.02460000 |
| C | -7.03246100 | 0.47493500 | -0.04773500 |
| H | -7.41507100 | 1.48496100 | -0.06697600 |
| H | 0.83880500 | -4.64245400 | 0.11953900 |
| H | 6.97560100 | 3.91004400 | 0.04734300 |
| H | -8.97304800 | -0.41759300 | -0.06450100 |
| C | -0.78254300 | 0.40831200 | 0.02701800 |
| H | 0.07076100 | 1.07415900 | 0.02909500 |
| H | -1.40860300 | 0.58113300 | 0.89963700 |
| H | -1.38315500 | 0.55490000 | -0.86807300 |

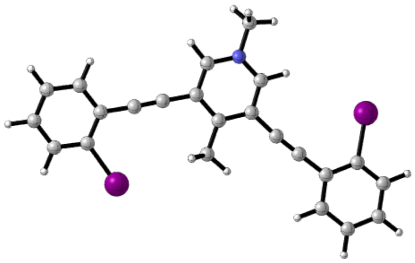

**2**

| | | | |
|---|---|---|---|
| I | 5.79000200 | 0.93546000 | -0.08199500 |
| I | -4.59277400 | -2.00793800 | -0.02066100 |
| C | 6.30674700 | -3.35165800 | 0.05611300 |
| C | -8.02091200 | 0.61866000 | -0.04139400 |
| C | 5.01946100 | -3.86952900 | 0.10558600 |
| H | 4.86262400 | -4.93788300 | 0.14613500 |
| C | 3.93551200 | -3.01305800 | 0.10288000 |
| H | 2.92693200 | -3.40050200 | 0.14120800 |
| C | 6.51386800 | -1.98011800 | 0.00374900 |
| H | 7.51819700 | -1.58396000 | -0.03522700 |
| C | 5.42881100 | -1.11896600 | 0.00128000 |
| C | 4.12295700 | -1.62673400 | 0.05104300 |
| C | 2.99205000 | -0.76459000 | 0.05128700 |
| C | 2.04749800 | -0.01843900 | 0.05208400 |
| C | 1.01661100 | 0.95750000 | 0.05332700 |
| C | 1.40071600 | 2.29295100 | 0.06518200 |
| H | 2.44205700 | 2.57948400 | 0.07528600 |
| N | 0.49131300 | 3.27362500 | 0.06368900 |
| C | -0.82261700 | 3.00050800 | 0.05240200 |
| H | -1.49633000 | 3.84386600 | 0.05291400 |
| C | -0.34904500 | 0.63272600 | 0.04023300 |
| C | -1.27836000 | 1.69310300 | 0.04027000 |
| C | -2.67430200 | 1.44075900 | 0.02420500 |
| C | -3.85553800 | 1.20290800 | 0.00999200 |
| C | -5.25987800 | 0.97391100 | -0.00710600 |
| C | -6.12233600 | 2.07829600 | -0.00905000 |
| H | -5.69478300 | 3.07109100 | 0.00284100 |
| C | -7.49186200 | 1.90263100 | -0.02609100 |
| H | -8.14622700 | 2.76254500 | -0.02750600 |
| C | -5.80915500 | -0.31541400 | -0.02250600 |
| C | -7.18410000 | -0.48818700 | -0.03957200 |
| H | -7.60175000 | -1.48442700 | -0.05141100 |
| H | 7.16014500 | -4.01565500 | 0.05772400 |
| H | -9.09216500 | 0.47230500 | -0.05490800 |
| C | 0.92869400 | 4.68033100 | 0.11960900 |

| | | | |
|---|---|---|---|
| H | 1.95727500 | 4.74233800 | -0.21875800 |
| H | 0.85116500 | 5.03669900 | 1.14393800 |
| H | 0.29331300 | 5.27238700 | -0.53175900 |
| C | -0.83242600 | -0.77226900 | 0.02310500 |
| H | -0.00937700 | -1.47971500 | 0.02721400 |
| H | -1.47973400 | -0.94930100 | 0.88497600 |
| H | -1.45796100 | -0.93523100 | -0.85749300 |

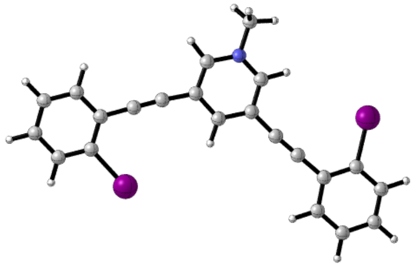

**3**

| | | | |
|---|---|---|---|
| I | 5.71398800 | 0.84535100 | 0.01895100 |
| I | -4.29002900 | -1.99341300 | 0.02460800 |
| C | 5.99092800 | -3.46608400 | -0.02654200 |
| C | -8.00732300 | 0.20792700 | -0.01549300 |
| C | 4.67647200 | -3.91315600 | -0.04009100 |
| H | 4.46040700 | -4.97179200 | -0.05306300 |
| C | 3.64166000 | -2.99825000 | -0.03706600 |
| H | 2.61242400 | -3.32852400 | -0.04759000 |
| C | 6.27497900 | -2.10729900 | -0.00988500 |
| H | 7.30040100 | -1.76737900 | 0.00065100 |
| C | 5.23979200 | -1.18697100 | -0.00684400 |
| C | 3.90734500 | -1.62353900 | -0.02050300 |
| C | 2.82426500 | -0.70338900 | -0.01839600 |
| C | 1.91503100 | 0.08556000 | -0.01683700 |
| C | 0.91523500 | 1.08876800 | -0.01437200 |
| C | 1.31002400 | 2.42522700 | -0.01599400 |
| H | 2.35131700 | 2.71074300 | -0.01727500 |
| N | 0.40055200 | 3.40578100 | -0.01679300 |
| C | -0.91713200 | 3.15092800 | -0.01465400 |
| H | -1.57952100 | 4.00305000 | -0.01482500 |
| C | -0.44872300 | 0.80537900 | -0.01278000 |
| C | -1.38557100 | 1.84318100 | -0.01255400 |
| C | -2.77595000 | 1.57339600 | -0.01308200 |
| C | -3.94000100 | 1.26553400 | -0.01294800 |
| C | -5.31075100 | 0.88925500 | -0.01315500 |
| C | -6.29643400 | 1.88390700 | -0.02809900 |
| H | -5.98953500 | 2.92038200 | -0.03882700 |
| C | -7.63551200 | 1.54580600 | -0.02931500 |

| | | | |
|---|---|---|---|
| H | -8.38797800 | 2.32119200 | -0.04108400 |
| C | -5.70062300 | -0.45779300 | 0.00082300 |
| C | -7.04519400 | -0.79227400 | -0.00037500 |
| H | -7.34222500 | -1.83088900 | 0.01052500 |
| H | 6.80632000 | -4.17626800 | -0.02883600 |
| H | -9.05365300 | -0.06490500 | -0.01634200 |
| H | -0.79182300 | -0.21989000 | -0.01376500 |
| C | 0.84730600 | 4.81213300 | 0.02159200 |
| H | 1.87880400 | 4.86271000 | -0.30913900 |
| H | 0.76283900 | 5.18122300 | 1.04076100 |
| H | 0.21956500 | 5.39767700 | -0.64282300 |

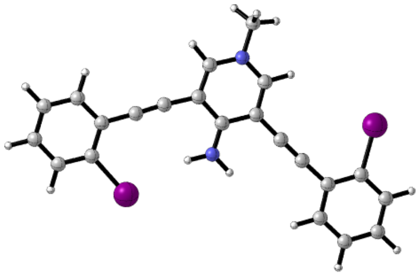

**4**

| | | | |
|---|---|---|---|
| I | 5.78444700 | 0.79908100 | -0.00136700 |
| I | -4.27001200 | -2.00308100 | 0.00830600 |
| C | 5.89371000 | -3.51982100 | -0.00325000 |
| C | -7.96148200 | 0.23242600 | -0.01019000 |
| C | 4.56275400 | -3.91473400 | -0.00346700 |
| H | 4.30479400 | -4.96407300 | -0.00393300 |
| C | 3.56510400 | -2.95947100 | -0.00315300 |
| H | 2.52483000 | -3.25404500 | -0.00344600 |
| C | 6.22814300 | -2.17305800 | -0.00269600 |
| H | 7.26562300 | -1.87183800 | -0.00256600 |
| C | 5.22967600 | -1.21206200 | -0.00231300 |
| C | 3.88140100 | -1.59584600 | -0.00254500 |
| C | 2.83457300 | -0.63114900 | -0.00223100 |
| C | 1.97423900 | 0.21191400 | -0.00202500 |
| C | 0.98303900 | 1.22588000 | -0.00134300 |
| C | 1.33805800 | 2.55139400 | -0.00740800 |
| H | 2.37604100 | 2.85017300 | -0.00879400 |
| N | 0.41847100 | 3.53656700 | -0.01336200 |
| C | -0.89617100 | 3.24022000 | -0.00860700 |
| H | -1.58113400 | 4.07456700 | -0.01090700 |
| C | -0.39913900 | 0.88237100 | 0.00174600 |
| C | -1.34921000 | 1.94512200 | -0.00263700 |
| C | -2.73272800 | 1.63440900 | -0.00467400 |
| C | -3.88214900 | 1.27333800 | -0.00556300 |
| C | -5.25466200 | 0.89466600 | -0.00692300 |

| | | | |
|---|---|---|---|
| C | -6.23511800 | 1.89464700 | -0.01362700 |
| H | -5.92125600 | 2.92898700 | -0.01753100 |
| C | -7.57697000 | 1.56631200 | -0.01526200 |
| H | -8.32239500 | 2.34860700 | -0.02051900 |
| C | -5.66081900 | -0.44536700 | -0.00187700 |
| C | -7.00642200 | -0.77416000 | -0.00349100 |
| H | -7.31038400 | -1.81083300 | 0.00045900 |
| H | 6.68099800 | -4.26091100 | -0.00354500 |
| H | -9.00972500 | -0.03236200 | -0.01143100 |
| C | 0.85281500 | 4.93997700 | 0.03903800 |
| H | 1.80317100 | 5.03481100 | -0.47688000 |
| H | 0.96073000 | 5.25330800 | 1.07517700 |
| H | 0.11248400 | 5.55796500 | -0.45922400 |
| N | -0.79045600 | -0.38036300 | 0.00480400 |
| H | -0.10219700 | -1.11690400 | 0.00553400 |
| H | -1.77457900 | -0.62092400 | 0.00470000 |

**W conformation**

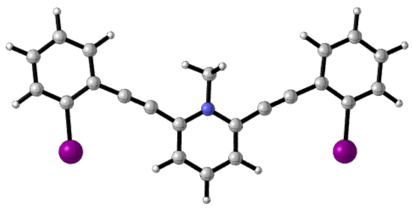

**1**

| | | | |
|---|---|---|---|
| I | -5.77608900 | -1.46336400 | 0.08285400 |
| I | 5.80197600 | -1.45819000 | -0.08828000 |
| C | -7.36415300 | 2.54998400 | -0.12624700 |
| C | 7.32349600 | 2.58059000 | 0.11976800 |
| C | -6.25050700 | 3.37931300 | -0.16704500 |
| H | -6.37053500 | 4.45173700 | -0.22094800 |
| C | -4.98572900 | 2.82657300 | -0.13893800 |
| H | -4.10834300 | 3.45753000 | -0.17110200 |
| C | -7.21853300 | 1.17124000 | -0.05656400 |
| H | -8.09080000 | 0.53462400 | -0.02510800 |
| C | -5.95207500 | 0.61066400 | -0.02750300 |
| C | -4.81872900 | 1.43736000 | -0.06956700 |
| C | -3.50925900 | 0.89075300 | -0.04643800 |
| C | -2.40192700 | 0.41430800 | -0.02843600 |
| C | -1.19064000 | -0.31169400 | -0.00975000 |
| C | -1.21831100 | -1.70180700 | -0.00772800 |
| H | -2.18321300 | -2.18601100 | -0.01969500 |
| C | -0.03388400 | -2.41397500 | 0.00814700 |
| C | 1.17284300 | -1.73805000 | 0.02242600 |
| H | 2.12315700 | -2.24996800 | 0.03531300 |

| | | | |
|---|---|---|---|
| N | 0.00486300 | 0.33929400 | 0.00547800 |
| C | 1.18018500 | -0.35003400 | 0.02153500 |
| C | 2.39640300 | 0.36550600 | 0.03738600 |
| C | 3.49596400 | 0.86041200 | 0.05178400 |
| C | 4.79685100 | 1.42622100 | 0.07069500 |
| C | 4.94116900 | 2.81820900 | 0.14034500 |
| H | 4.05321100 | 3.43404900 | 0.17572800 |
| C | 6.19660200 | 3.39174700 | 0.16467400 |
| H | 6.29931500 | 4.46594700 | 0.21883300 |
| C | 5.94345200 | 0.61781400 | 0.02414700 |
| C | 7.20042000 | 1.19967900 | 0.04956500 |
| H | 8.08307600 | 0.57772700 | 0.01480300 |
| H | -0.05076600 | -3.49504200 | 0.00931400 |
| H | -8.35799700 | 2.97554700 | -0.14827800 |
| H | 8.31033200 | 3.02233000 | 0.13892000 |
| C | 0.06673400 | 1.80909500 | 0.00526500 |
| H | -0.94111500 | 2.20349000 | -0.00881700 |
| H | 0.61518600 | 2.13537500 | -0.87503600 |
| H | 0.59022700 | 2.13656300 | 0.90020900 |

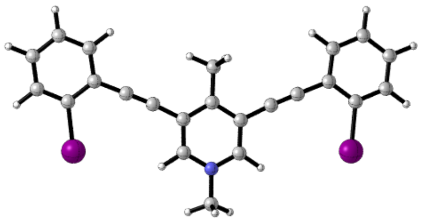

**2**

| | | | |
|---|---|---|---|
| I | -5.80649800 | -1.37804500 | -0.07802500 |
| I | 5.83019000 | -1.37696300 | 0.08090200 |
| C | -7.49910800 | 2.59258500 | 0.11234300 |
| C | 7.46843600 | 2.61617900 | -0.10993900 |
| C | -6.40728600 | 3.44938400 | 0.15267200 |
| H | -6.55460000 | 4.51869100 | 0.20270100 |
| C | -5.12745500 | 2.92997100 | 0.12895100 |
| H | -4.26690500 | 3.58366700 | 0.16033100 |
| C | -7.31532300 | 1.21826500 | 0.04765900 |
| H | -8.16933900 | 0.55739700 | 0.01631700 |
| C | -6.03305000 | 0.69454700 | 0.02343700 |
| C | -4.92070200 | 1.54687500 | 0.06457100 |
| C | -3.59324000 | 1.03703500 | 0.04482700 |
| C | -2.47557600 | 0.59019900 | 0.02940700 |
| C | -1.20625100 | -0.04526100 | 0.01145000 |
| C | -1.18480900 | -1.43473000 | 0.00167200 |
| H | -2.09715000 | -2.01269500 | 0.00932700 |
| N | -0.02892800 | -2.10861100 | -0.01855600 |
| C | 1.14767900 | -1.46531700 | -0.02815800 |
| H | 2.04285600 | -2.06959400 | -0.04363200 |

| | | | |
|---|---|---|---|
| C | 0.00615400 | 0.66285600 | 0.00072700 |
| C | 1.20089600 | -0.08089600 | -0.01929900 |
| C | 2.47334400 | 0.54495000 | -0.03442400 |
| C | 3.58425700 | 1.00928400 | -0.04752800 |
| C | 4.90484600 | 1.53574600 | -0.06506800 |
| C | 5.09271300 | 2.92174200 | -0.12927500 |
| H | 4.22306000 | 3.56325700 | -0.16177300 |
| C | 6.36527800 | 3.45845900 | -0.15152600 |
| H | 6.49841400 | 4.52962400 | -0.20144400 |
| C | 6.02839000 | 0.69790100 | -0.02229500 |
| C | 7.30331100 | 1.23950300 | -0.04525600 |
| H | 8.16630700 | 0.59044500 | -0.01287300 |
| H | -8.50395100 | 2.99149200 | 0.13075700 |
| H | 8.46785200 | 3.02858200 | -0.12730400 |
| C | -0.03973500 | -3.58260000 | 0.01540600 |
| H | -0.99328800 | -3.93586000 | -0.36207800 |
| H | 0.10274800 | -3.91574200 | 1.04058200 |
| H | 0.76323200 | -3.95397200 | -0.61349000 |
| C | 0.05803300 | 2.15006800 | 0.00634400 |
| H | -0.93604100 | 2.58476900 | 0.03270900 |
| H | 0.63193500 | 2.49379900 | 0.86886600 |
| H | 0.58688800 | 2.50136200 | -0.88176900 |

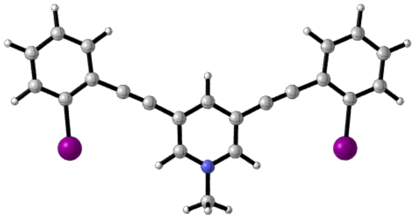

**3**

| | | | |
|---|---|---|---|
| I | -5.83687300 | -1.29416400 | 0.07788300 |
| I | 5.83740400 | -1.29406600 | -0.07615100 |
| C | -7.45666500 | 2.70671800 | -0.10712400 |
| C | 7.45610600 | 2.70710800 | 0.11143400 |
| C | -6.34977400 | 3.54415400 | -0.14640300 |
| H | -6.47815800 | 4.61596700 | -0.19444200 |
| C | -5.07943700 | 3.00211600 | -0.12420800 |
| H | -4.20668400 | 3.63923200 | -0.15479900 |
| C | -7.29826600 | 1.32898700 | -0.04477300 |
| H | -8.16434600 | 0.68397300 | -0.01413400 |
| C | -6.02594400 | 0.78192300 | -0.02191100 |
| C | -4.89868900 | 1.61502700 | -0.06239900 |
| C | -3.58137400 | 1.08188300 | -0.04507700 |
| C | -2.47125400 | 0.61649800 | -0.03234200 |
| C | -1.20972800 | -0.02741200 | -0.01810800 |

| | | | |
|---|---|---|---|
| C | -1.17062700 | -1.41856900 | -0.02658000 |
| H | -2.07111000 | -2.01443100 | -0.04118400 |
| N | -0.00004100 | -2.06966700 | -0.01794600 |
| C | 1.17050100 | -1.41871100 | 0.00098600 |
| H | 2.07100400 | -2.01468600 | 0.00767600 |
| C | -0.00009400 | 0.66739500 | 0.00055600 |
| C | 1.20955400 | -0.02756400 | 0.01087200 |
| C | 2.47110400 | 0.61621800 | 0.02815600 |
| C | 3.58118300 | 1.08163100 | 0.04331300 |
| C | 4.89839100 | 1.61494800 | 0.06299200 |
| C | 5.07880600 | 3.00205100 | 0.12555700 |
| H | 4.20589400 | 3.63899600 | 0.15514600 |
| C | 6.34900700 | 3.54432400 | 0.14957100 |
| H | 6.47712500 | 4.61614700 | 0.19809900 |
| C | 6.02585800 | 0.78207000 | 0.02375400 |
| C | 7.29804300 | 1.32936800 | 0.04846800 |
| H | 8.16428200 | 0.68452200 | 0.01883200 |
| H | -8.45418600 | 3.12371500 | -0.12443900 |
| H | 8.45352700 | 3.12428500 | 0.13015600 |
| H | -0.00007700 | 1.74796200 | 0.00470200 |
| C | -0.00055800 | -3.54545400 | 0.01840300 |
| H | -0.88219500 | -3.90862700 | -0.49912100 |
| H | -0.01250600 | -3.87267900 | 1.05522000 |
| H | 0.89270000 | -3.90876500 | -0.47869000 |

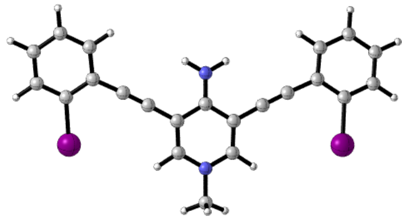

**4**

| | | | |
|---|---|---|---|
| I | -5.87679700 | -1.28118000 | -0.30150500 |
| I | 5.87522000 | -1.28122000 | 0.30569400 |
| C | -7.35545900 | 2.71373500 | 0.42023700 |
| C | 7.35723200 | 2.71226500 | -0.41757900 |
| C | -6.21991800 | 3.49189700 | 0.60062400 |
| H | -6.30967000 | 4.55013400 | 0.79993500 |
| C | -4.97029000 | 2.90784800 | 0.52626600 |
| H | -4.07839900 | 3.50197300 | 0.66940100 |
| C | -7.24401700 | 1.35419300 | 0.16557100 |
| H | -8.13115300 | 0.75362700 | 0.02634500 |
| C | -5.99205100 | 0.76522900 | 0.08909200 |
| C | -4.83646700 | 1.53845300 | 0.26965500 |

| | | | |
|---|---|---|---|
| C | -3.53946300 | 0.95558700 | 0.20148900 |
| C | -2.46197600 | 0.42035300 | 0.14271800 |
| C | -1.21624000 | -0.25319000 | 0.06782500 |
| C | -1.16667500 | -1.62560600 | 0.05294700 |
| H | -2.07004700 | -2.21563000 | 0.10272000 |
| N | -0.00088800 | -2.29558200 | -0.02483300 |
| C | 1.16576900 | -1.62542700 | -0.08659600 |
| H | 2.06868600 | -2.21531300 | -0.14538200 |
| C | 0.00052500 | 0.47986500 | 0.00191200 |
| C | 1.21655200 | -0.25300600 | -0.07775400 |
| C | 2.46238400 | 0.42071000 | -0.14969200 |
| C | 3.54013600 | 0.95567700 | -0.20585700 |
| C | 4.83749800 | 1.53810500 | -0.27136400 |
| C | 4.97237900 | 2.90713300 | -0.52937900 |
| H | 4.08103000 | 3.50130400 | -0.67566500 |
| C | 6.22239700 | 3.49063500 | -0.60152300 |
| H | 6.31301000 | 4.54857300 | -0.80202400 |
| C | 5.99235500 | 0.76465200 | -0.08722000 |
| C | 7.24471400 | 1.35306400 | -0.16158100 |
| H | 8.13129700 | 0.75231800 | -0.01964800 |
| H | -8.33725800 | 3.16302700 | 0.47736000 |
| H | 8.33932800 | 3.16111800 | -0.47301600 |
| C | 0.00185100 | -3.76545900 | 0.01929400 |
| H | -0.91190400 | -4.13373400 | -0.43633300 |
| H | 0.06303600 | -4.10124600 | 1.05212100 |
| H | 0.85549700 | -4.13352000 | -0.54107800 |
| N | 0.00096400 | 1.80563000 | 0.01079600 |
| H | -0.86893400 | 2.31195400 | 0.05966200 |
| H | 0.87111500 | 2.31202300 | -0.03266700 |

## Iodide Complexes

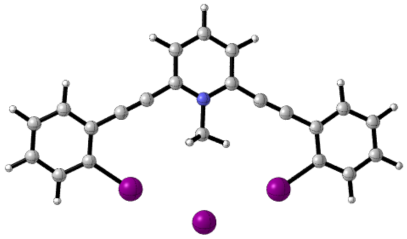

**1•I⁻**

| | | | |
|---|---|---|---|
| I | 3.14306200 | -1.28951400 | -0.00020300 |
| I | -3.10706400 | -1.27344400 | -0.00013700 |
| C | 7.26483200 | 0.15776300 | -0.00061900 |
| C | -7.25822600 | 0.08919500 | -0.00061200 |
| C | 7.14645600 | 1.54395400 | -0.00034200 |

| | | | |
|---|---|---|---|
| H | 8.02873200 | 2.16821900 | -0.00033700 |
| C | 5.89198200 | 2.11897600 | -0.00007300 |
| H | 5.77722500 | 3.19440200 | 0.00013800 |
| C | 6.13674900 | -0.64877600 | -0.00060900 |
| H | 6.24041200 | -1.72499100 | -0.00081900 |
| C | 4.86600100 | -0.08726300 | -0.00032200 |
| C | 4.74526700 | 1.31267400 | -0.00006900 |
| C | 3.46679000 | 1.93476900 | 0.00016200 |
| C | 2.37986200 | 2.45820600 | 0.00030400 |
| C | 1.16091600 | 3.17083300 | 0.00031200 |
| C | 1.18150700 | 4.56329100 | 0.00026200 |
| H | 2.14206400 | 5.05349800 | 0.00025900 |
| C | -0.00263900 | 5.27231400 | 0.00019900 |
| C | -1.20272000 | 4.58939900 | 0.00016200 |
| H | -2.15350900 | 5.09840500 | 0.00007700 |
| N | -0.02934300 | 2.51046400 | 0.00035200 |
| C | -1.20514900 | 3.19878100 | 0.00023500 |
| C | -2.42305000 | 2.48833900 | 0.00019300 |
| C | -3.49905300 | 1.94196500 | 0.00006600 |
| C | -4.76436100 | 1.29622300 | -0.00012700 |
| C | -5.92754300 | 2.07931100 | -0.00018200 |
| H | -5.83490400 | 3.15686200 | -0.00003600 |
| C | -7.16924500 | 1.47801800 | -0.00041900 |
| H | -8.06462500 | 2.08332900 | -0.00045600 |
| C | -4.85469100 | -0.10606200 | -0.00030200 |
| C | -6.11389700 | -0.69368100 | -0.00055400 |
| H | -6.19509600 | -1.77183800 | -0.00069900 |
| H | 0.00966700 | 6.35336600 | 0.00016900 |
| H | 8.24536000 | -0.29961700 | -0.00084100 |
| H | -8.22909000 | -0.38840600 | -0.00080700 |
| C | -0.08159500 | 1.02745700 | 0.00067400 |
| H | 0.92494400 | 0.62814700 | 0.00037500 |
| H | -0.62105600 | 0.68955300 | -0.88177100 |
| H | -0.62037100 | 0.69001100 | 0.88372000 |
| I | 0.00412400 | -2.67512300 | 0.00050100 |

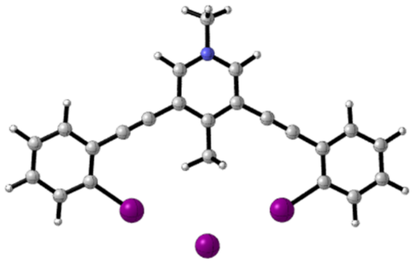

**2•I⁻** (one imaginary frequency)

| | | | |
|---|---|---|---|
| I | 3.11071500 | -1.42585600 | 0.00027900 |
| I | -3.15139200 | -1.44712500 | 0.00046500 |
| C | 7.27788400 | -0.12737800 | -0.00891700 |
| C | -7.28589300 | -0.05004200 | -0.00905500 |
| C | 7.21688000 | 1.26221900 | -0.01095500 |
| H | 8.12406200 | 1.84998100 | -0.01340700 |
| C | 5.98664400 | 1.88806700 | -0.00982200 |
| H | 5.91727600 | 2.96748000 | -0.01135900 |
| C | 6.11617000 | -0.88363200 | -0.00577400 |
| H | 6.17344500 | -1.96336500 | -0.00418300 |
| C | 4.86836300 | -0.27043800 | -0.00461900 |
| C | 4.80587000 | 1.13307900 | -0.00669000 |
| C | 3.55379900 | 1.81190800 | -0.00561500 |
| C | 2.49665000 | 2.39170200 | -0.00464500 |
| C | 1.23300900 | 3.03399400 | -0.00254000 |
| C | 1.18081900 | 4.41703600 | -0.00451500 |
| H | 2.07339400 | 5.02359200 | -0.00506500 |
| C | -1.15310000 | 4.39301100 | -0.00435400 |
| H | -2.05797500 | 4.98085300 | -0.00469900 |
| C | -1.18230700 | 3.00653500 | -0.00267900 |
| C | -2.44648300 | 2.35979600 | -0.00487700 |
| C | -3.51507000 | 1.80223800 | -0.00581200 |
| C | -4.78261800 | 1.15003600 | -0.00684800 |
| C | -5.94490600 | 1.93256000 | -0.01015600 |
| H | -5.84948900 | 3.00999200 | -0.01184700 |
| C | -7.19054300 | 1.33719400 | -0.01128600 |
| H | -8.08282700 | 1.94735500 | -0.01388100 |
| C | -4.88067600 | -0.25144000 | -0.00457300 |
| C | -6.14259500 | -0.83433800 | -0.00572400 |
| H | -6.22620400 | -1.91234000 | -0.00399300 |
| H | 8.23805900 | -0.62609600 | -0.00977600 |
| H | -8.25784200 | -0.52535800 | -0.00991300 |
| C | 0.07253900 | 0.80893800 | -0.00315300 |
| H | 0.63261600 | 0.44167600 | -0.86790600 |
| H | -0.91557200 | 0.36390800 | -0.00432000 |
| H | 0.63074500 | 0.44035900 | 0.86232100 |
| I | -0.00243400 | -2.91801400 | 0.00895600 |
| C | 0.03237500 | 2.28776000 | -0.00180300 |
| N | 0.00629300 | 5.06566600 | -0.00659800 |
| C | -0.00563100 | 6.53377900 | 0.04531000 |
| H | 0.85072300 | 6.91364600 | -0.50286900 |
| H | -0.91945700 | 6.89608200 | -0.41416800 |
| H | 0.04280100 | 6.85981300 | 1.08180800 |

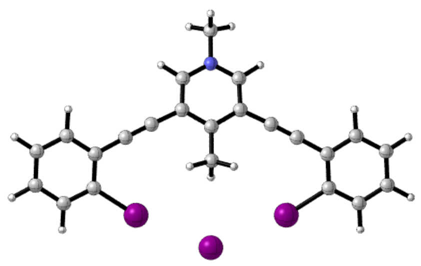

**2•I⁻** --Tridentate structure with CH hydrogen bonding from central methyl group to iodide

| | | | |
|---|---|---|---|
| I | 3.10593600 | -1.40774900 | 0.01426600 |
| I | -3.10595100 | -1.40775300 | 0.01295400 |
| C | 7.21783500 | -0.15369200 | 0.77846200 |
| C | -7.21755600 | -0.15417800 | 0.77946500 |
| C | 7.15764000 | 1.23461100 | 0.82635500 |
| H | 8.05251600 | 1.81540400 | 1.00058600 |
| C | 5.94400300 | 1.86954700 | 0.64984800 |
| H | 5.87589600 | 2.94842800 | 0.68550600 |
| C | 6.07177600 | -0.90149900 | 0.55445300 |
| H | 6.12836000 | -1.98055500 | 0.51564900 |
| C | 4.84228400 | -0.27879400 | 0.37553900 |
| C | 4.78009200 | 1.12377400 | 0.42641300 |
| C | 3.53790400 | 1.79993500 | 0.25040500 |
| C | 2.47467100 | 2.34512800 | 0.09567600 |
| C | 1.20695800 | 2.94902900 | -0.10941400 |
| C | 1.16599700 | 4.25625400 | -0.56241500 |
| H | 2.06592800 | 4.81824200 | -0.75930500 |
| N | 0.00002000 | 4.88194200 | -0.78142100 |
| C | -1.16609400 | 4.25623000 | -0.56236500 |
| H | -2.06587300 | 4.81851400 | -0.75933500 |
| C | -0.00003900 | 2.25083000 | 0.12861800 |
| C | -1.20714700 | 2.94915300 | -0.10936000 |
| C | -2.47480300 | 2.34518400 | 0.09586200 |
| C | -3.53794000 | 1.79985000 | 0.25074700 |
| C | -4.78003000 | 1.12355300 | 0.42693900 |
| C | -5.94386500 | 1.86917400 | 0.65127100 |
| H | -5.87577700 | 2.94803900 | 0.68743200 |
| C | -7.15739500 | 1.23410300 | 0.82802500 |
| H | -8.05221200 | 1.81477400 | 1.00296700 |
| C | -4.84218400 | -0.27899200 | 0.37538800 |
| C | -6.07157400 | -0.90183000 | 0.55454900 |
| H | -6.12813900 | -1.98086900 | 0.51522900 |
| H | 8.16485300 | -0.65877000 | 0.91510200 |
| H | -8.16449200 | -0.65936000 | 0.91629200 |
| C | -0.00112600 | 6.28444900 | -1.21681600 |
| H | 0.89314900 | 6.47330700 | -1.80195500 |
| H | -0.01852000 | 6.93535800 | -0.34548400 |
| H | -0.87861200 | 6.46323700 | -1.83006600 |

| | | | |
|---|---|---|---|
| C | -0.00009800 | 0.84814400 | 0.58829800 |
| H | 0.89835300 | 0.61429700 | 1.15547300 |
| H | -0.89868800 | 0.61426000 | 1.15520100 |
| H | 0.00014200 | 0.15693900 | -0.27237200 |
| I | 0.00014900 | -2.77949100 | -0.66432600 |

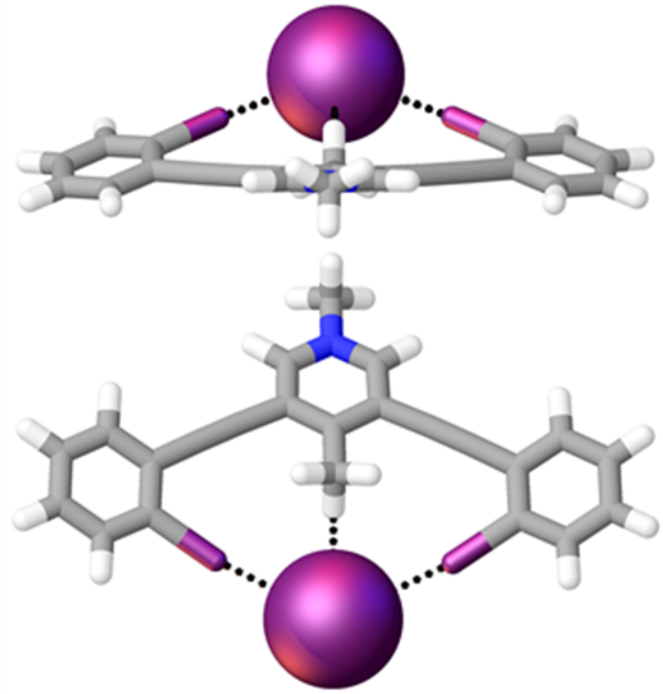

Figure 35S Tridentate binding mode of **2•I⁻** obtained from DFT evaluations, shown from two different perspectives.

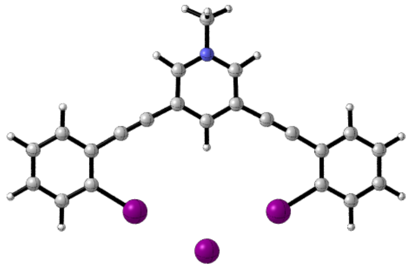

**3•I⁻**

| | | | |
|---|---|---|---|
| I | 2.95714700 | -1.35070800 | -0.01791900 |
| I | -2.95710300 | -1.35067800 | -0.01799700 |
| C | 7.16227500 | -0.21086700 | -0.36852100 |
| C | -7.16228900 | -0.21094400 | -0.36838200 |
| C | 7.15937300 | 1.18063500 | -0.36382100 |
| H | 8.08729600 | 1.73017900 | -0.43707800 |
| C | 5.95986100 | 1.85578600 | -0.26521200 |
| H | 5.93402600 | 2.93719500 | -0.26053100 |
| C | 5.97506400 | -0.92032000 | -0.27415600 |

| | | | |
|---|---|---|---|
| H | 5.98953800 | -2.00158100 | -0.27718700 |
| C | 4.75565800 | -0.25912000 | -0.17419400 |
| C | 4.75497000 | 1.14612000 | -0.17148200 |
| C | 3.52897200 | 1.85978800 | -0.07740400 |
| C | 2.46946300 | 2.42886300 | -0.00162400 |
| C | 1.21117100 | 3.06888500 | 0.07652600 |
| C | 1.16921100 | 4.45445100 | 0.20347300 |
| H | 2.06601000 | 5.05167700 | 0.25664100 |
| N | -0.00002900 | 5.10406300 | 0.26522700 |
| C | -1.16926400 | 4.45444600 | 0.20343300 |
| H | -2.06606900 | 5.05166300 | 0.25657400 |
| C | -0.00001600 | 2.36883500 | 0.02109500 |
| C | -1.21120500 | 3.06888000 | 0.07647300 |
| C | -2.46949100 | 2.42884700 | -0.00170800 |
| C | -3.52901100 | 1.85979900 | -0.07751900 |
| C | -4.75499800 | 1.14609400 | -0.17151000 |
| C | -5.95990300 | 1.85573100 | -0.26521200 |
| H | -5.93408600 | 2.93714100 | -0.26058200 |
| C | -7.15940800 | 1.18055600 | -0.36373900 |
| H | -8.08734200 | 1.73008300 | -0.43698100 |
| C | -4.75566400 | -0.25915400 | -0.17415900 |
| C | -5.97506200 | -0.92037700 | -0.27404600 |
| H | -5.98952100 | -2.00163800 | -0.27704300 |
| H | 8.09851900 | -0.74784700 | -0.44588700 |
| H | -8.09852800 | -0.74794200 | -0.44568700 |
| H | -0.00001700 | 1.28791300 | -0.07053300 |
| C | -0.00002200 | 6.56300100 | 0.45662300 |
| H | 0.88683500 | 6.97966900 | -0.00942400 |
| H | 0.00016400 | 6.78304800 | 1.52154600 |
| H | -0.88705900 | 6.97963600 | -0.00910600 |
| I | 0.00000000 | -2.96912700 | 0.28438900 |

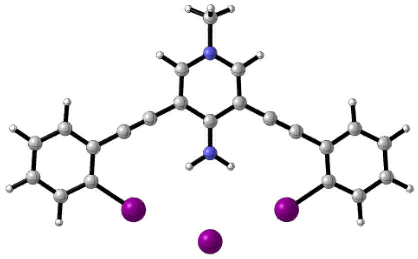

**4•I⁻**

| | | | |
|---|---|---|---|
| I | 3.17178300 | -1.44936800 | -0.00212200 |
| I | -3.17183900 | -1.44934500 | -0.00225600 |
| C | 7.32584600 | -0.11049400 | -0.03666300 |
| C | -7.32587400 | -0.11039000 | -0.03653400 |
| C | 7.24062200 | 1.27696600 | -0.03791500 |
| H | 8.13796800 | 1.87967500 | -0.04563000 |

| | | | |
|---|---|---|---|
| C | 6.00020000 | 1.88490500 | -0.02922800 |
| H | 5.91493500 | 2.96307700 | -0.03009100 |
| C | 6.17576100 | -0.88555400 | -0.02673700 |
| H | 6.24953900 | -1.96426300 | -0.02576600 |
| C | 4.92206600 | -0.28791700 | -0.01793400 |
| C | 4.83267200 | 1.11245300 | -0.01923700 |
| C | 3.56346100 | 1.76133400 | -0.01061800 |
| C | 2.48696400 | 2.30198500 | -0.00340400 |
| C | 1.22241700 | 2.94285000 | 0.00472400 |
| C | 1.16618100 | 4.31052500 | 0.00783000 |
| H | 2.06931000 | 4.90167400 | 0.00958400 |
| N | 0.00001600 | 4.98937700 | 0.00632200 |
| C | -1.16614500 | 4.31057100 | 0.00786300 |
| H | -2.06928300 | 4.90169200 | 0.00966800 |
| C | -0.00001600 | 2.18561300 | 0.00561600 |
| C | -1.22240300 | 2.94287700 | 0.00474100 |
| C | -2.48697800 | 2.30206300 | -0.00337900 |
| C | -3.56343300 | 1.76133100 | -0.01059100 |
| C | -4.83266200 | 1.11248500 | -0.01920500 |
| C | -6.00017200 | 1.88497100 | -0.02915100 |
| H | -5.91487500 | 2.96314000 | -0.03001200 |
| C | -7.24061200 | 1.27706900 | -0.03780200 |
| H | -8.13794300 | 1.87979900 | -0.04550800 |
| C | -4.92209800 | -0.28787800 | -0.01792500 |
| C | -6.17580800 | -0.88548000 | -0.02665700 |
| H | -6.24961200 | -1.96418700 | -0.02565100 |
| H | 8.29392100 | -0.59336800 | -0.04344200 |
| H | -8.29396300 | -0.59323700 | -0.04326300 |
| C | 0.00030300 | 6.45053200 | 0.07997400 |
| H | 0.88286600 | 6.83173600 | -0.42511800 |
| H | 0.00395200 | 6.77499000 | 1.11907500 |
| H | -0.88582200 | 6.83168100 | -0.41883000 |
| N | -0.00002000 | 0.87517200 | 0.00286600 |
| H | 0.85818500 | 0.32832200 | 0.00327800 |
| H | -0.85822400 | 0.32832000 | 0.00327600 |
| I | 0.00000600 | -2.76215100 | 0.03150100 |

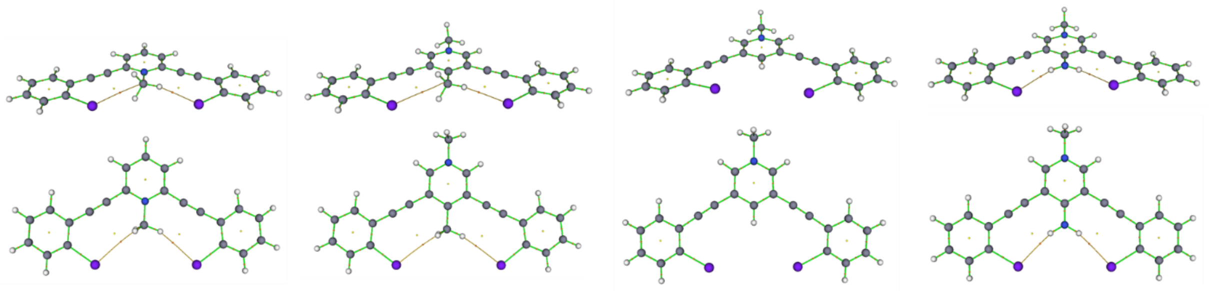


*Figure 36S. Visualization of AIM analysis. Bond path are orange lines. (3,-1) BCPs are orange dots, (3,+1) BCPs are yellow dots.*

## UV-vis Titration Details and Data

UV-vis titrations were carried out on an Agilent Cary 60 UV-Vis spectrometer equipped with a Peltier 1x1 Cell Holder Accessory which can control the liquid sample temperature at 20 °C during the titrations. Association constants were determined by non-linear regression in Bindfit[17, 18] fitting the complete spectrum simultaneously. Hamilton gas-tight micro-syringes were used during serial dilutions and titrations. The reported association constants and errors were obtained from the average and standard deviation of three repeated titrations. Full binding data and fitting parameters for each titration can be obtained from the Bindfit using the links found below figures below.

Considering both the solubilities and changes of absorbance, the solvent mixture used in all titrations is made by 90% spectra grade Tetrahydrofuran, 9.9% spectra grade Dimethyl sulfoxide, and 0.1% deionized water. A stock solution of **1-4 OTf** was prepared with the solvent mixture. The stock solution of each host was then used to make TBA Bromide guest solutions. An aliquot (2.0 mL) of the stock host solution was transferred to a quartz cuvette with cap and a magnetic stir bar as the starting volume. Aliquots of guest solution were added to the cuvette, after each addition and stirring for three minutes, a spectrum was recorded.

**Table S4**. Association constants for binding of TBA bromide to all receptors in 90% THF/9.9% DMSO/0.1% deionized $H_2O$ solvent system at 293 K.

| Receptor | Assay 1 ($M^{-1}$) | Assay 2 ($M^{-1}$) | Assay 3 ($M^{-1}$) | **Average ($M^{-1}$)** |
|---|---|---|---|---|
| **1-OTf** | 25516 | 27268 | 25182 | **25989 ±915** |
| **2-OTf** | 16940 | 11189 | 16181 | **14700 ±2551** |
| **3-OTf** | 12511 | 10300 | 13529 | **12113 ±1348** |
| **4-OTf** | 19417 | 16997 | 18654 | **18356 ±1010** |

**1-OTf with TBA bromide**

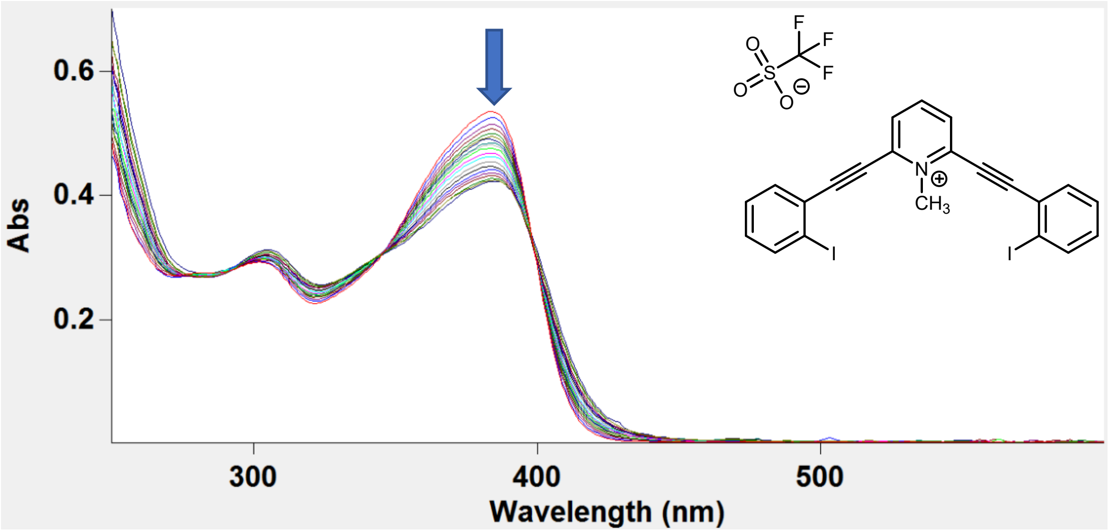

Figure 37S UV-Vis titration spectra of **1-OTf** with TBABr in THF-DMSO-$H_2O$ solvent mixture at 20 °C.

Assay 1 $K_a$= 25516 $M^{-1}$ http://app.supramolecular.org/bindfit/view/bc8348fa-c18f-4738-a858-6afee54be274
Assay 2 $K_a$= 27268 $M^{-1}$ http://app.supramolecular.org/bindfit/view/d0db7fb8-e521-422a-89f9-65a8afc351dd
Assay 3 $K_a$= 25182 $M^{-1}$ http://app.supramolecular.org/bindfit/view/80d9e25a-a4a8-4829-ab9e-ab413d6650cf

**2-OTf with TBA bromide**

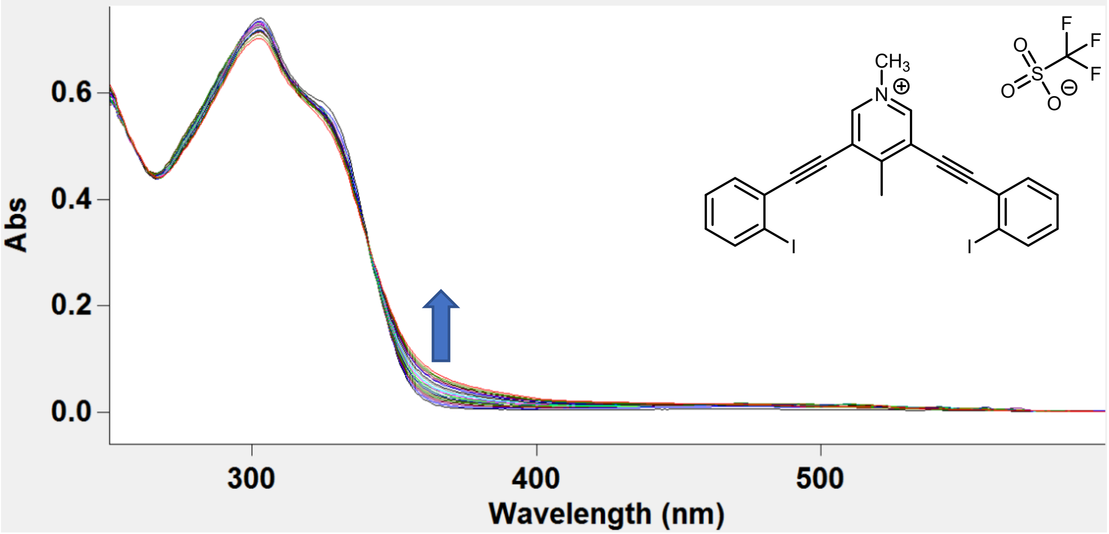

Figure 38S UV-vis titration spectra of **2-OTf** with TBABr in THF-DMSO-$H_2O$ solvent mixture at 20 °C.

Assay 1 $K_a$= 16940 $M^{-1}$ http://app.supramolecular.org/bindfit/view/67f16bc8-3f4f-40b6-a488-0e53de34ae04
Assay 2 $K_a$= 11189 $M^{-1}$ http://app.supramolecular.org/bindfit/view/f35feff3-f714-4875-92ae-a7e9fc66cf5c
Assay 3 $K_a$= 16181 $M^{-1}$ http://app.supramolecular.org/bindfit/view/0134f156-85a4-492a-90c9-51c19ab9d406

**3-OTf with TBA bromide**

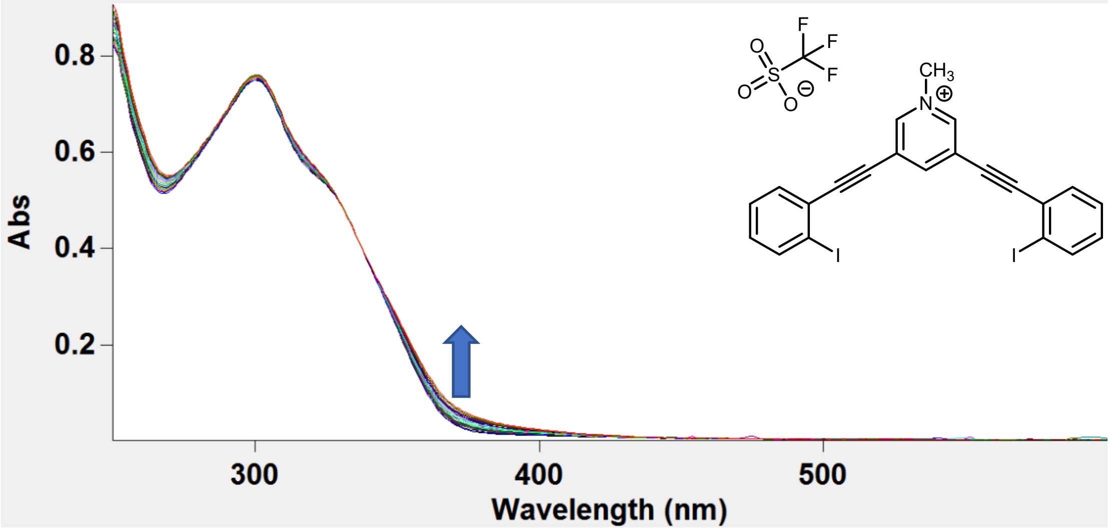


Figure 39S UV-Vis titration spectra of **3-OTf** with TBABr in THF-DMSO-$H_2O$ solvent mixture at 20 °C.

Assay 1 $K_a$= 12511 $M^{-1}$ http://app.supramolecular.org/bindfit/view/25834360-3d81-4204-abda-afb68c0b3b57
Assay 2 $K_a$= 10300 $M^{-1}$ http://app.supramolecular.org/bindfit/view/78e57c1c-3dde-48e2-8601-0ecbb7e80fc5
Assay 3 $K_a$= 13529 $M^{-1}$ http://app.supramolecular.org/bindfit/view/9a833e73-82cd-40fc-aadc-1c983a81025e

**4-OTf with TBA bromide**

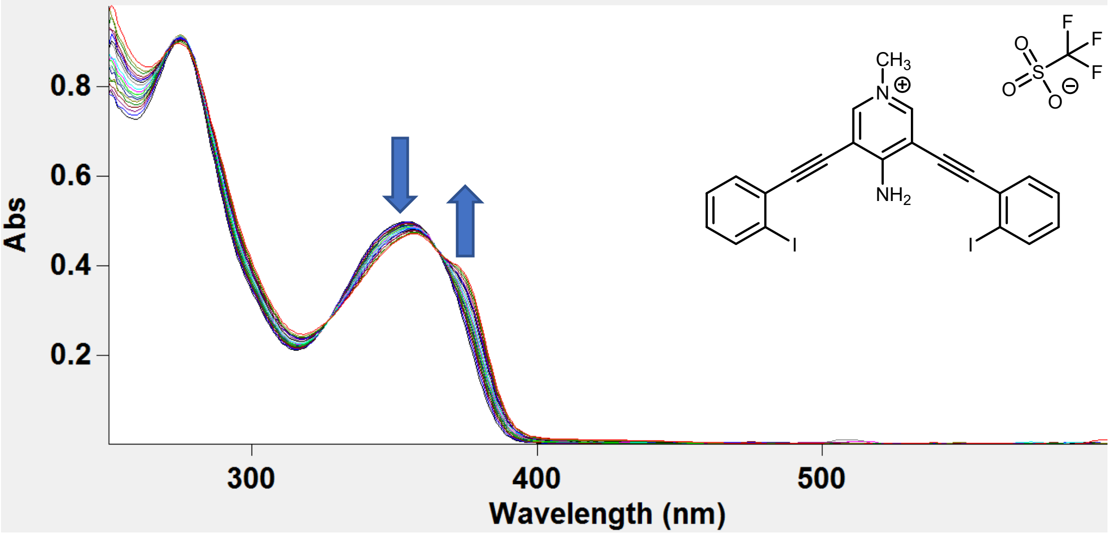


Figure 40S UV-Vis titration spectra of **4-OTf** with TBABr in THF-DMSO-$H_2O$ solvent mixture at 20 °C.

Assay 1 $K_a$= 19417 $M^{-1}$ http://app.supramolecular.org/bindfit/view/339d3d49-a9e4-4c6f-b716-2b9bc59a0c02

Assay 2 $K_a$= 16997 $M^{-1}$ http://app.supramolecular.org/bindfit/view/b56dd4e1-3cc5-494f-8772-4b704f48a1da
Assay 3 $K_a$= 18654 $M^{-1}$ http://app.supramolecular.org/bindfit/view/16b0f1e1-2ff0-4b28-b7f5-6454fab4b65f

# References:


1) Sheldrick, G. M. (1996). SADABS: Area Detector Absorption Correction; University of Göttingen, Germany.
2) Dolomanov, O.V.; Bourhis, L.J.; Gildea, R.J.; Howard, J.A.K.; Puschmann, H., (2009). J. Appl. Cryst., 42, 339-341.
3) Sheldrick, G. M. (2015).Acta Cryst.A71, 3-8.
4) Sheldrick, G. M. (2015).Acta Cryst.C71, 3-8.
5) Bruker (2016). APEX3. Bruker AXSInc., Madison, Wisconsin, USA.
6) Sheldrick, G.M. (2008). Acta Cryst. A64, 112-122.
7) Scattergood, P.A.; Roberts, J.; Omar, S.A.E.; Elliott, P.I., (2019) Inorg. Chem., 58(13), 8607-8621.
8) Hou, Y.; Cao, S.; Li, X.; Pei, Y.; Wang, B.; Wang, L.; Pei, Z., (2014) ACS Appl. Mater. Interfaces, 6(19), 16909-16917.
9) Bosch, E.; Barnes, B.L., (2000) Organometallics, 19(25), 5522-5524.
10) Riel, A.S.M,; Decato, D.A.; Sun, J.; Massena, C.J.; Jessop, M.J.; Berryman, O.B., (2018) Chem. Sci., 9, 5828-5836.
11) Zhao, Y.; Truhlar, D.G., (2008) Theor Chem Account 120, 215–241.
12) Frisch, M. J.; Trucks, G. W.; Schlegel, H. B.; Scuseria, G. E.; Robb, M. A.; Cheeseman, J. R.; Scalmani, G.; Barone, V.; Mennucci, B.; Petersson, G. A.; Nakatsuji, H.; Caricato, M.; Li, X.; Hratchian, H. P.; Izmaylov, A. F.; Bloino, J.; Zheng, G.; Sonnenberg, J. L.; Hada, M.; Ehara, M.; Toyota, K.; Fukuda, R.; Hasegawa, J.; Ishida, M.; Nakajima, T.; Honda, Y.; Kitao, O.; Nakai, H.; Vreven, T.; Montgomery, J. A. J.; Peralta, J. E.; Ogliaro, F.; Bearpark, M.; Heyd, J. J.; Brothers, E.; Kudin, K. N.; Staroverov, V. N.; Kobayashi, R.; Normand, J.; Raghavachari, K.; Rendell, A.; Burant, J. C.; Iyengar, S. S.; Tomasi, J.; Cossi, M.; Rega, N.; Millam, N. J.; Klene, M.; Knox, J. E.; Cross, J. B.; Bakken, V.; Adamo, C.; Jaramillo, J.; Gomperts, R.; Stratmann, R. E.; Yazyev, O.; Austin, A. J.; Cammi, R.; Pomelli, C.; Ochterski, J. W.; Martin, R. L.; Morokuma, K.; Zakrzewski, V. G.; Voth, G. A.; Salvador, P.; Dannenberg, J. J.; Dapprich, S.; Daniels, A. D.; Farkas, O.; Foresman, J. B.; Ortiz, J. V.; Cioslowski, J.; Fox, D. J. Gaussian, Inc.: Wallingford, CT 2009.
13) Weigend, F.; Ahlrichs, R., (2005) Phys. Chem. Chem. Phys. 7, 3297.
14) Peterson, K.A., (2003) J. Chem. Phys. 119, 11113-11123.
15) Pritchard, B.P.; Altarawy, D.; Didier, B.; Gibsom, T.D.; Windus, T.L., (2019) J. Chem. Inf. Model. 59, 4814-4820
16) Boys, F.; Bernardi, F., (1970) Mol. Phys. 19, 553.
17) http://supramolecular.org/
18) Hibbert, D.B.; Thordarson, P.; (2016) Chem. Commun., 52, 12792-12805.
